# Comprehensive Comparative Genomic and Transcriptomic Analyses of the Legume Genes Controlling the Nodulation Process

**DOI:** 10.3389/fpls.2016.00034

**Published:** 2016-01-29

**Authors:** Zhenzhen Qiao, Lise Pingault, Mehrnoush Nourbakhsh-Rey, Marc Libault

**Affiliations:** Department of Microbiology and Plant Biology, University of OklahomaNorman, OK, USA

**Keywords:** legume, nodulation, comparative genomic, comparative transcriptomic, paralogs, orthologs, neo-/sub-functionalization, root hair cell

## Abstract

Nitrogen is one of the most essential plant nutrients and one of the major factors limiting crop productivity. Having the goal to perform a more sustainable agriculture, there is a need to maximize biological nitrogen fixation, a feature of legumes. To enhance our understanding of the molecular mechanisms controlling the interaction between legumes and rhizobia, the symbiotic partner fixing and assimilating the atmospheric nitrogen for the plant, researchers took advantage of genetic and genomic resources developed across different legume models (e.g., *Medicago truncatula, Lotus japonicus, Glycine max*, and *Phaseolus vulgaris*) to identify key regulatory protein coding genes of the nodulation process. In this study, we are presenting the results of a comprehensive comparative genomic analysis to highlight orthologous and paralogous relationships between the legume genes controlling nodulation. Mining large transcriptomic datasets, we also identified several orthologous and paralogous genes characterized by the induction of their expression during nodulation across legume plant species. This comprehensive study prompts new insights into the evolution of the nodulation process in legume plant and will benefit the scientific community interested in the transfer of functional genomic information between species.

## Introduction

Legumes (family *Fabaceae*) are the 2nd most important crop family after grass species. Legumes are an important source of oil and proteins for human and animal consumption, and they also fix atmospheric nitrogen leading to a sturdy supply in nitrogen fertilizers, which can benefit other plant species. This unique feature of legumes is the result of their symbiotic relationship with soil bacteria involved in nodulation (e.g., *Bradyrhizobium japonicum* and *Sinorhizobium meliloti* are the *Glycine max* and *Medicago truncatula* symbiotic partners, respectively). Ultimately, upon infection of the legume by the symbiotic bacteria, a novel plant root lateral organ called a nodule is formed where rhizobia fix and convert atmospheric nitrogen into nitrite/ammonia that can be used by plants. In exchange, acting as a carbon sink, the symbionts receive plant photosynthates.

Legume nodulation is initiated by the recognition of plant flavonoids by rhizobia. In response, rhizobia secretes the Nod Factor (NF), a lipochito-oligosaccharide signal molecule which is recognized by plant lysine motif (LysM) receptor kinase, such as *Lotus japonicus* NFR1/NFR5 (Madsen et al., 2003; Radutoiu et al., 2003), *Pisum sativum* SYM10 (Madsen et al., 2003), and *M. truncatula* NFP (Amor et al., 2003) and LYK3 (Limpens et al., 2003). Downstream of the recognition of the NF, a signaling cascade is activated leading to oscillations in calcium concentration within the nucleus of the root hair cell (Miwa et al., 2006; Capoen et al., 2009; Sieberer et al., 2009). Ultimately, this molecular recognition of the two partners will lead to root hair cell deformation, curling and formation of a shepherd hook (Kijne, 1992). Upon root hair curling. rhizobia infect the root hair cell through the formation of a tube-like apoplastic compartment called infection thread (VandenBosch et al., 1989). Concomitantly, the root cortex cells are actively dividing leading to the formation of the nodule primordium (Yang et al., 1994).

Through an examination of legume mutants defective in nodulation, root hair deformation and calcium spiking, the root hair regulatory pathway activated in response to rhizobia inoculation or NF treatment was characterized. For instance, while the *M. truncatula* mutants *dmi1* and *dmi2 (does not make infections)* are not affected in their calcium flux and root hair deformation, the *nfp* (*nod factor perception*) mutant is impaired for both phenotypes suggesting that the *DMI1* and *DMI2* genes are acting downstream to *NFP* (Amor et al., 2003). As a part of this regulatory pathway, CCaMK protein, a nuclear protein sensitive to the calcium oscillations, interacts with and phosphorylates CYCLOPS, a nuclear coiled-coil transcription factor, directly inducing the expression of *NODULE INCEPTION (NIN)*, encoding a RWP-RK transcription factor (Marsh et al., 2007; Singh et al., 2014). In another model legume, *L. japonicus*, LjNIN targets two *Nuclear Factor-Y* (*NF-Y*) genes, Lj*NF-YA1* and Lj*NF-YB1*, to control nodule development (Soyano et al., 2013). NF-Y TFs play a central role during the nodulation process. For instance, in *M. truncatula*, MtNF-YA1, and MtNF-YA2 redundantly act to control the early stage of rhizobial infection via the transcriptional activation of Mt*ERN1* (*Ethylene Response Factor Required for Nodulation 1*; Laloum et al., 2014). In *Phaseolus vulgaris*, PvNF-YC1 is also controlling nodule organogenesis as well as the selection process of the symbiosis partner (Zanetti et al., 2010). More recently, Baudin et al. (2015) demonstrated that both MtNF-YA1, MtNF-YB16 and MtNF-YC2, and PvNF-YA1, PvNF-YB7, and PvNF-YC1 proteins form heterotrimers recognizing the CCAAT box of the Mt*ERN1* and Pv*ERN1* promoter sequences, respectively, (Baudin et al., 2015). Interestingly, while Mt*ERN2*, the Mt*ERN1* paralogous gene, also regulates the nodulation process, its activity does not overlap with Mt*ERN1* supporting the existence of additional regulatory pathways (Cerri et al., 2012). Together, these legume TFs control the expression of various genes regulating the infection of the plant root hair cell by rhizobia then its progression in the root cells through the development of the infection thread. MtFLOT4 and LjSYMREM1, plasma membrane microdomain proteins, seem also to play integral roles during the early stages of infection of the root hair cells by rhizobia such as the formation of the infection thread (Haney and Long, 2010; Toth et al., 2012).

While the genetic resources developed on model legumes, such as *M. truncatula* have a considerable impact on the characterization of nodulation genes, additional resources have been more recently developed taking advantage of –omic technologies. For instance, the access to large quantities of *M. truncatula* (Breakspear et al., 2014) and soybean root hair cells inoculated by rhizobia (Brechenmacher et al., 2010, 2012; Libault et al., 2010a,b; Nguyen et al., 2012; Yan et al., 2015) are now allowing a more global understanding of the molecular processes controlling the early stages of legume nodulation. In this manuscript, we are taking advantage of the knowledge gained during the past two decades and the more recent release of genomic and transcriptomic datasets to perform a comprehensive analysis of the evolution of legume protein coding genes controlling the nodulation process. Our results confirm the strong conservation of a core set of legume protein coding genes controlling nodulation between species and also highlight the divergence of a subset of the paralogs. Interestingly, the nodulation genes are also characterized by their high density along the legume chromosomes suggesting that these genes share the same biological function and are physically co-localized on the chromosomes. This study represents a new resource to better understand the evolution of legume nodulation, maximize the transfer of the scientific information between legumes and to open perspectives regarding the role and the conservation of gene modules in controlling the nodulation process.

## Materials and methods

### Identification of the nodulation genes across legume species

To properly update the annotation of the functionally characterized nodulation genes, published nucleotidic sequences were used as a query for a BLAST search against the four different genome sequences [i.e., *M. truncatula* v4.0 (Young et al., 2011)*, G. max* Wm82.a2.v1 (Schmutz et al., 2010), *P. vulgaris* v1.0 (Schmutz et al., 2014), and *L. japonicus* v2.5 (Sato et al., 2008) available on the Phytozome v10.3 (http://phytozome.jgi.doe.gov/pz/portal.html) and Miyakogusa v3.0 (http://www.kazusa.or.jp/lotus/) websites]. Hits with an *e*-value <10^−12^ and a score > 100 were considered for further analysis.

### Syntenic analysis between nodulation genes

The Accelerating Comparative Genomic database [CoGe (https://genomevolution.org/coge/; Lyons and Freeling, 2008; Lyons et al., 2008)] was mined to characterize microsynteny relationships between legume genes. The most recent versions of the genome sequences available on the CoGe database were selected when highlighting microsynteny relationships (i.e.,*M. truncatula* v4.0*, G. max* v9.0, *P. vulgaris* v1.0 and *L. japonicus* v2.5). Only syntelog genes were used for further analysis. To better connect the v9.0 annotations of the soybean nodulation genes characterized by CoGe with the most recent release of the soybean genome (Wm82.a2.v1), we included both annotation systems in our analysis (Supplemental Table 1). The GEvo (genome evolution analysis) tool was applied to visualize the collinearity and/or rearrangement between syntenic regions.

### Transcriptomic analysis of soybean and *M. truncatula* nodulation genes

Gene expression atlases were mined to characterize the transcriptional patterns of the G. max, *M. truncatula, L. japonicas*, and *P. vulgaris* nodulation genes including in root hairs in response to rhizobia inoculation (Benedito et al., 2008; Libault et al., 2010b,c; Verdier et al., 2013; Breakspear et al., 2014; O'Rourke et al., 2014). The expression pattern of orthologous genes were compared based on the induction of gene expression in root hair cells upon rhizobia inoculation and based on their specific expression in nodules compared to other plant tissues.

### Gene density analysis

For each species, the annotation file (gff3) were collected from the Phytozome v10.3 (http://phytozome.jgi.doe.gov/pz/portal.html#) and the Miyakogusa v3.0 websites (http://www.kazusa.or.jp/lotus/).

The distribution of nodulation genes on the chromosomes was performed with a sliding window sizes of 10, 6, 5 Mb and a step of 1, 0.6, and 0.5 Mb for *G. max, P. vulgaris* and *L. japonicus*/*M. truncatula*, respectively. The gene density was normalized with a Z-score calculation [Z-score = (gene density-u)/st, u and st are the mean and the standard deviation of the gene density for each chromosome, respectively], and R package ggplot2 (http://ggplot2.org/) was used to draw plot. The sample function in R was used to produce random nodulation gene distribution. The Kolmogorov-Smirnov test was used to validate the specific distributions of the genes on the chromosomes.

## Results

### Syntenic relationships reveal the orthologous relationships between legume nodulation-related protein-coding genes

To perform the most comprehensive evolutionary analysis of the protein coding genes involved in nodulation, we mined the scientific literature allowing us to list 110 functionally characterized genes from *M. truncatula, L. japonicus, G. max*, and *P. vulgaris* (Supplemental Table 1). To date, most protein coding genes involved in nodulation genes have been characterized in the model legumes *G. max* and *M. truncatula* notably upon the development of the *Tnt1* retrotransposon insertion mutant population (Tadege et al., 2008; Pislariu et al., 2012; Cui et al., 2013). This observation supports the need to identify orthologous genes between legume species to facilitate the transfer of the scientific knowledge.

To update the annotation of these 110 genes, we first blasted their published nucleotidic sequences against the most recent release of the legume genome sequences [i.e., Phytozome v10.3 (http://phytozome.jgi.doe.gov/pz/portal.html) for *M. truncatula* (Mt4.0v1)*, G. max* (Wm82.a2.v1), and *P. vulgaris* (v1.0); 2- Miyakogusa v3.0 (http://www.kazusa.or.jp/lotus/) for *L. japonicus* (v2.5); *e*-value <10^−12^ and score > 100; Supplemental Table 1].

**Figure 1:**
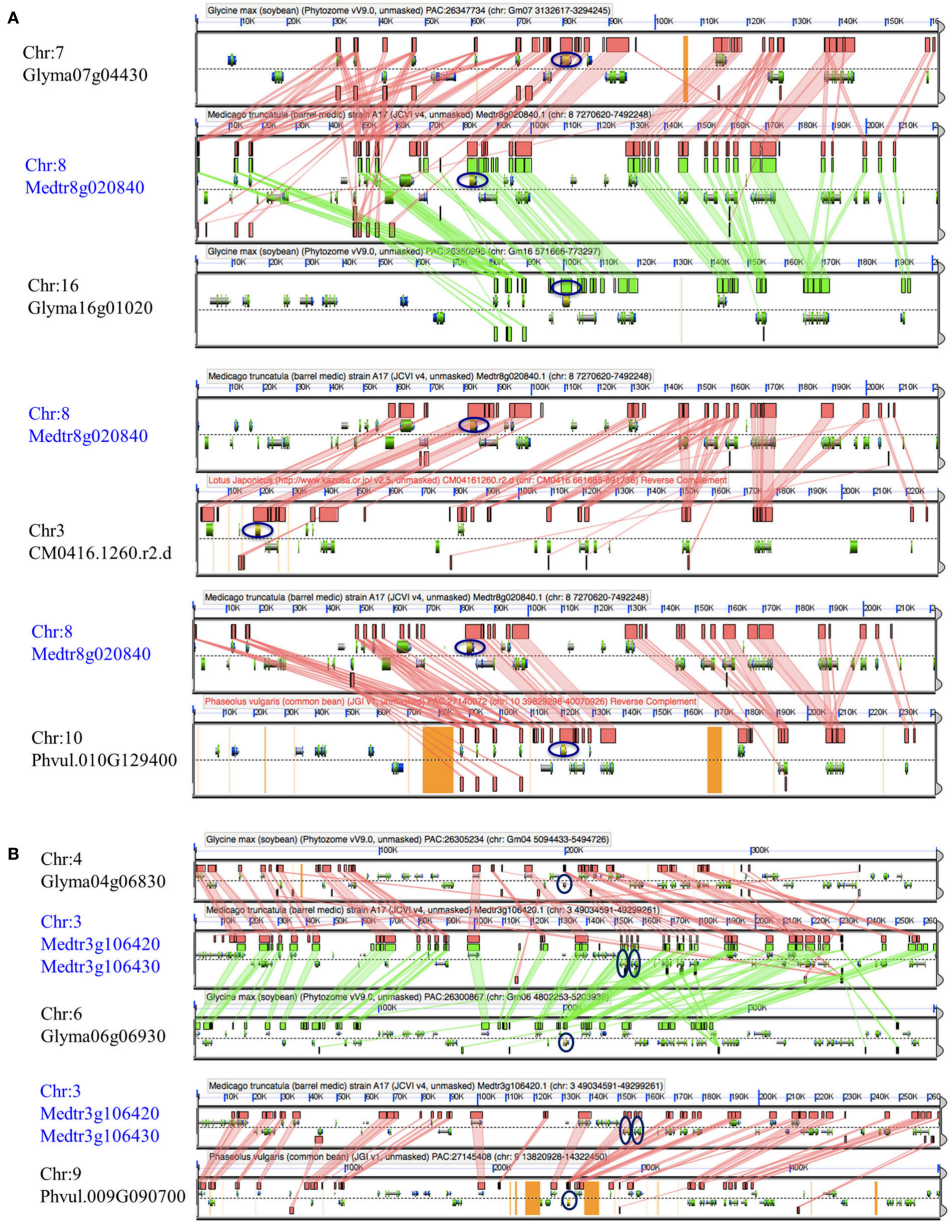
**Syntenic relationships between Mt***NSP1*** (A) and Mt***FLOT2/4*** (B) loci and chromosome regions from ***Glycine max***, ***Medicago truncatula***, ***Lotus japonicas***, and ***Phaseolus vulgaris*****. Each panel is a visualization of chromosome region showing the gene models on positive and negative strands. Green and red blocks in each panel highlight microsyntenic regions between legumes based on gene function and orientation. Genes highlighted in blue (e.g., *Medtr8g020840* and *Medtr3g106420/Medtr3g106430* for *NSP1* and *FLOT2/4* genes, respectively) were used as query when performing microsynteny analysis against the four legume species. The *NSP1* and *FLOT2/4* orthologs based on their microsyntenic relationships are highlighted with blue circles.

To identify orthologous and paralogous genes in and between *L. japonicus, M. truncatula, G. max*, and *P. vulgaris*, we examined syntenic relationships between corresponding genomic regions based on gene content, order, and orientation. To perform this analysis, we took advantage of the release of the sequence of various legume genomes and the development of comparative genomic resources such as CoGe (Lyons and Freeling, 2008; Lyons et al., 2008). Upon our evolutionary analysis, we repetitively observed strong syntenic relationships between genes from the four legume species including a large number of soybean paralogs, a consequence of the most recent whole genome duplication (WGD) of the soybean genome (Schmutz et al., 2010; Figure 1; Supplemental Table 1 and Supplemental Figure 1 for access to the entire datasets). Together, this comparative genomic analysis led to the characterization of 191, 92, 65 and 91 soybean, *M. truncatula, L. japonicas*, and common bean genes orthologous and paralogous to functionally described nodulation genes, respectively. These genes belong to 81 orthologous/paralogous groups (Supplemental Table 1).

### Comparative transcriptomic analysis reveals conservation and neo-/sub-functionalization between *M. truncatula, G. max, L. japonicus and P. vulgaris* nodulation genes

While microsynteny relationships clearly revealed the orthology existing between nodulation genes from different species, they are not sufficient to conclude about the conservation of their function. To provide a first insight into the conservation and divergence of the function between orthologous genes, we mined transcriptomic databases and integrated them into our comparative genomic analysis. Specifically, we took advantage of the release of the *M. truncatula* and soybean root hair transcriptomes and their perturbation in response to rhizobia inoculation (Libault et al., 2010b; Breakspear et al., 2014) as well as the access to the *M. truncatula, G. max, L. japonicas*, and *P. vulgaris* transcriptome atlases (Benedito et al., 2008; Libault et al., 2010c; Verdier et al., 2013; O'Rourke et al., 2014). In addition, we included in our analysis more focused transcriptomic studies on the nodulation process in the model plant *M. truncatula* (Roux et al., 2014; Larrainzar et al., 2015).

**Figure 2:**
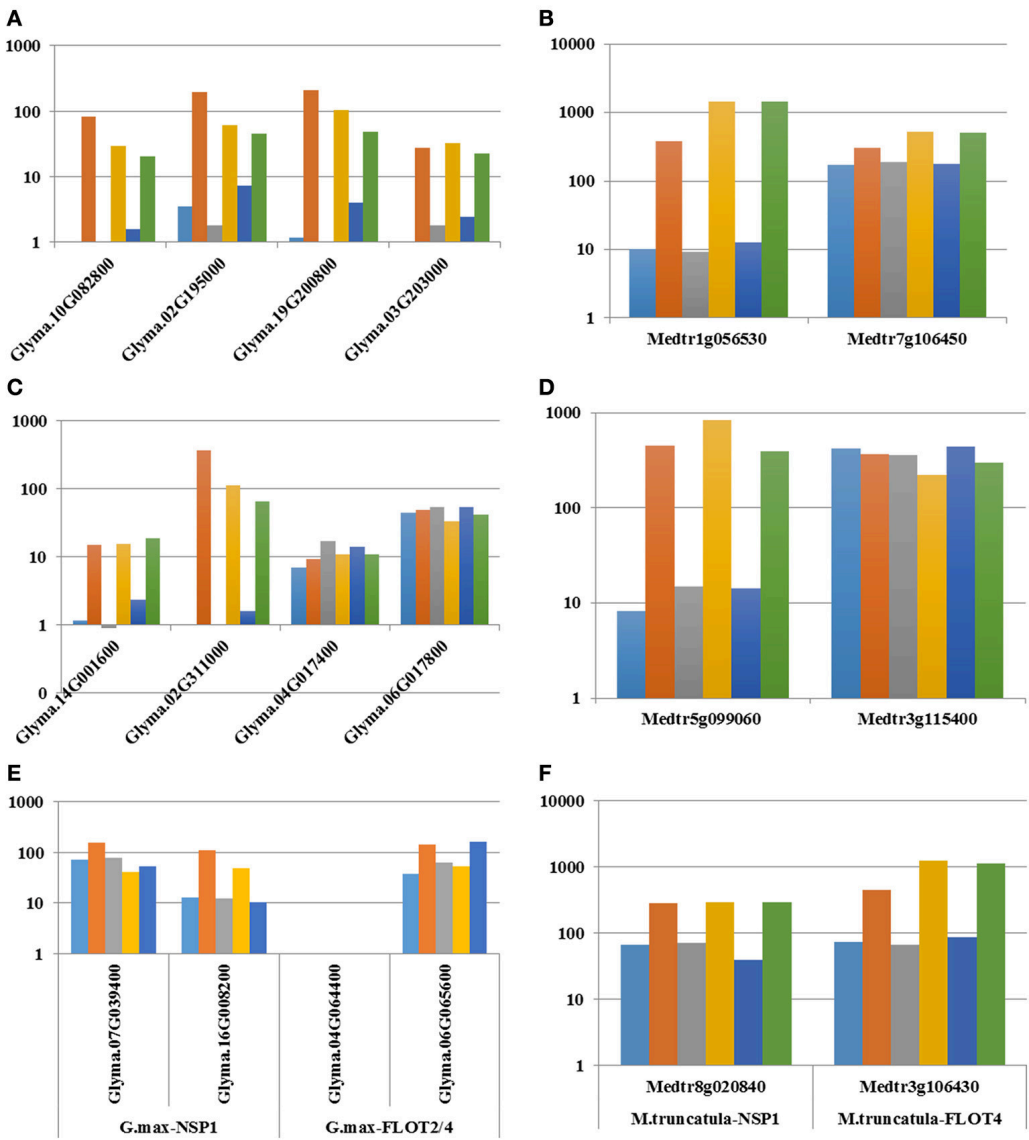
**Relative expression levels of ***NF-YA1/2*** (A, B), ***NIN*** (C, D), ***NSP1***, and ***FLOT2/4*** (E, F) ***M. truncatula*** genes (B, D, F), and their ***G. max*** orthologs (A, C, E) in inoculated and mock-inoculated root hair cells**. Gene IDs are highlighted on the x-axis. The relative expression levels of the genes (log10 scale) are indicated on the y-axis. Gene expression datasets were mined from the *G. max* and *M. truncatula* root hair transcriptomic datasets (Libault et al., 2010b; Breakspear et al., 2014). For *G. max*: blue, 12H UN; orange, 12H IN; gray, 24H UN; yellow, 24H IN; dark blue, 48H UN; and green, 48H IN; For *M. truncatula*: blue, 1D UN; orange, 1D IN; gray, 2D UN; yellow, 2D IN; dark blue, 3D UN; and green, 3D IN.

**Figure 3:**
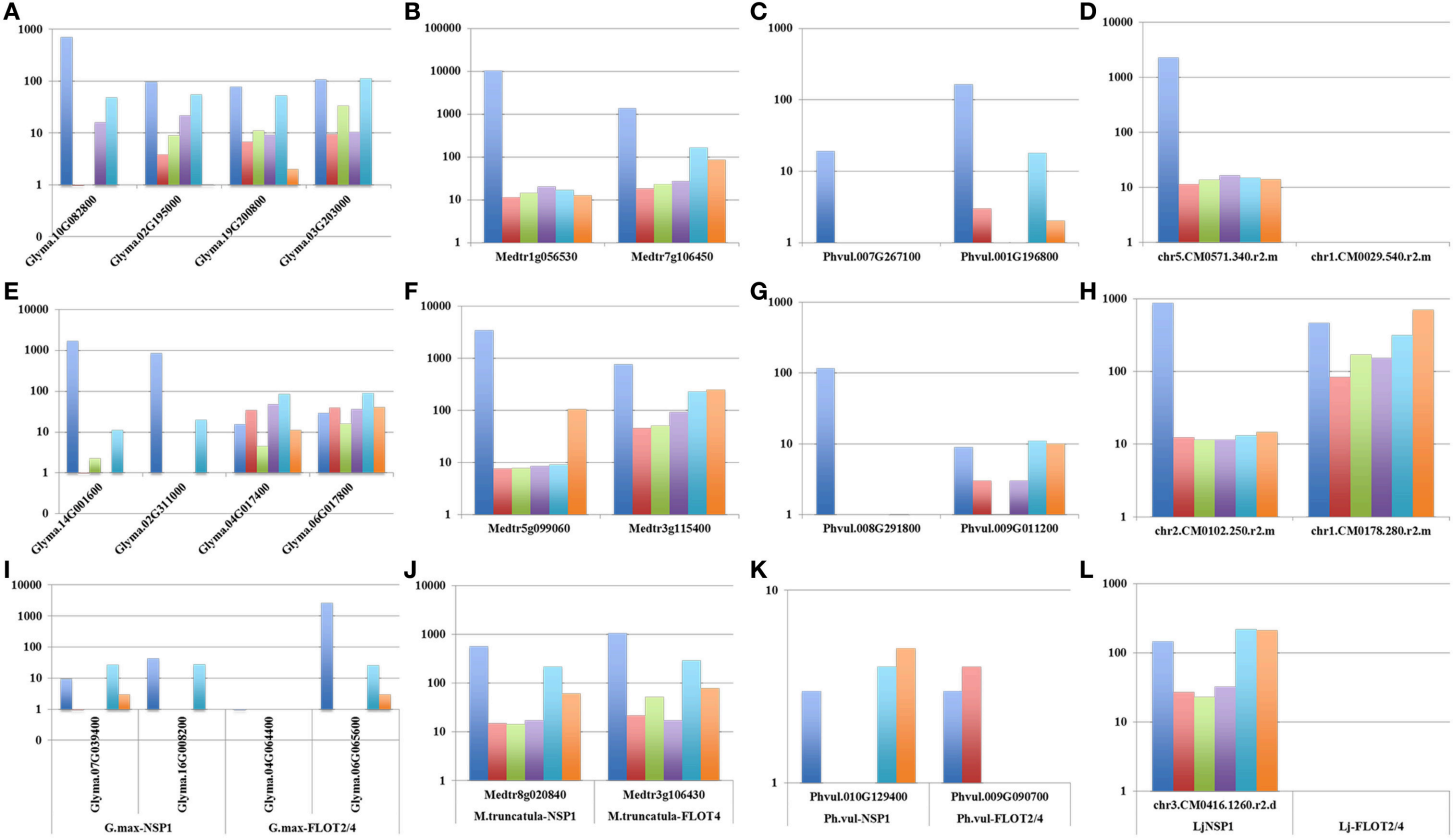
**Relative expression levels of ***NF-YA1/2*** (A–D), ***NIN*** (E–H), ***NSP1*** and ***FLOT2/4*** (I–L) of ***M. truncatula*** genes (B, F, J), and their ***G. max*** orthologs (A, E, I), ***P. vulgaris*** orthologs (C, G, K) and ***L. japonicus*** (D, H, L) in various plant organs (i.e., blue, nodule; orange, flower; gray, pod; yellow, leaf; dark blue, root; and green, root tip)**. Gene IDs are highlighted on the x-axis. The relative expression levels of the genes (log10 scale) are indicated on the y-axis. Gene expression datasets were mined from the *G. max, M. truncatula, P. vulgaris*, and *L. japonicus* atlases datasets (Benedito et al., 2008; Libault et al., 2010c (http://mtgea.noble.org/v3/); Verdier et al., 2013; O'Rourke et al., 2014).

To identify the entire set of legume genes transcriptionally induced during nodulation, we independently analyzed their expression pattern during both the early (i.e., root hair response to rhizobia inoculation) and late events of the nodulation process (i.e., nodule specific expression compared to other plant organs). A total of 18 and 19 *M. truncatula* genes were induced in root hair cells in response to wild-type *Sinorhizobium meliloti* inoculation or were preferentially expressed in nodules compared to other plant tissues (fold-change > 2; Supplemental Table 2), respectively. Among those genes, eleven were both induced in root hair cells in response to *S. meliloti* and preferentially expressed in nodules including Mt*NF-YA1/2* (Figures 2B, 3B), Mt*NIN* (Figures 2D, 3F), Mt*FLOT2/4*, and Mt*NSP1* (Figures 2F, 3J). To provide a more complete understanding of the *M. truncatula* genes transcriptionally induced during nodulation, we mined recently published *M. truncatula* RNA-seq data sets (Roux et al., 2014; Larrainzar et al., 2015). We identified a total of 30 and 15 *M. truncatula* genes differentially expressed in specific zones of the *M. truncatula* nodule (i.e., Roux et al., 2014) and during the early stages of nodulation in root tissue (i.e., from 30 min to 2 days after *S. meliloti* inoculation; Larrainzar et al., 2015), respectively, (Supplemental Table 2). Analyzing the soybean transcriptome, the expression of 47 genes was induced in root hair cells in response to rhizobia inoculation while 38 soybean genes were preferentially expressed in nodules vs. other soybean tissues. Among those, 20 were both up-regulated in root hairs in response to *B. japonicum* and preferentially expressed in nodules such as Gm*NF-YA1/2* (Figures 2A, 3A), Gm*NIN* (Figures 2C, 3E), and Gm*FLOT2/4* (Figures 2E, 3I). Mining the common bean and *L. japonicus* transcriptome atlases, we also characterized 19 and 16 genes preferentially expressed in common bean and *L. japonicus* nodules, respectively, such as Pv*NF-YA1/2* (Figure 3C), Pv*NIN* (Figure 3G), Lj*NF-YA1/2* (Figure 3D), Lj*NIN* (Figure 3H). Unexpectedly, Pv*NSP1*, PvFLOT2*/4*, and Lj*NSP1* are not preferentially expressed in common bean and *L. japonicus* nodules (Figures 3D,H,K,L).

**Figure 4:**
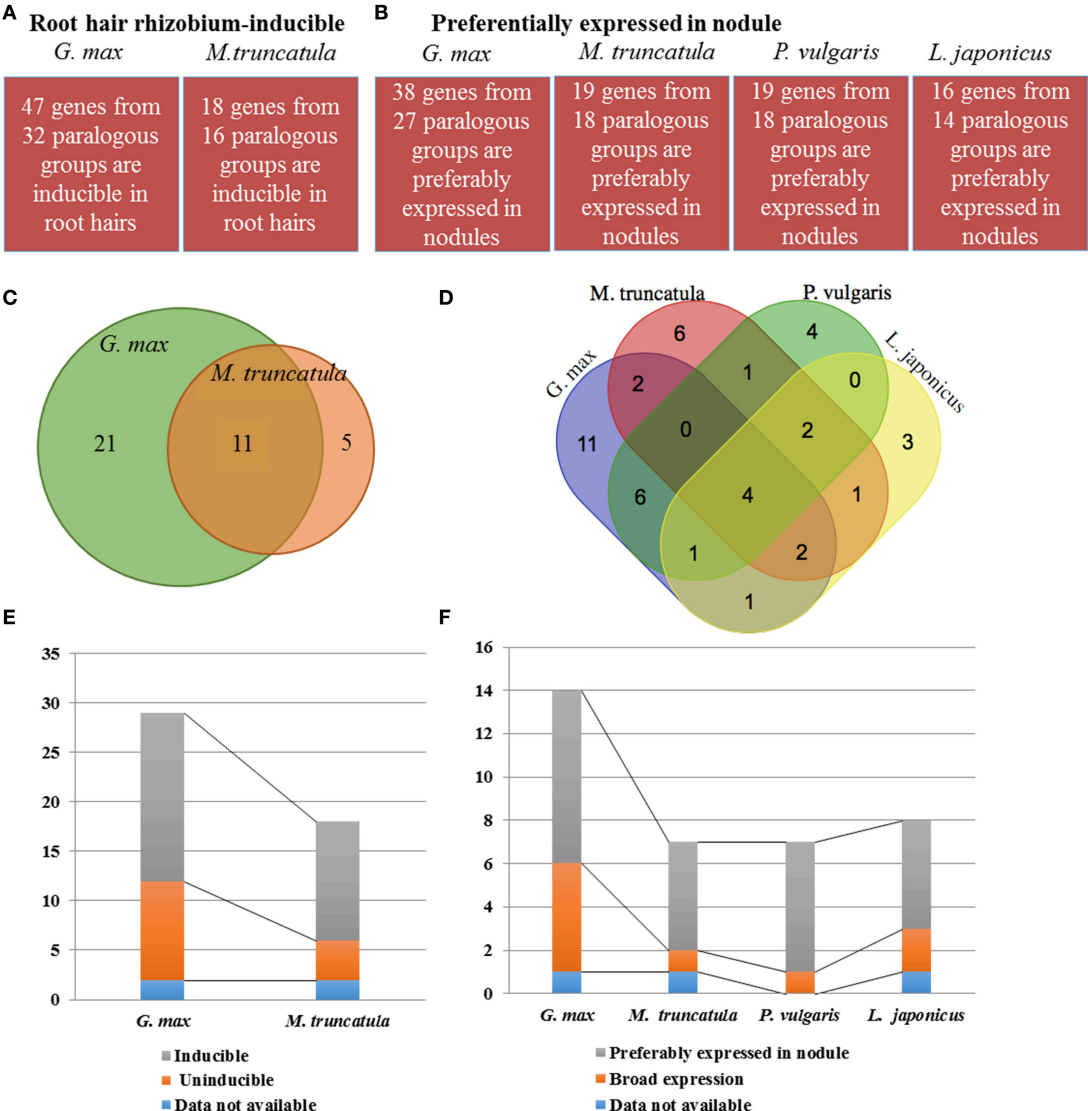
**Comparison of the expression profiles between ***G. max***, ***M. truncatula***, ***P. vulgaris***, and ***L. japonicus*** orthologous genes and groups during the early (i.e., root hair response to rhizobia inoculation; A, C, E) and late stages of the nodulation process (i.e., preferential expression in mature nodules; B, D, F)**. **(A)** and **(B)**, numbers of *G. max* and *M. truncatula* genes and orthologous groups induced in root hairs upon rhizobia inoculation **(A)** and *G. max, M. truncatula, P. vulgaris*, and *L. japonicus* genes preferentially expressed in nodules (**B**; fold-change > 2; Supplemental Table 3 for details). **(C)** and **(D)**: Venn diagrams showing the overlaps between *G. max* and *M. truncatula* root hair inducible **(C)** and overlaps between *G. max, M. truncatula, P. vulgaris*, and *L. japonicus* nodule-specific **(D)** orthologous groups; **(E)** and **(F)**: Distribution of the number of *G. max* and *M. truncatula* genes which belong to the 11 root hair-inducible **(E)** and the number of *G. max, M. truncatula, P. vulgaris*, and *L. japonicus* genes which belong to four nodule preferential **(F)** orthologous groups, respectively, according to their expression patterns.

To better evaluate the impact of soybean WGDs on the population of genes controlling nodulation, we classified and compared the 47 and 18 soybean and *M. truncatula* genes induced in root hair cells in response to rhizobia inoculation based on their paralogous relationships. A total of 32 and 16 paralogous groups were identified for each plant species, respectively, (Figure 4A; Supplemental Table 1, Supplemental Table 3). Performing a similar analysis on the 38, 19, 19 and 16 soybean, *M. truncatula*, common bean and *L. japonicus* genes preferentially expressed in nodules, we characterized 27, 18 18 and 14 groups of paralogous genes, respectively, (Figure 4B, Supplemental Table 3). These results suggest that the large number of differentially expressed soybean genes during nodulation is not just the consequence of the WGDs.

Upon WGDs, paralogs can share the same expression profiles leading to functional redundancy [e.g., induction of the expression of the four soybean *NF-YA1/2* paralogs in root hair cells in response to *B. japonicum* inoculation (Figure 2A)], or could be a source for sub- and neo-functionalization [(e.g., Gm*NIN*s orthologs can be divided into two groups: *Glyma.14G001600* and *Glyma.02G311000*, which are induced during nodulation and *Glyma.04G017400* and *Glyma.06G017800*, characterized by their more constitutive expression Figures 2C, 3E)]. To further explore the consequences of WGDs on the transcriptional regulation of paralogous genes, we compared the expression of soybean and *M. truncatula* genes that belong to paralogous groups that are induced during nodulation.

We identified 11 overlapping root hair inducible groups between the two plant species (34 and 69% of the *G. max* and *M. truncatula* root hair-inducible groups, respectively; Figure 4C, Supplemental Table 3). Twenty-nine and 18 soybean and *M. truncatula* genes are represented in these 11 groups, respectively, including the *NOD100, NMNa, RIP1, MtPUB1, NODULIN-26a*, NIN, *FLOT2/4, NSP1, MtNramp1, NF-YA1/2*, and *ERN1/2* genes (Supplemental Table 3). Performing a similar analysis on the 27, 18, 18 and 14 *G. max, M. truncatula, P. vulgaris*, and *L. japonicus* groups preferentially expressed in nodules, we characterized four overlapping orthologous groups (11, 22, 22, and 29% of the *G. max, M. truncatula, P. vulgaris*, and *L. japonicus* groups preferentially expressed in nodules, respectively; Figure 4D). These four groups are composed of 14, 7, 7, and 8 *G. max, M. truncatula, P. vulgaris*, and *L. japonicus* genes, respectively, such as *ENOD20, NIN, NIN2*, and *NF-YA1/2* genes (Supplemental Table 3).

Analyzing the expression patterns of these genes, we only observed a slight increase in the number of soybean genes differentially expressed during the early (Figure 4E; gray bar) and late events of the nodulation process compared to *M. truncatula, P. vulgaris*, and *L. japonicus* genes (Figure 4F, gray bar). Oppositely, many soybean paralogs are not transcriptionally responsive to rhizobium, but are characterized by their broad expression patterns (Figures 4E,F, orange bars). These results suggest that upon soybean WGDs, a subset of the paralogous genes were transcriptionally restricted to their role during nodulation based on the conservation of their induction in response to rhizobia while other paralogs gained new function after alteration of their expression patterns.

### Legume genes specialized in the nodulation processes are forming gene modules on legume chromosomes

**Figure 5:**
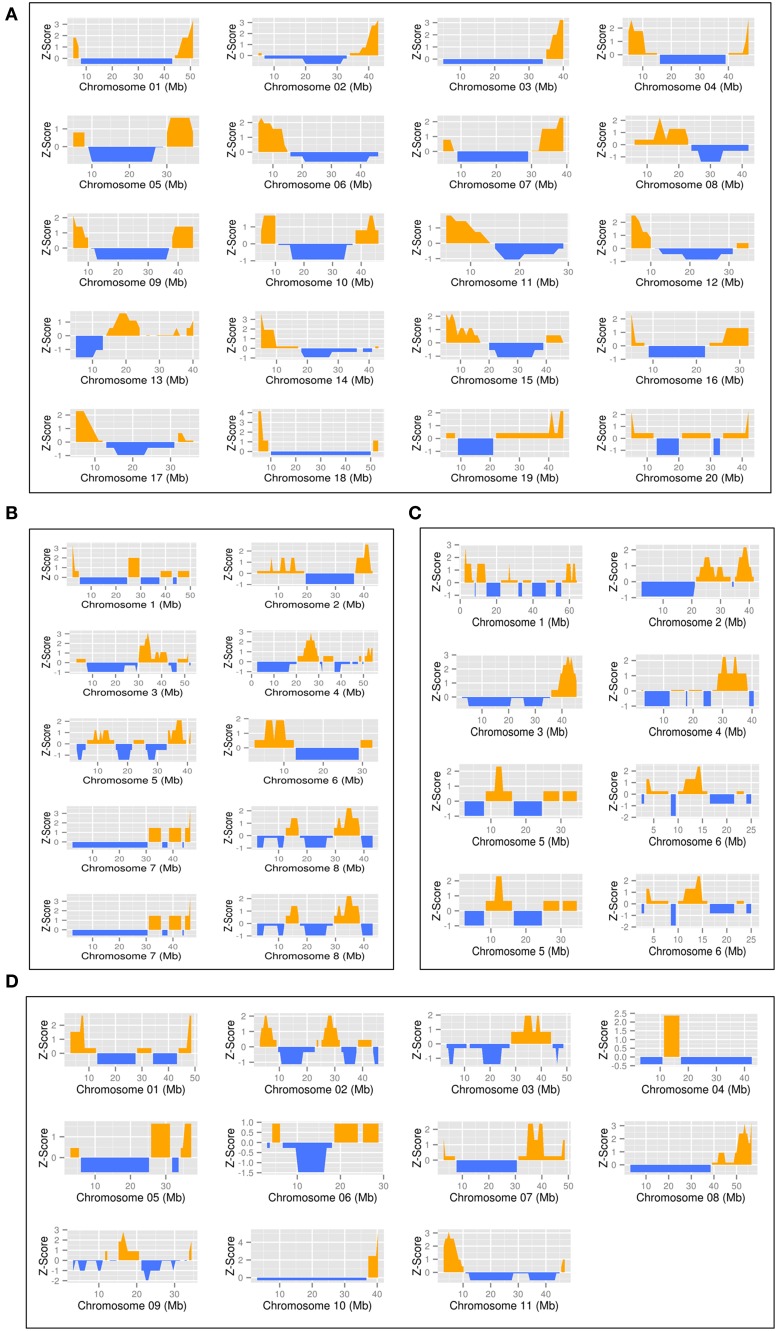
**Representation of the density of the legume nodulation genes along each legume chromosome**. Gene density is represented by a z-score in a 10 Mb sliding window (step 1 Mb) along each chromosomes for each legume genome. Positive values are in orange, negative value in blue. The position on the chromosomes are indicated in Mb. The genomes represented are *Glycine max* **(A)**, *Medicago truncatula* **(B)**, *Lotus japonicus* **(C)**, and *Phaseolus vulgaris* **(D)**.

Previous studies revealed the correlation existing between gene function and the organization of the euchromatin. Specifically, gene modules on chromosomes are characterized by the presence of genes involved in the same biological function and sharing similar expression patterns (Williams and Bowles, 2004; Zhan et al., 2006; Pingault et al., 2015). Hypothesizing that nodulation genes are also located in modules along the legume chromosomes, we analyzed their chromosomal distribution and density. Accordingly, we mapped the annotated legume genes controlling nodulation (Supplemental Table 1) on the 20, 8, 6, and 11 chromosomes of *G. max, M. truncatula, L. japonicas*, and *P. vulgaris*, respectively. To take in consideration the differences in the size of the genomes of the four legume plants, we analyzed the distribution of nodulation genes on the chromosomes generating window sizes of 10, 6, 5 Mb, and steps of 1, 0.6, and 0.5 Mb for *G. max, P.vulgaris* and *L. japonicus*/*M. truncatula*, respectively. To highlight the significant increase of the density of nodulation genes on chromosomes as reflected by their Z-score, we also normalized these results based on a random distribution of the nodulation genes (Figure 5). A significant enrichment in nodulation genes was repetitively observed on the *M. truncatula, L. japonicas*, and *P. vulgaris* chromosomes and on 12 out of the 20 soybean chromosomes (Kolmogorov-Smirnov test; *p*-value <0.05; Supplemental Table 4). Together, these results confirmed the clustering of nodulation genes in functional modules on the chromosomes. The fact that these modules are conserved between model legumes suggests their essential roles in controlling the nodulation process.

## Discussion

### Applying comparative genomic and transcriptomic analyses to reveal the conservation and divergence between nodulation orthologous/paralogous genes

Functional genomic studies led to the characterization of 110 genes controlling the early and late events of the nodulation process. As described by Chen et al. (2015), a core set of *M. truncatula* genes such as *ERN1/2, NSP2*, and several *NF-Y* genes are playing a central role in plant cell infection by rhizobia, both early (i.e., root hair cell infection) and late (i.e., nodule cell infection) during the nodulation process (Chen et al., 2015). In this study, our analyses have been conducted in four different legume models having the objective to better transfer scientific knowledge between species. Accordingly, we took advantage of the genomic information now available to perform a global and comprehensive analysis of the evolution of nodulation genes based on: 1- their orthology and paralogy upon demonstration of their microsyntenic relationships and; 2- the conservation and divergence of their transcriptomic patterns.

Applying various bioinformatics tools available on the CoGe platform (https://genomevolution.org/coge/) such as Synfind and GEvo, we repetitively analyzed the evolutionary relationship between nodulation genes across four model legume plants. Upon the availability of functional genomic datasets, we were able to confirm the orthologous relationships existing between genes known to control nodulation across different species. For instance, using the soybean NF receptor gene Gm*NFR1a* (Indrasumunar et al., 2011) as query, we clearly highlighted its orthology with the *M. truncatula* Mt*LYK3/HCL* (Limpens et al., 2003) and the *L. japonicus* Lj*NFR1* genes (Radutoiu et al., 2003). To further support our analysis, we revealed the orthology existing between 65 functionally characterized genes across different species [e.g., *SYMRK / NORK, SUNN/NARK/HAR1, RDN2, POLLUX/DMI1, NSP2, NSP1, NIN, NFR/NFP, NFR1/LYK3/HCL, MtENOD20/ENOD55-2*. *ENOD16/ENOD55-1* and *CCaMK/DMI3* genes (Supplemental Figure 1)].

While the conservation of the function between orthologous genes is often assumed, biological evidences to validate these assumptions are limited. Transcriptomes provide a first insight into the conservation of the function of orthologous and paralogous genes based on the conservation of a similar expression pattern. For instance, previous studies focusing on the fate of soybean paralogs, products of the successive duplications of the soybean genome, concluded about the differential expression of 50% of the paralogs leading to their sub-functionalization (Roulin et al., 2013). Taking advantage of the release of legume transcriptome atlases as well as the analysis of the transcriptomic response of the root hair cells to rhizobia inoculation, we compared the nodulation related protein coding genes' expression patterns between soybean, *M. truncatula*, common bean and *L. japonicus* orthologous genes. These analyses were also conducted under the context of paralogy. Our study clearly revealed the conservation of the transcriptomic patterns between *M. truncatula, G. max, L. japonicas*, and *P. vulgaris* orthologous genes during the nodulation process supporting the conservation of the biological function of at least one paralog. Interestingly, a significant number of soybean paralogs can display very distinctive expression patterns suggesting a gain of function or a sub-functionalization (e.g., soybean *NIN* genes; Figures 2, 3, 4). Differential epigenome and the evolution of the promoter regions of paralogous genes (i.e., presence and absence of cis- and trans-regulatory elements) should be carefully investigated to reveal the evolutionary mechanisms controlling these transcriptomic changes.

Plant genes acting together in the same biological process are physically closely located on the chromosomes (Williams and Bowles, 2004; Zhan et al., 2006; Pingault et al., 2015). Taking advantage of the identification of a large number of nodulation genes from four different species (i.e., 191, 92, 65 and 91 *G. max, M. truncatula, L. japonicus, P. vulgaris* genes, respectively), we analyzed their distribution along the 20, 8, 6, and 11 chromosomes of each species, respectively. We observed that the legumes genes controlling the nodulation process are characterized by their high density on the chromosomes rather than being randomly located on the chromosomes. This close relationship might be beneficial to their co-expression during nodulation. The relationships existing between chromosome territories, position of gene modules on the chromosome, their epigenomic context and their transcriptional activities should be investigated.

## Author contributions

ML designed research. MN characterized and updated the annotation of the legume nodulation genes. ZQ performed the syntenic and transcriptomic analysis between legume genes. LP analyzed the distribution of the nodulation genes on the legume chromosomes. ML wrote the article.

## Funding

This work was funded by a grant from the National Science Foundation-Plant Genome Research Program (#IOS-1339194) and by the National Science Foundation-CAREER program (#IOS-1453613).

### Conflict of interest statement

The authors declare that the research was conducted in the absence of any commercial or financial relationships that could be construed as a potential conflict of interest.
