# Supplementary material for: Comprehensive Comparative Genomic and Transcriptomic Analyses of the Legume Genes Controlling the Nodulation Process
Source: Front Plant Sci. 2016 Jan 29;7:34. doi: 10.3389/fpls.2016.00034 (PMC4732000; doi:10.3389/fpls.2016.00034)

Supplemental Figure 1. Syntenic relationships between *Glycine max*, *Medicago truncatula*, *Lotus japonicus* and *Phaseolus vulgaris* to reveal orthology and paralogy between nodulation genes. Each panel highlights the syntenic relationship between orthologous/paralogous nodulation genes based on the conservation of gene function, order and direction on the positive and negative strands of the genomic DNA. These syntenic relationship were generated using the Accelerating Comparative Genomic database [CoGe (https://genomevolution.org/coge/) (Lyons and Freeling, 2008;Lyons et al., 2008)].

ENOD8 orthologous genes in genomic regions of four legume plants

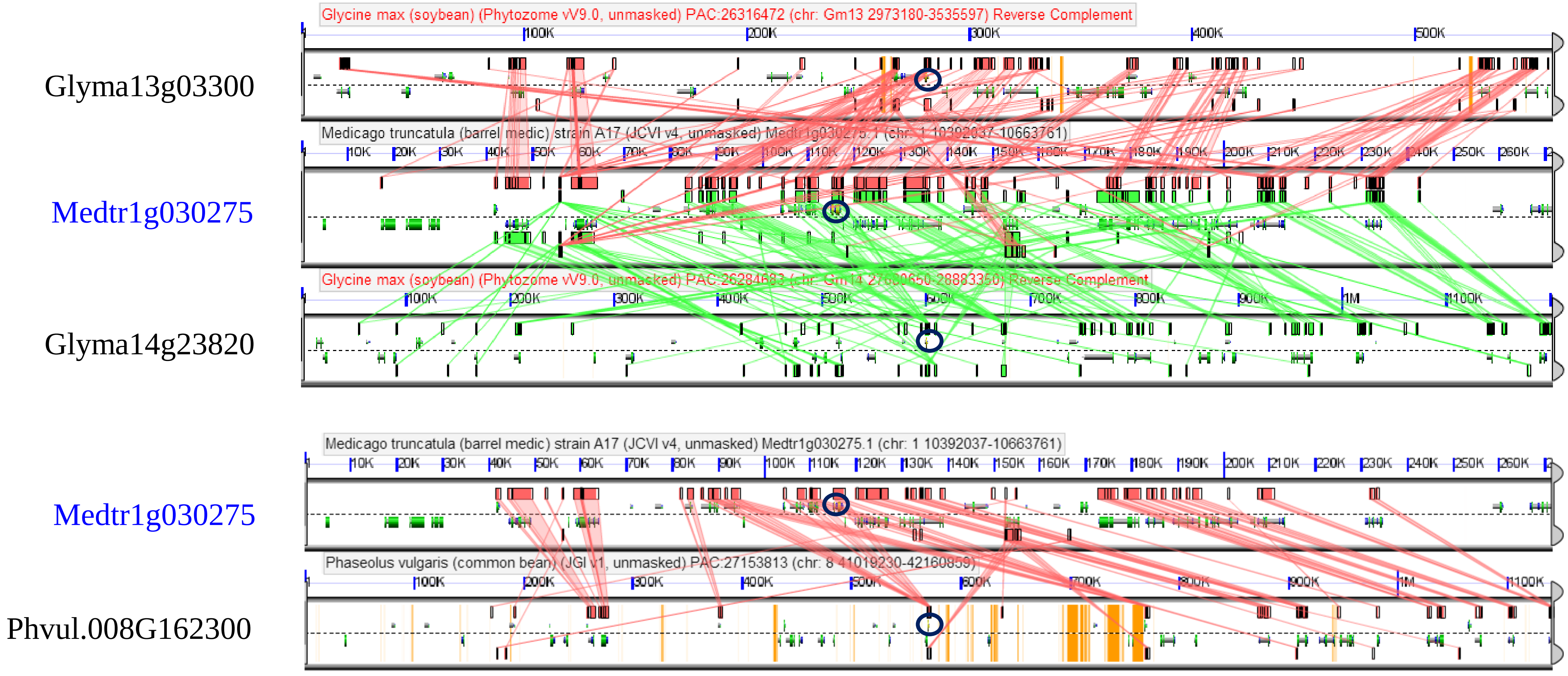

ENOD16 orthologous genes in genomic regions of four legume plants

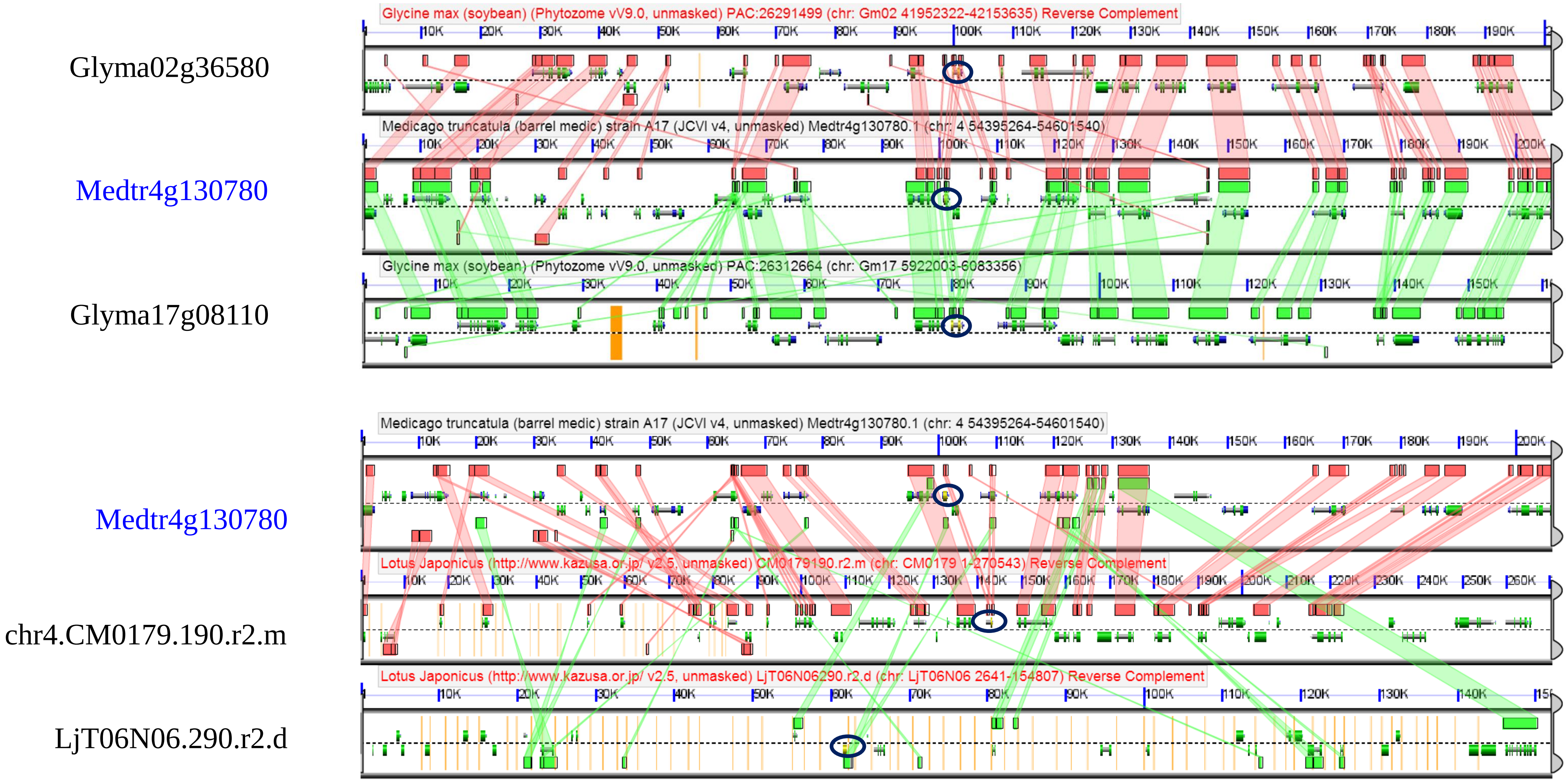

ENOD20 orthologous genes in genomic regions of four legume plants

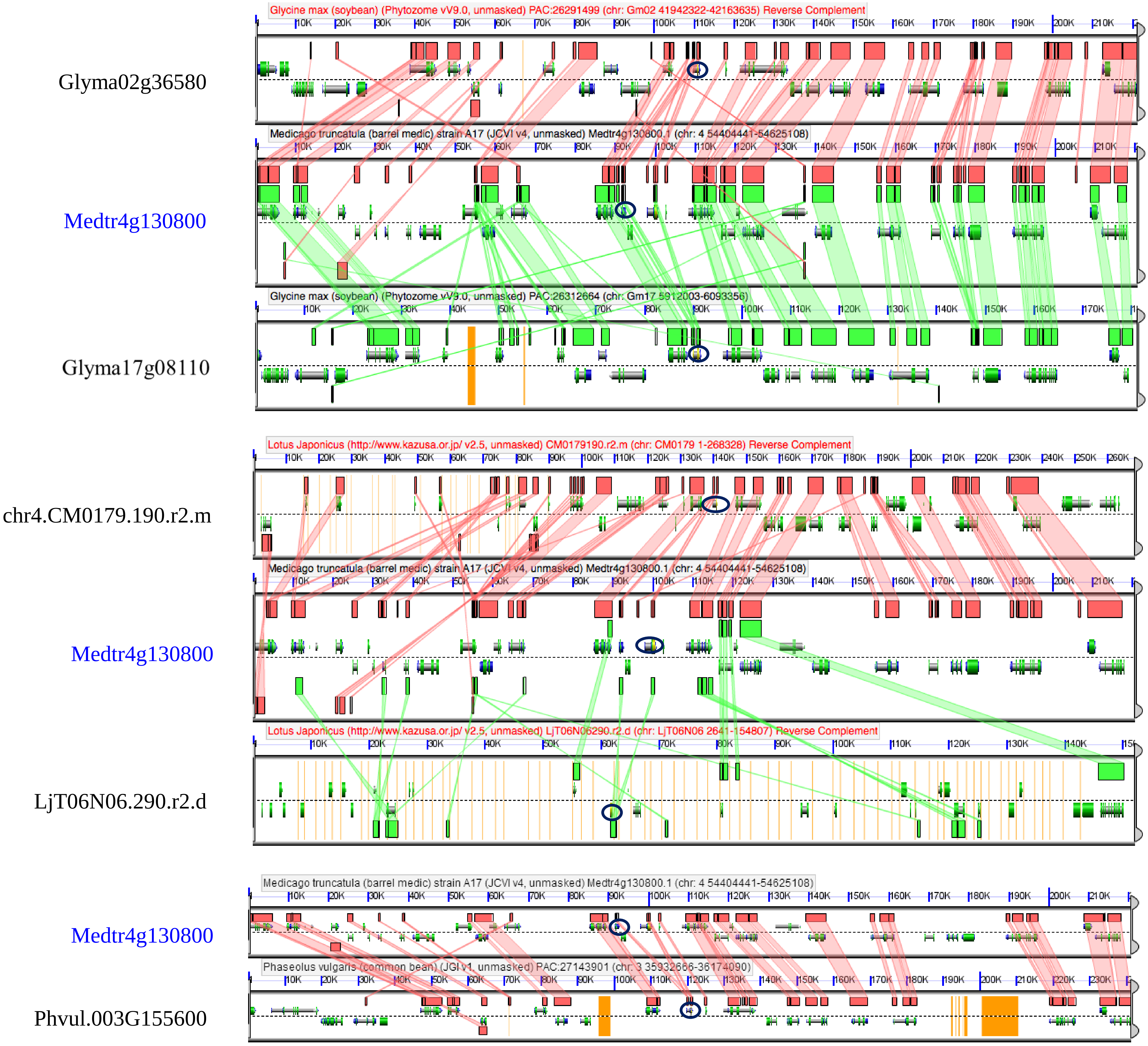

NOD 26 orthologous genes in genomic regions of four legume plants

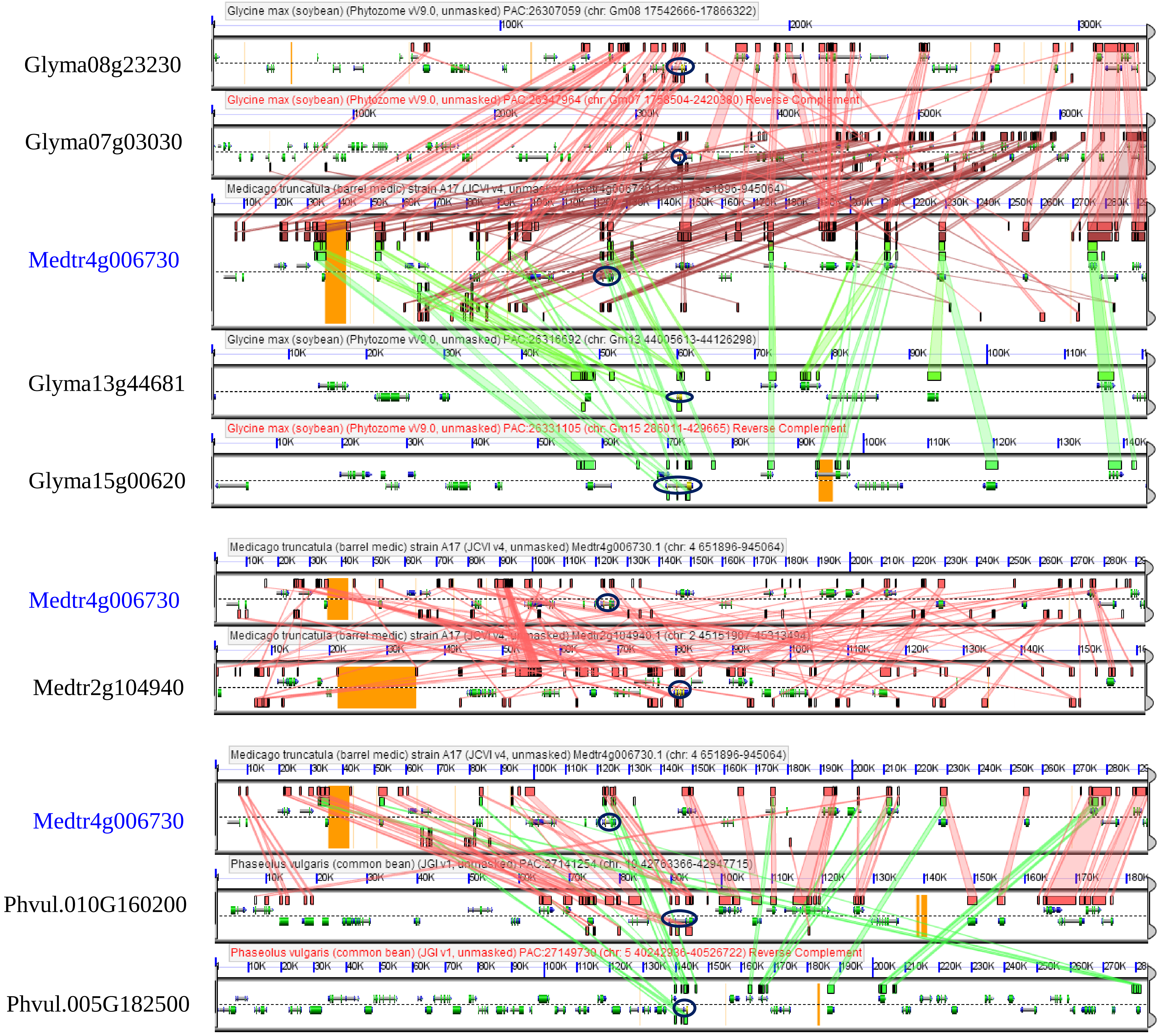

ENOD 40 orthologous genes in genomic regions of four legume plants

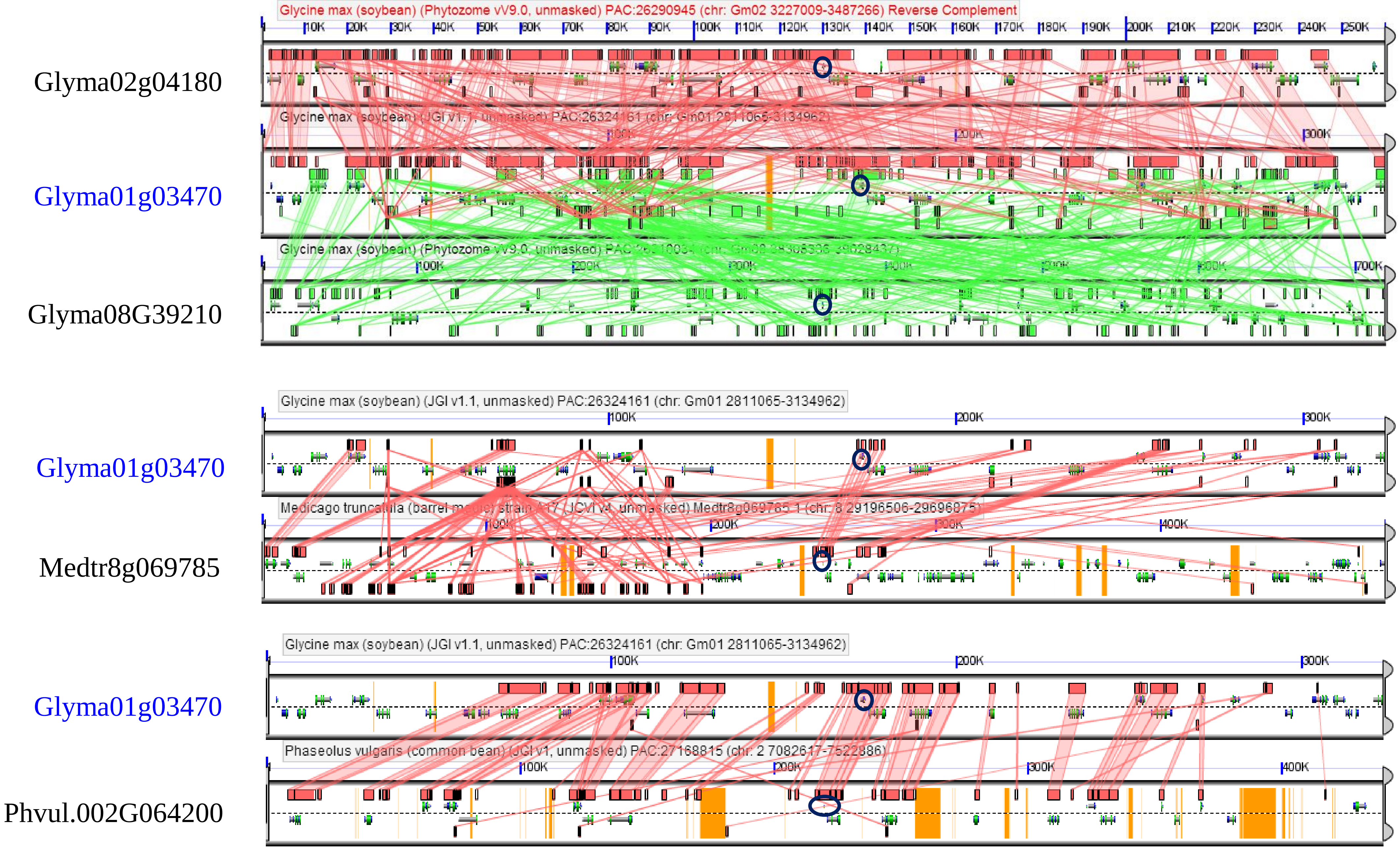

NOD35 orthologous genes in genomic regions of four legume plants

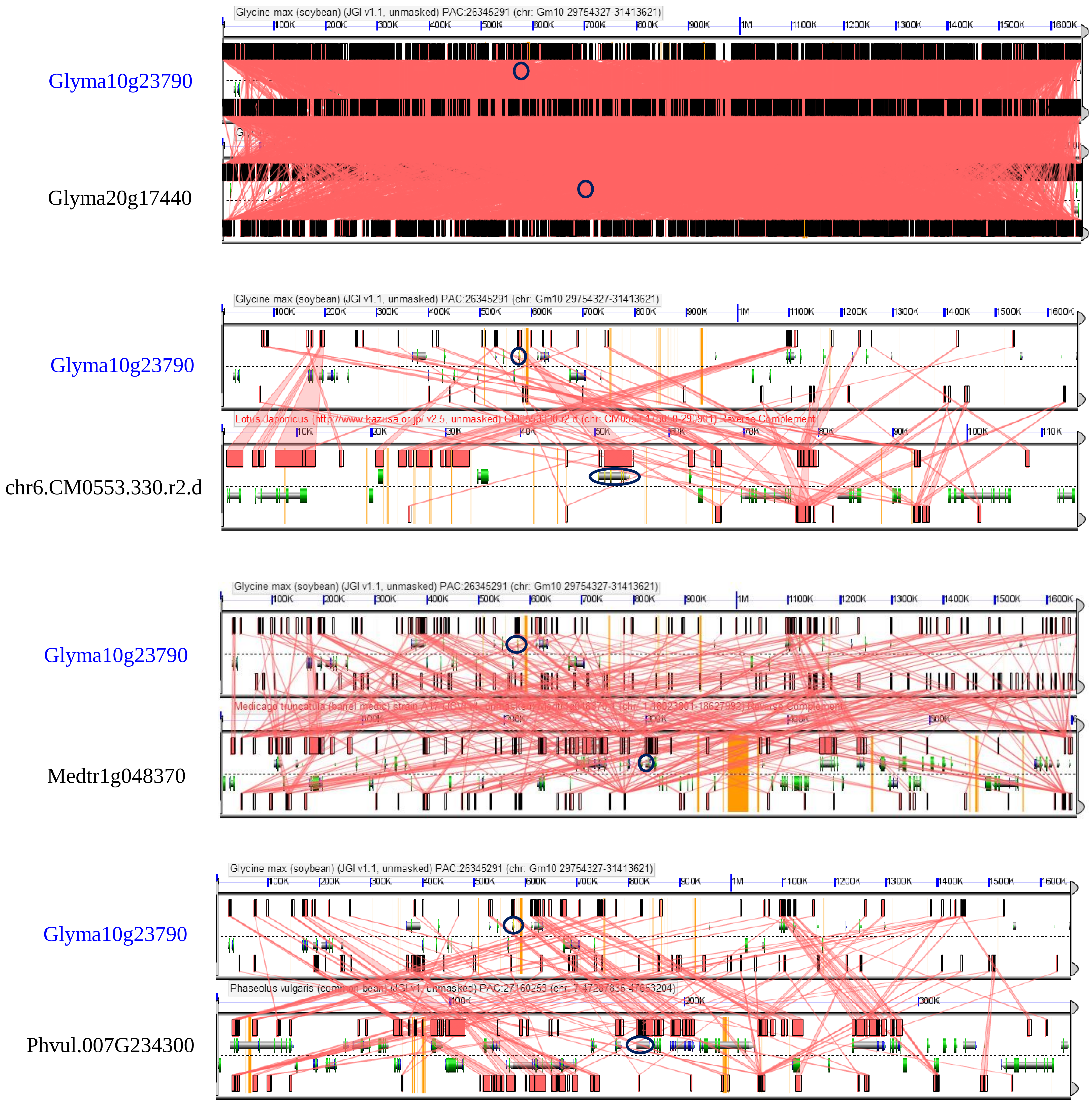

NOD100 orthologous genes in genomic regions of four legume plants

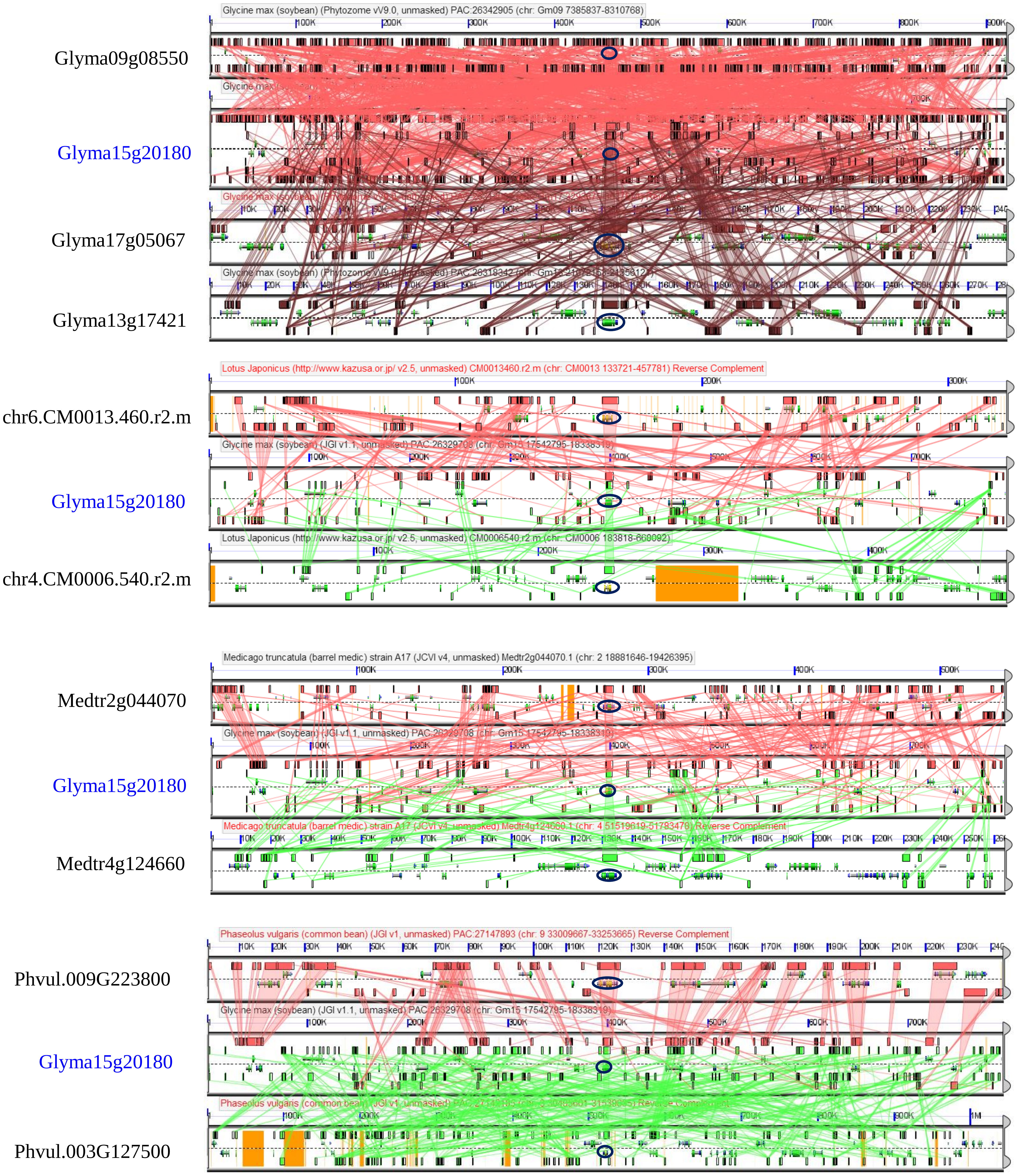

EFD orthologous genes in genomic regions of four legume plants

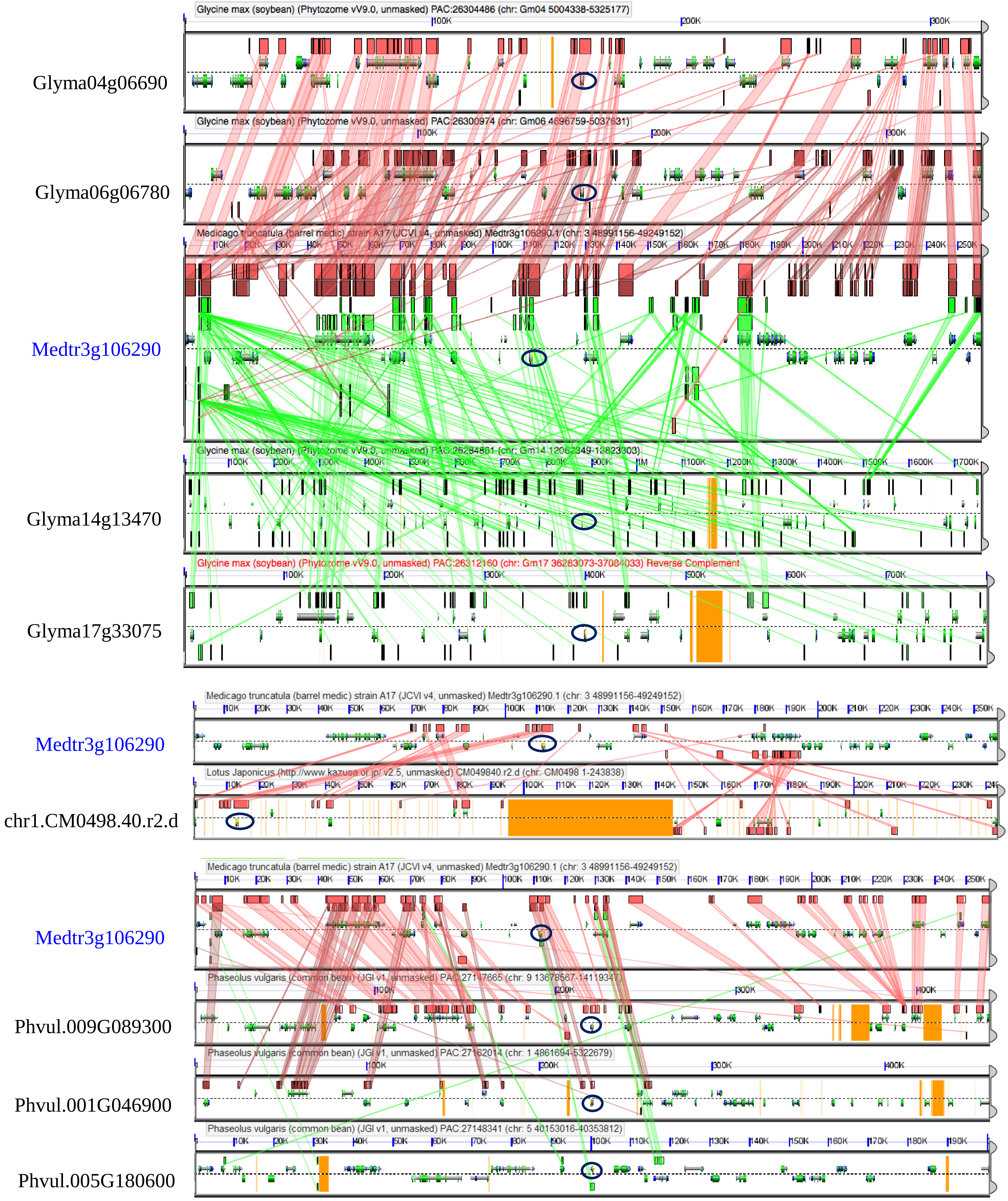

NF-YA1/2 orthologous genes in genomic regions of four legume plants

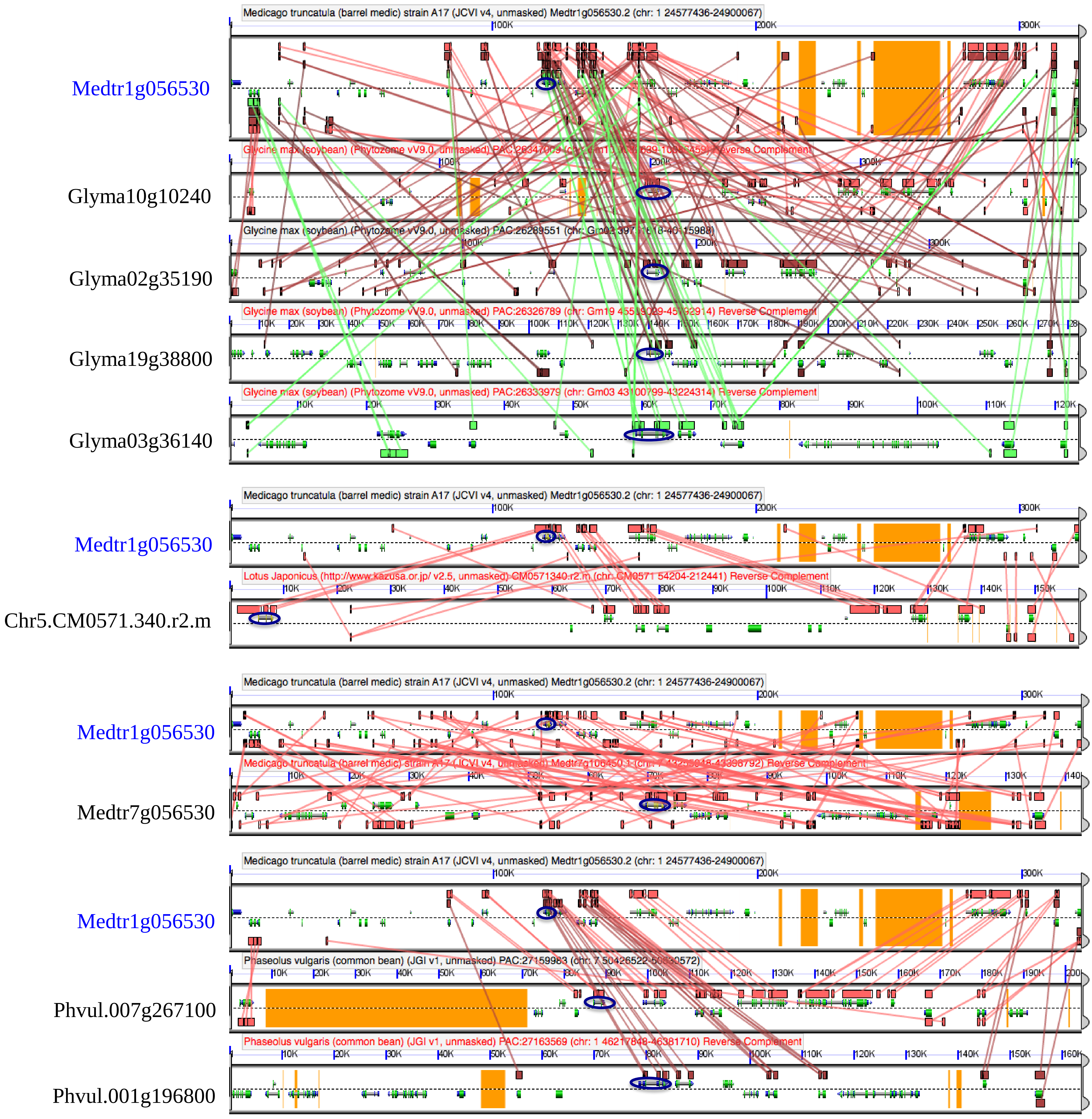

NF-YC1/2 orthologous genes in genomic regions of four legume plants

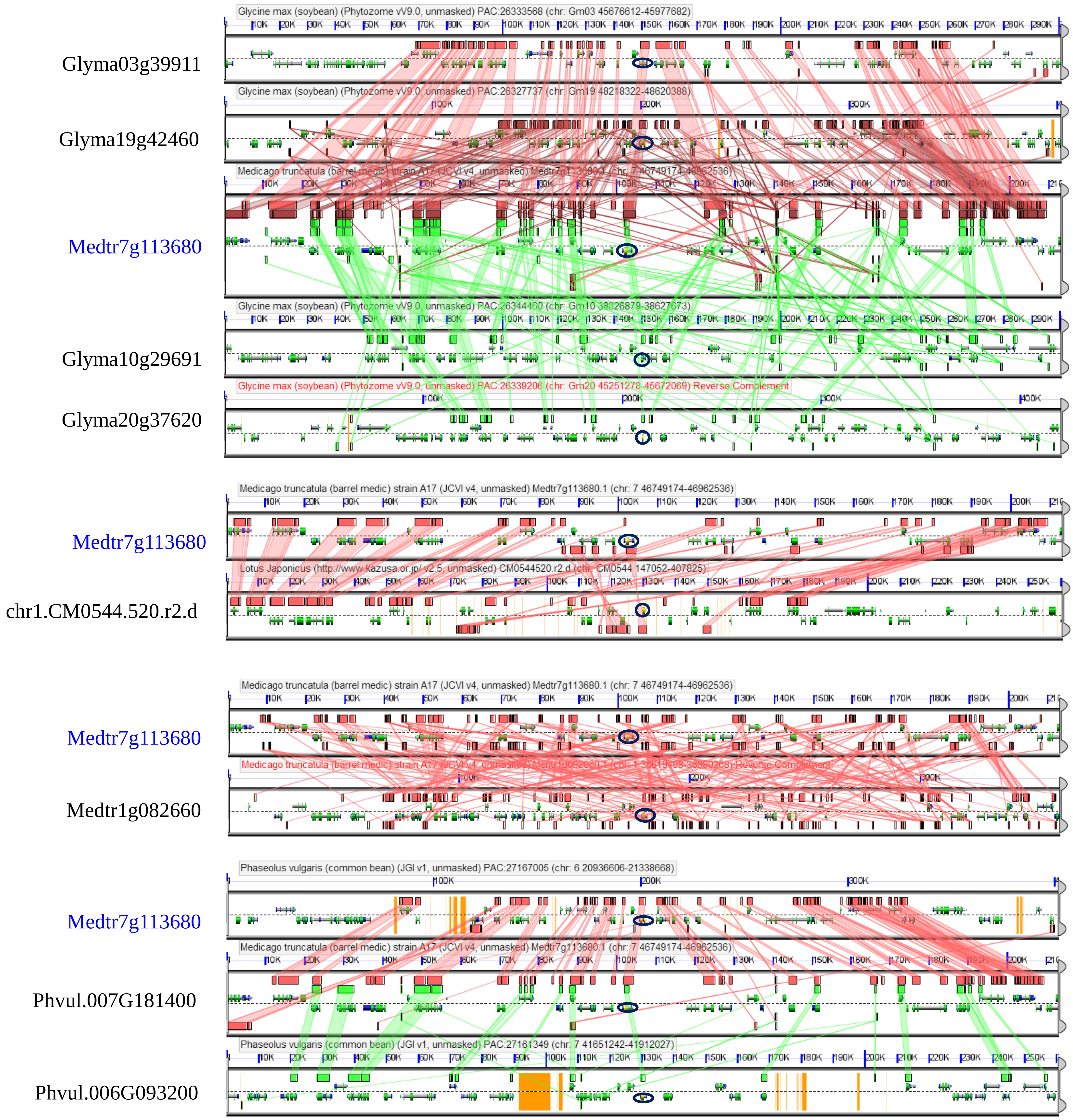

NIN orthologous genes in genomic regions of four legume plants

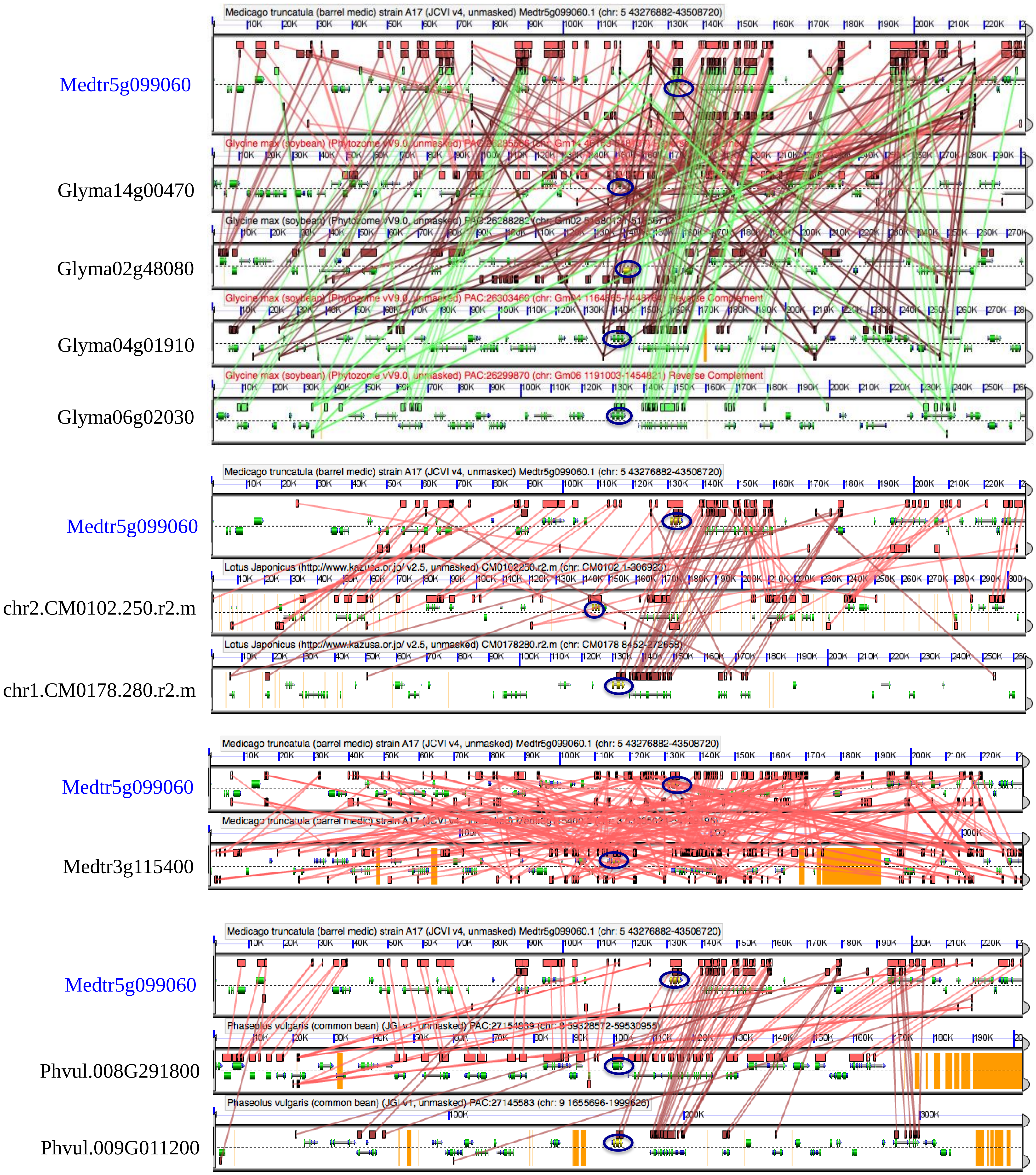

NIN2 orthologous genes in genomic regions of four legume plants

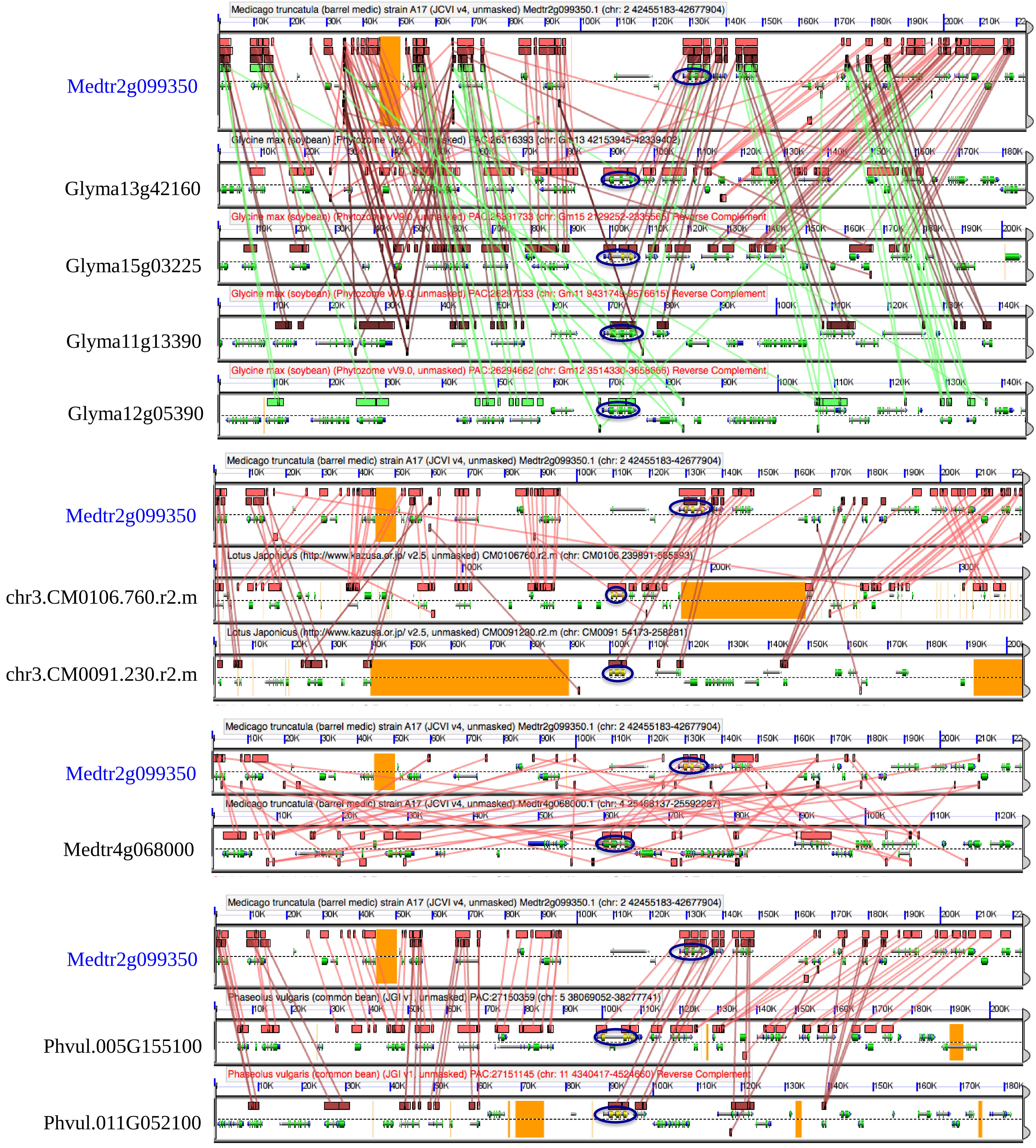

NSP1 orthologous genes in genomic regions of four legume plants

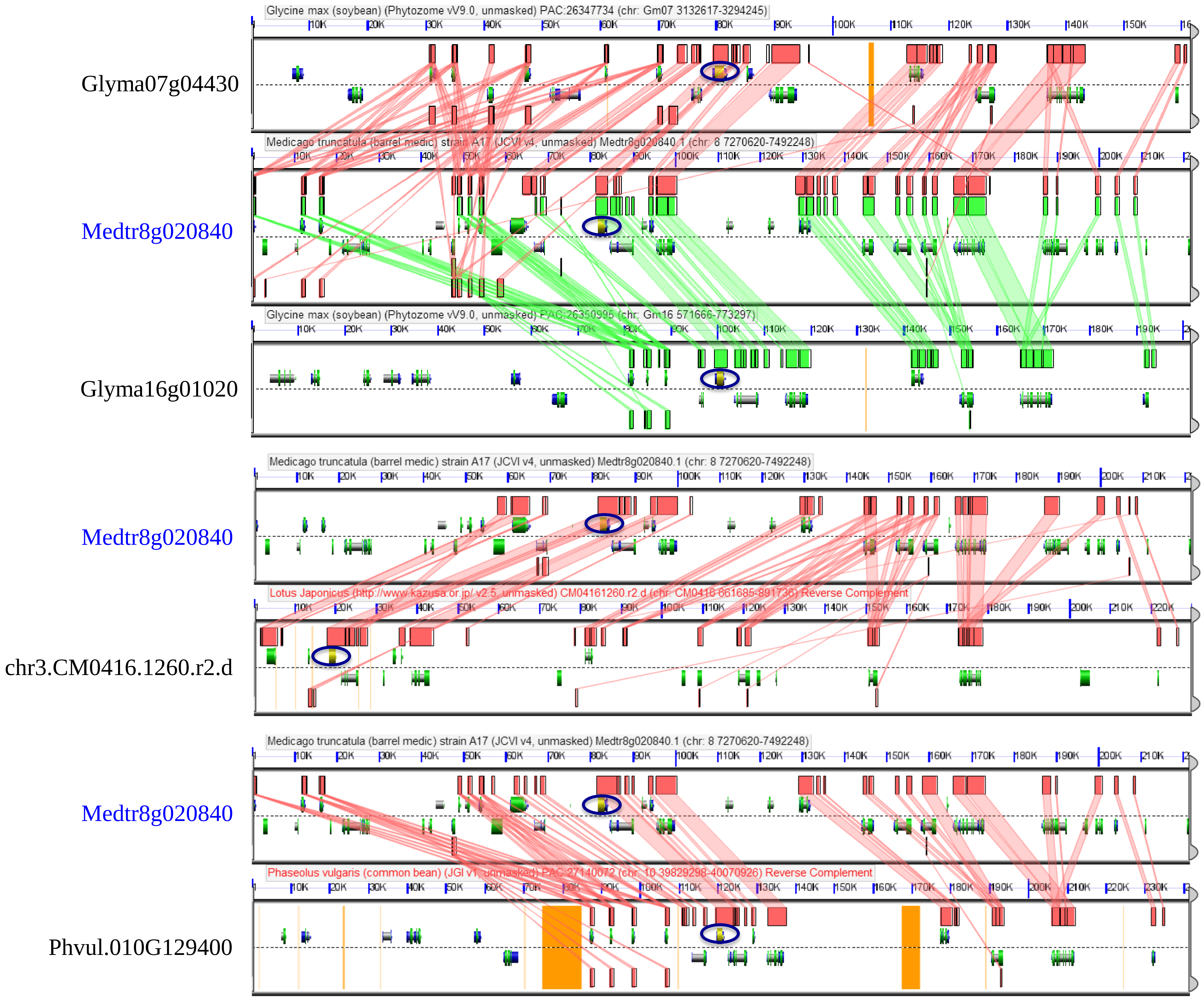

NSP2 orthologous genes in genomic regions of four legume plants

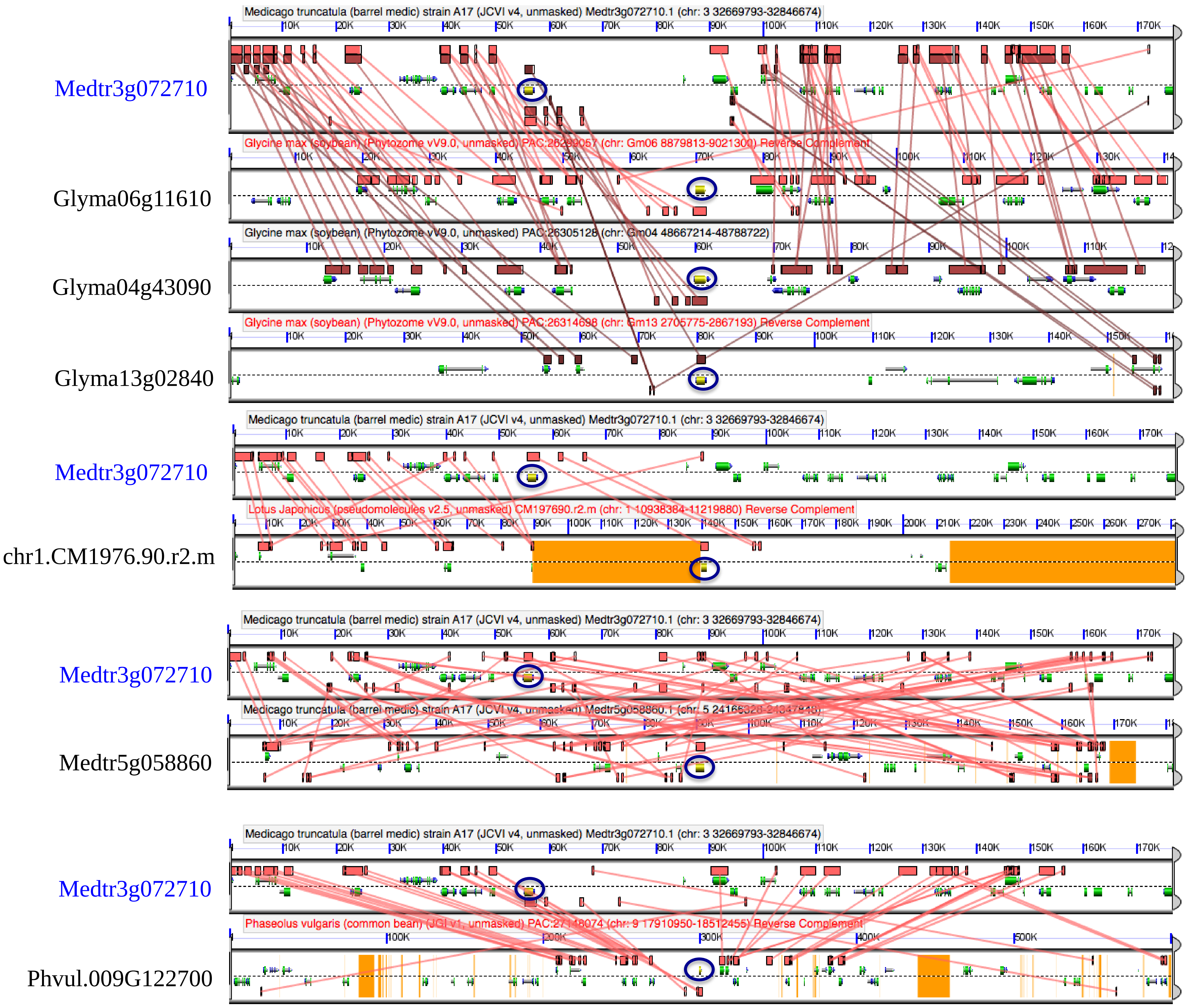

MtN6 orthologous genes in genomic regions of four legume plants

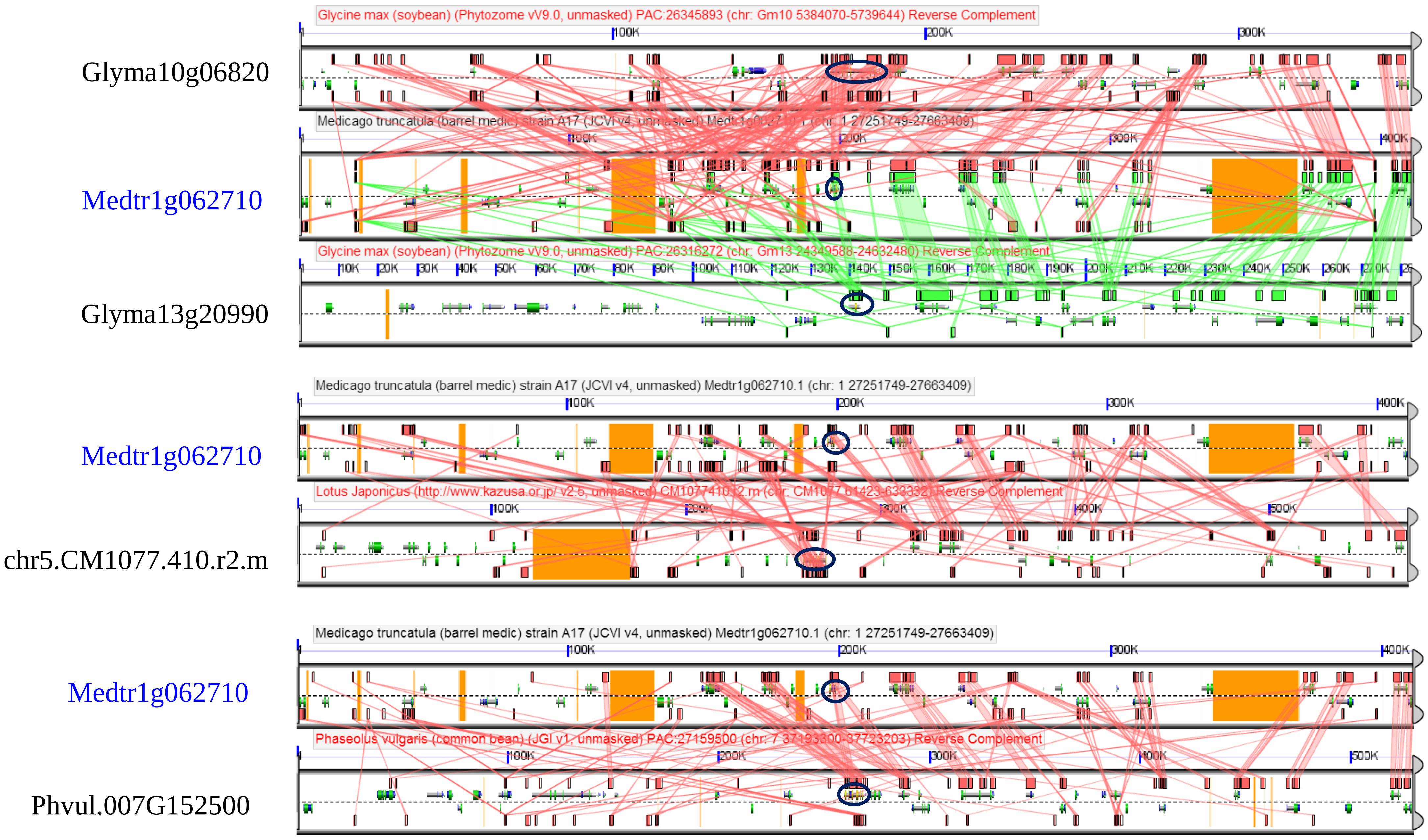

NODULIN-6I orthologous genes in genomic regions of four legume plants

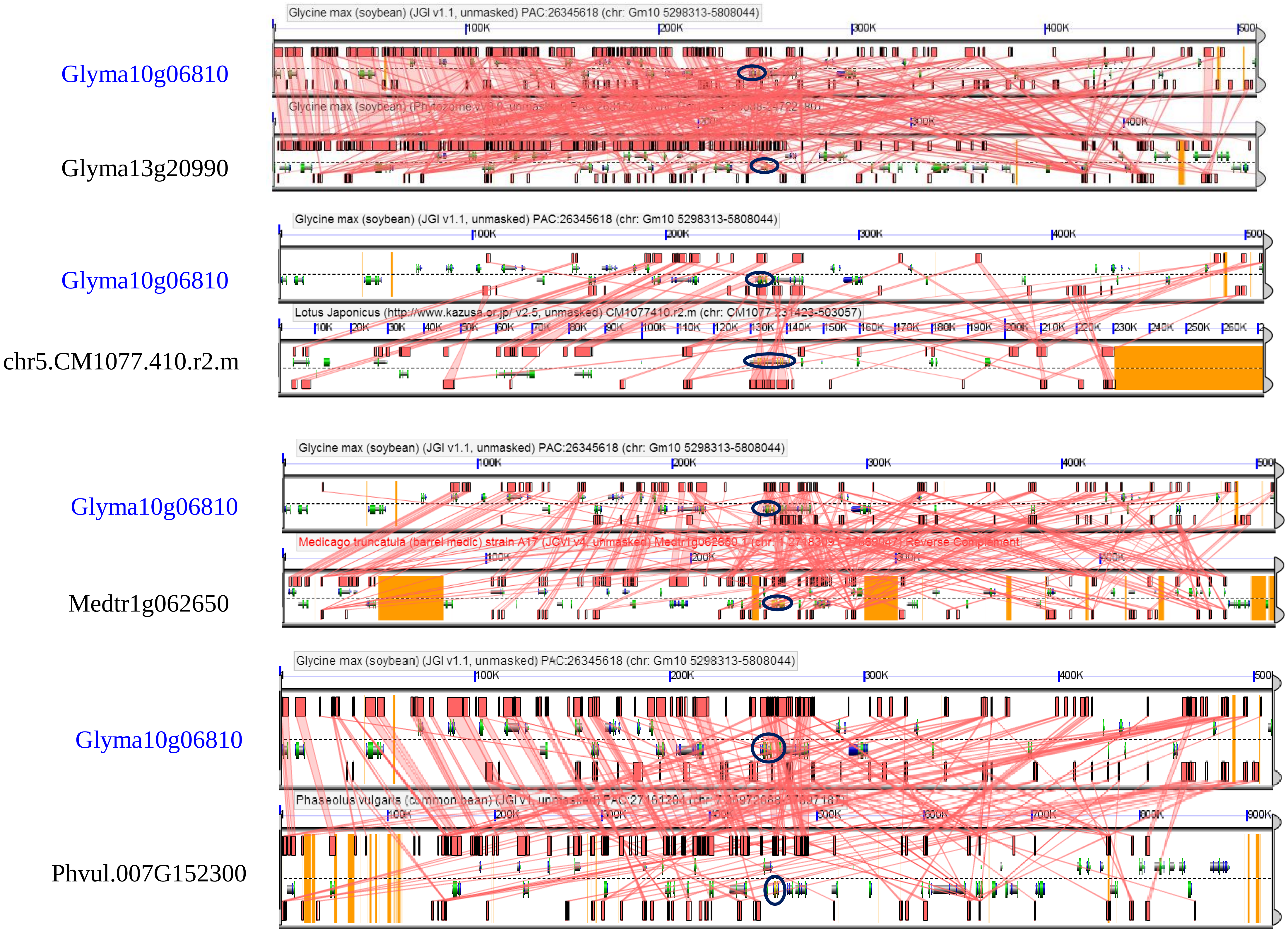

MtN13

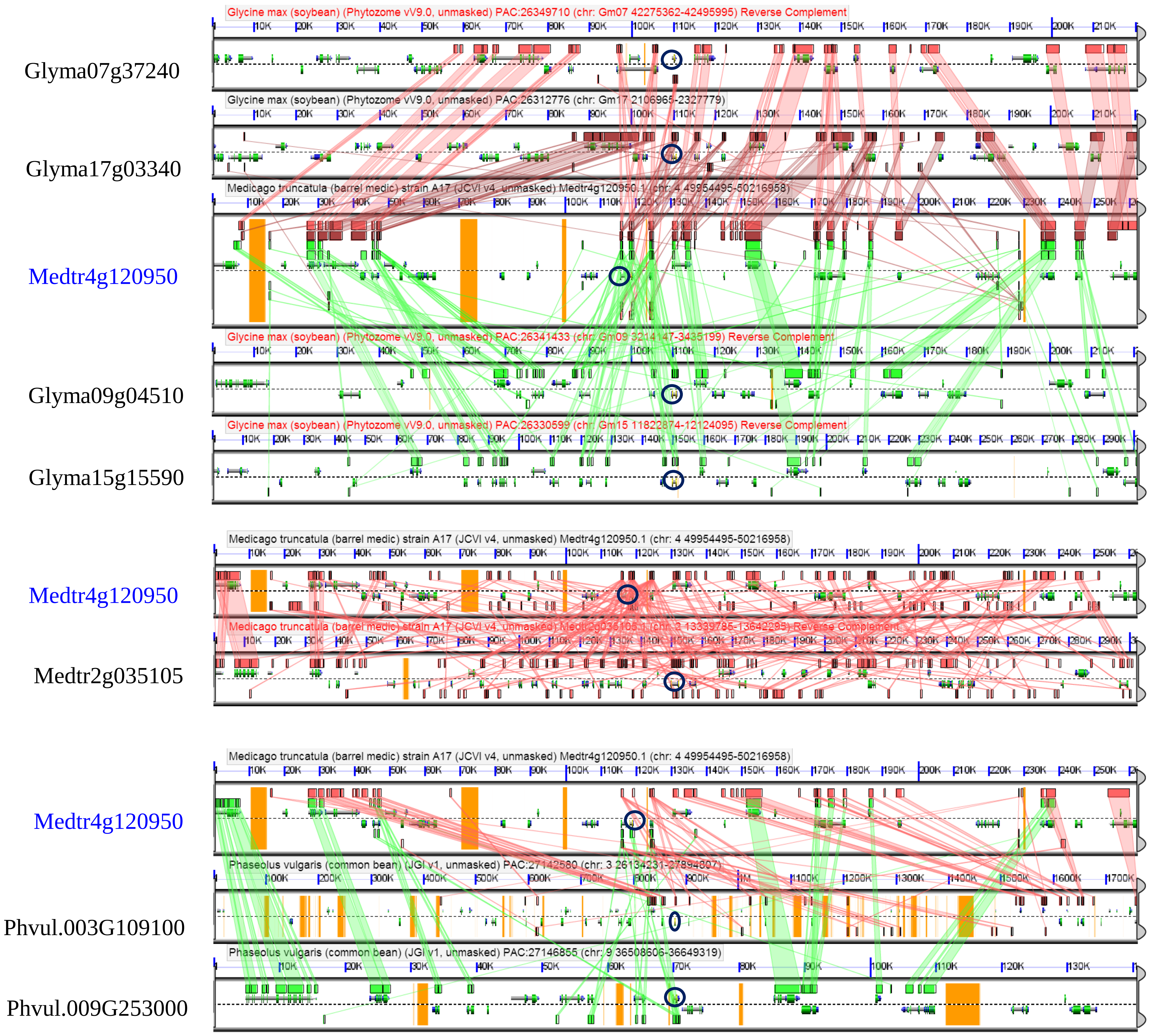

Glycine max (soybean) (Phytozome vV9.0, unmasked) PAC:26349710 (chr: Gm07 42275362-42495995) Reverse Complement
Glyma07g37240
Glycine max (soybean) (Phytozome vV9.0, unmasked) PAC:26312776 (chr: Gm17 2106965-2327779)
Glyma17g03340
Medicago truncatula (barrel medic) strain A17 (JCVI v4, unmasked) Medtr4g120950.1 (chr: 4 49954495-50216958)
Medtr4g120950
Glyma09g04510
Glyma15g15590
Medicago truncatula (barrel medic) strain A17 (JCVI v4, unmasked) Medtr4g120950.1 (chr: 4 49954495-50216958)
Medtr4g120950
Medtr2g035105
Medicago truncatula (barrel medic) strain A17 (JCVI v4, unmasked) Medtr4g120950.1 (chr: 4 49954495-50216958)
Medtr4g120950
Phaseolus vulgaris (common bean) (JGI v1, unmasked) PAC:27142580 (chr: 3 26134231-27894807)
Phvul.003G109100
Phaseolus vulgaris (common bean) (JGI v1, unmasked) PAC:27146855 (chr: 9 36508606-36649319)
Phvul.009G253000

NFR1 orthologous genes in genomic regions of four legume plants

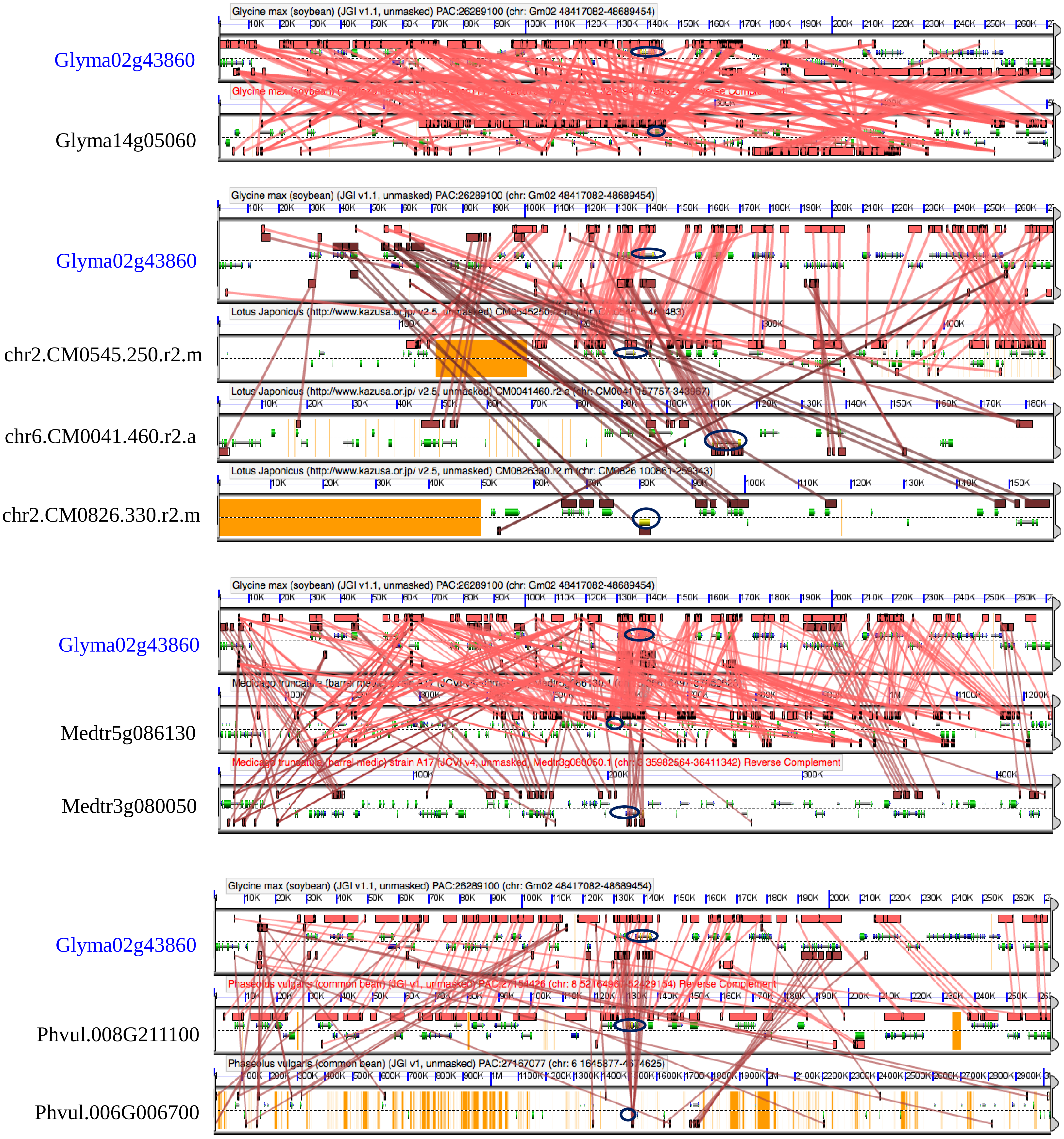

NFR5 orthologous genes in genomic regions of four legume plants

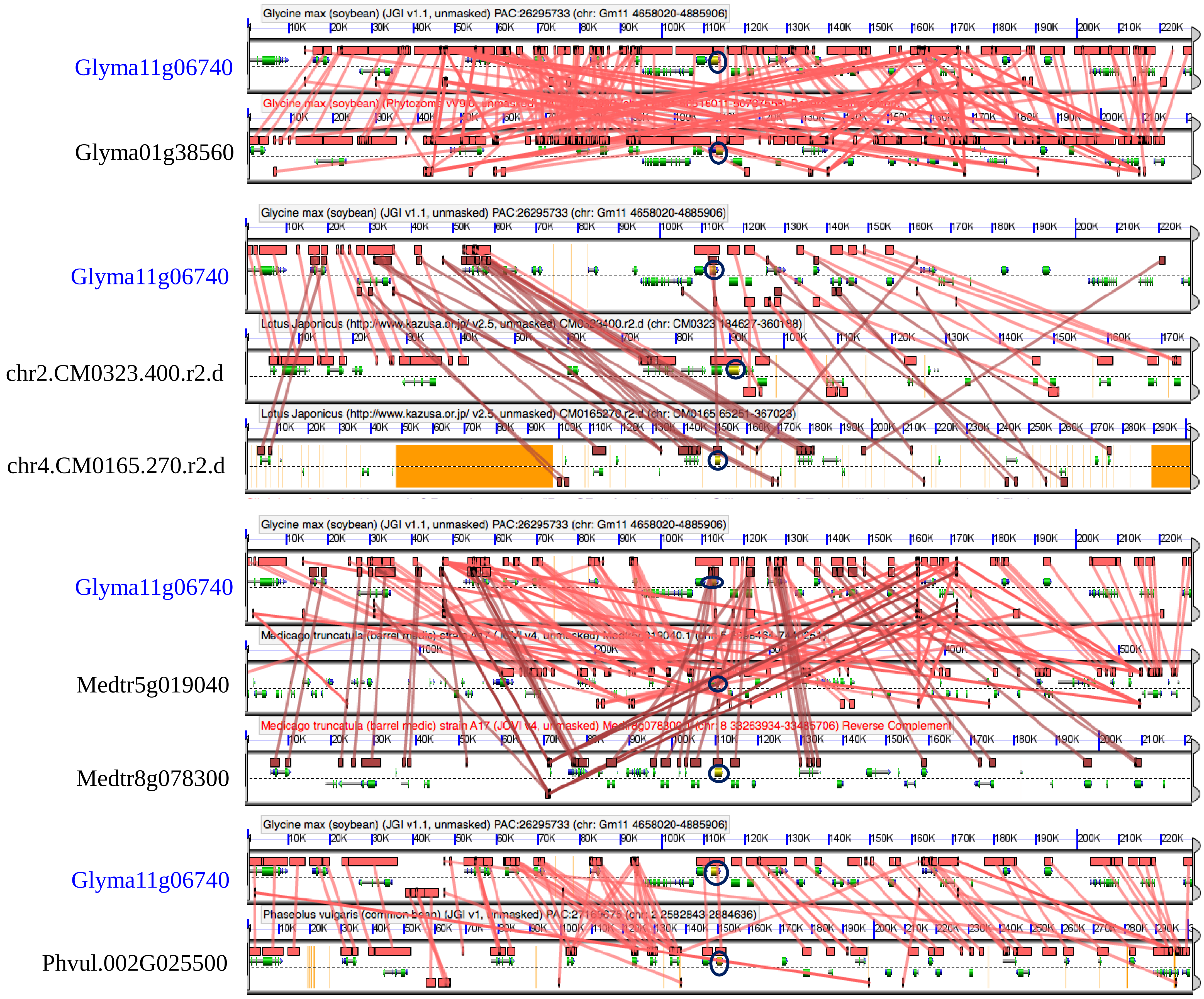

SYMRK /NORK orthologous genes in genomic regions of four legume plants

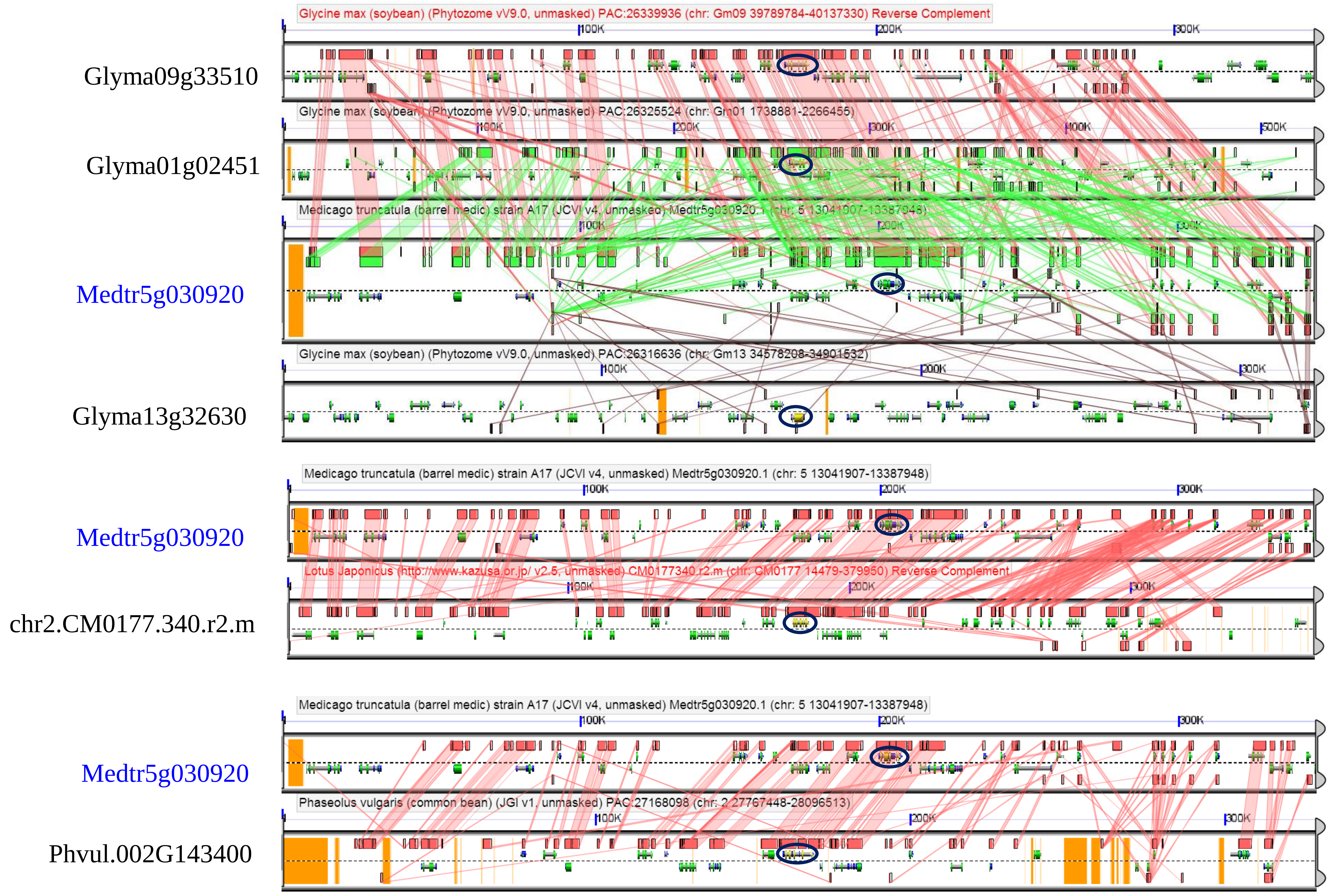

POLLUX orthologous genes in genomic regions of four legume plants

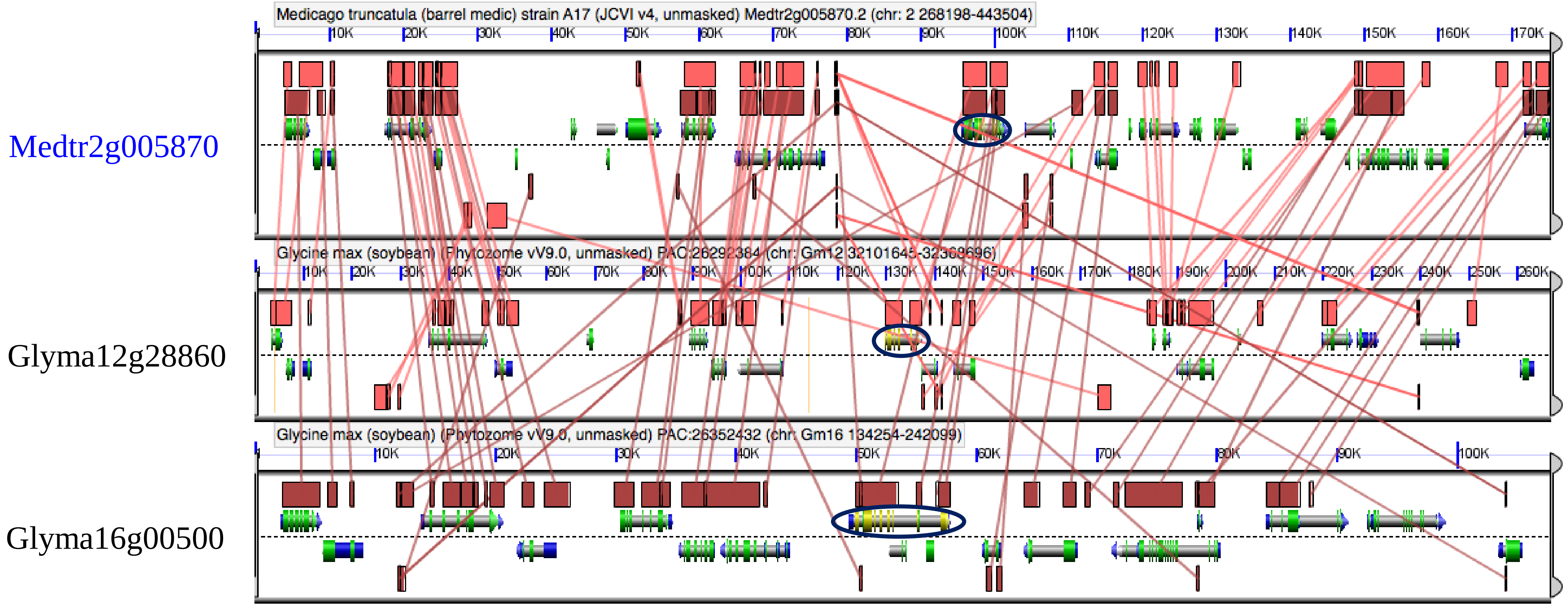

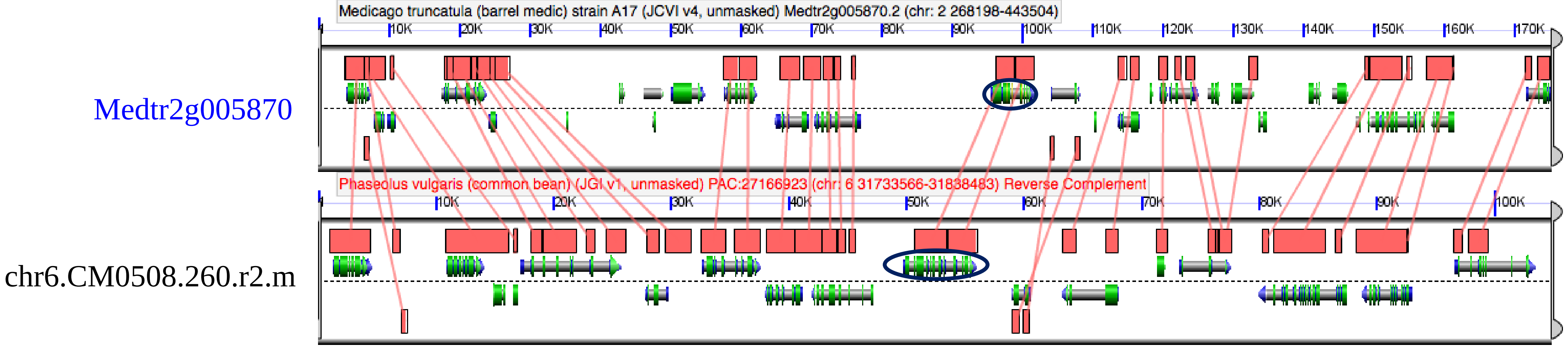

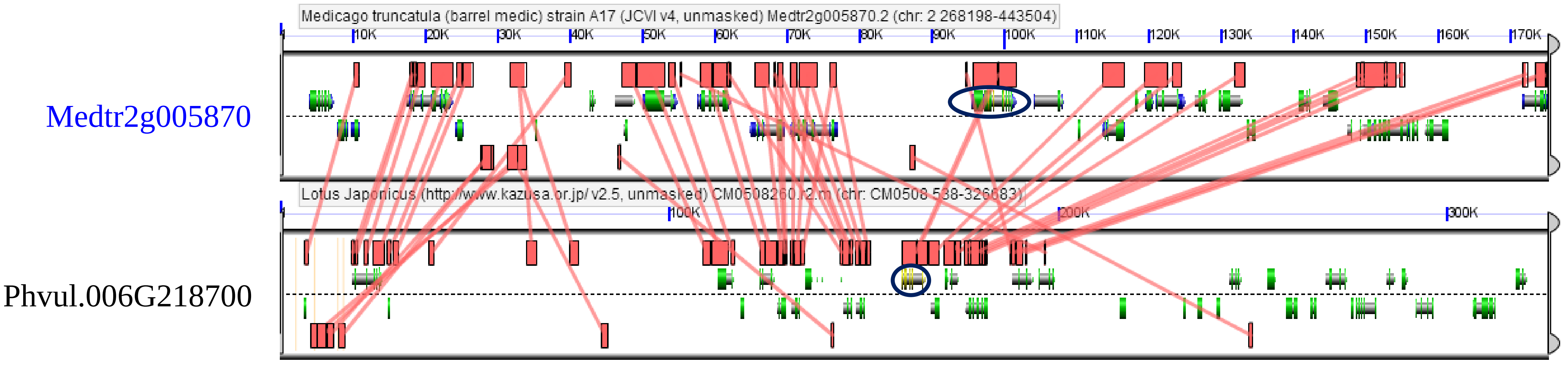

CASTOR orthologous genes in genomic regions of four legume plants

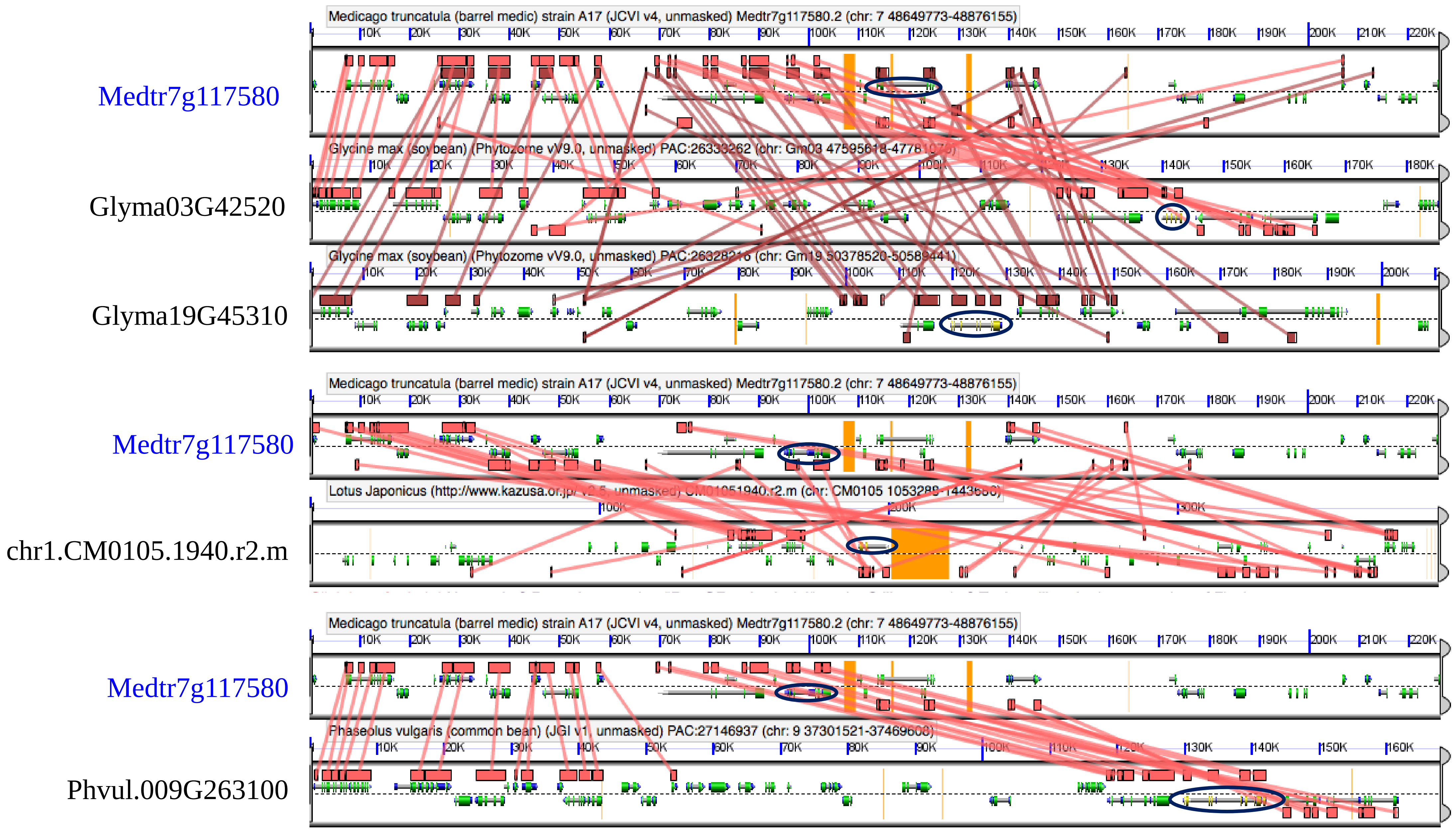

NUP85 orthologous genes in genomic regions of four legume plants

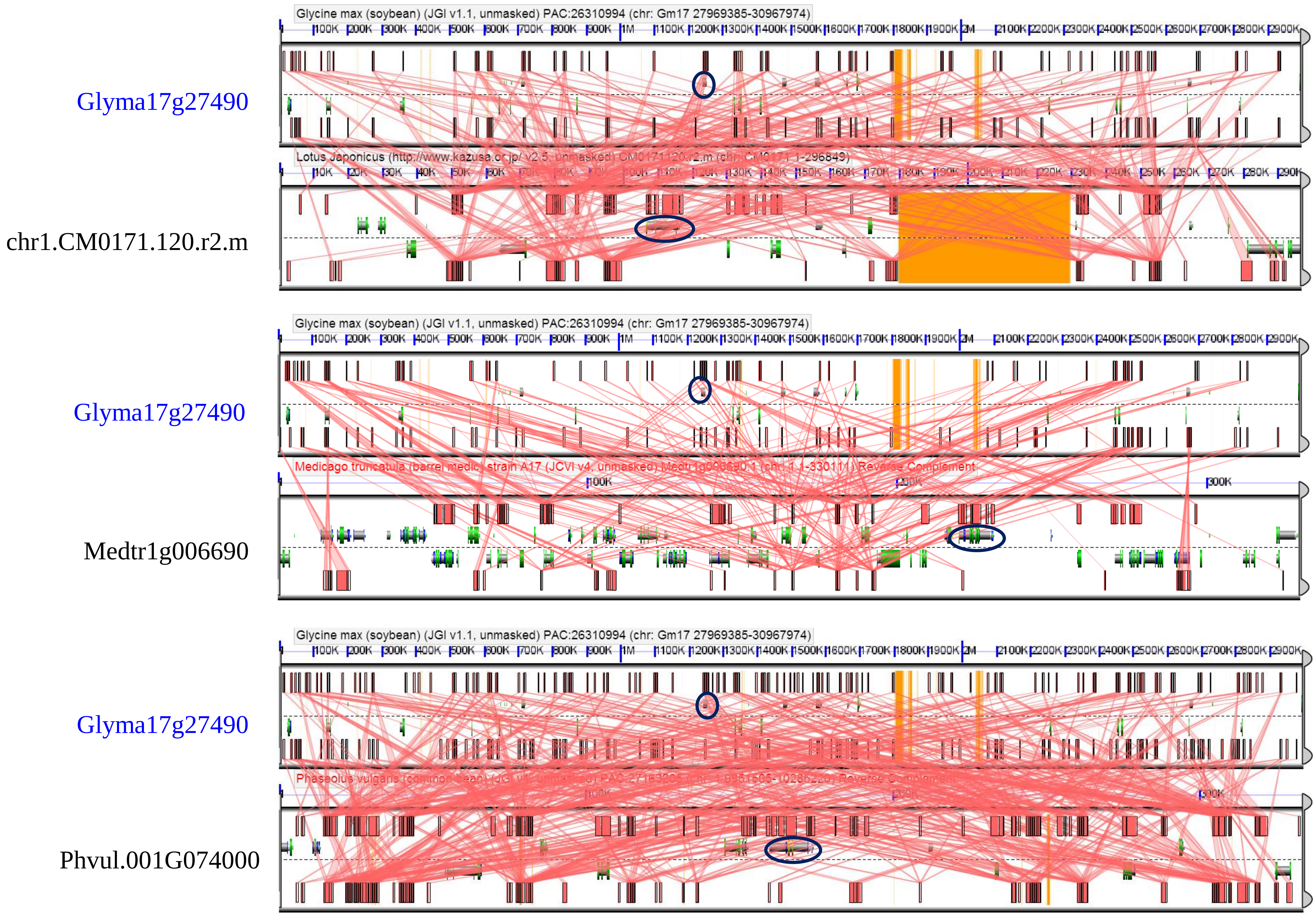

NUP133 orthologous genes in genomic regions of four legume plants

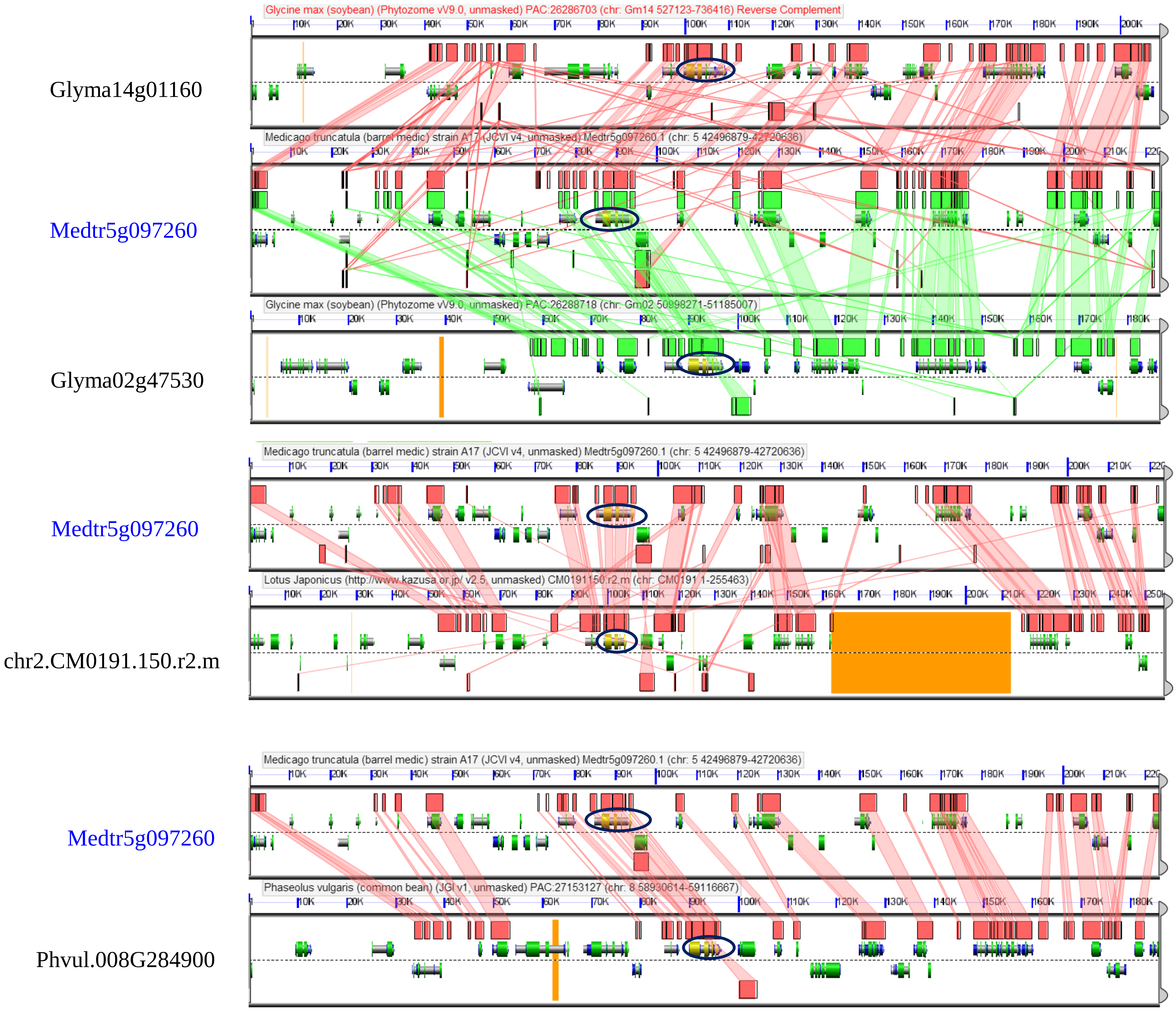

CCaMK/DMI3 orthologous genes in genomic regions of four legume plants

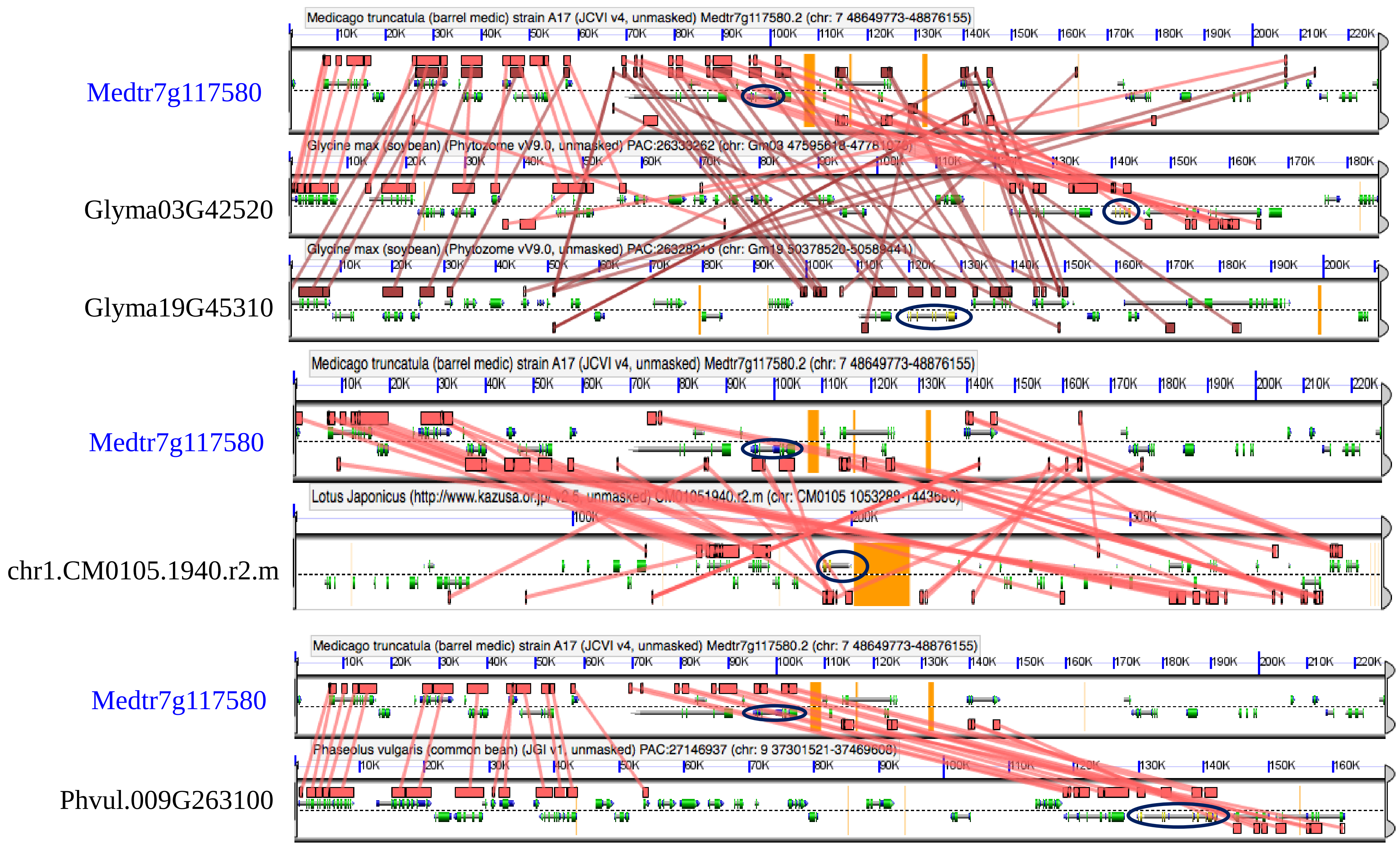

SUNN NARK/HAR1 orthologous genes in genomic regions of four legume plants

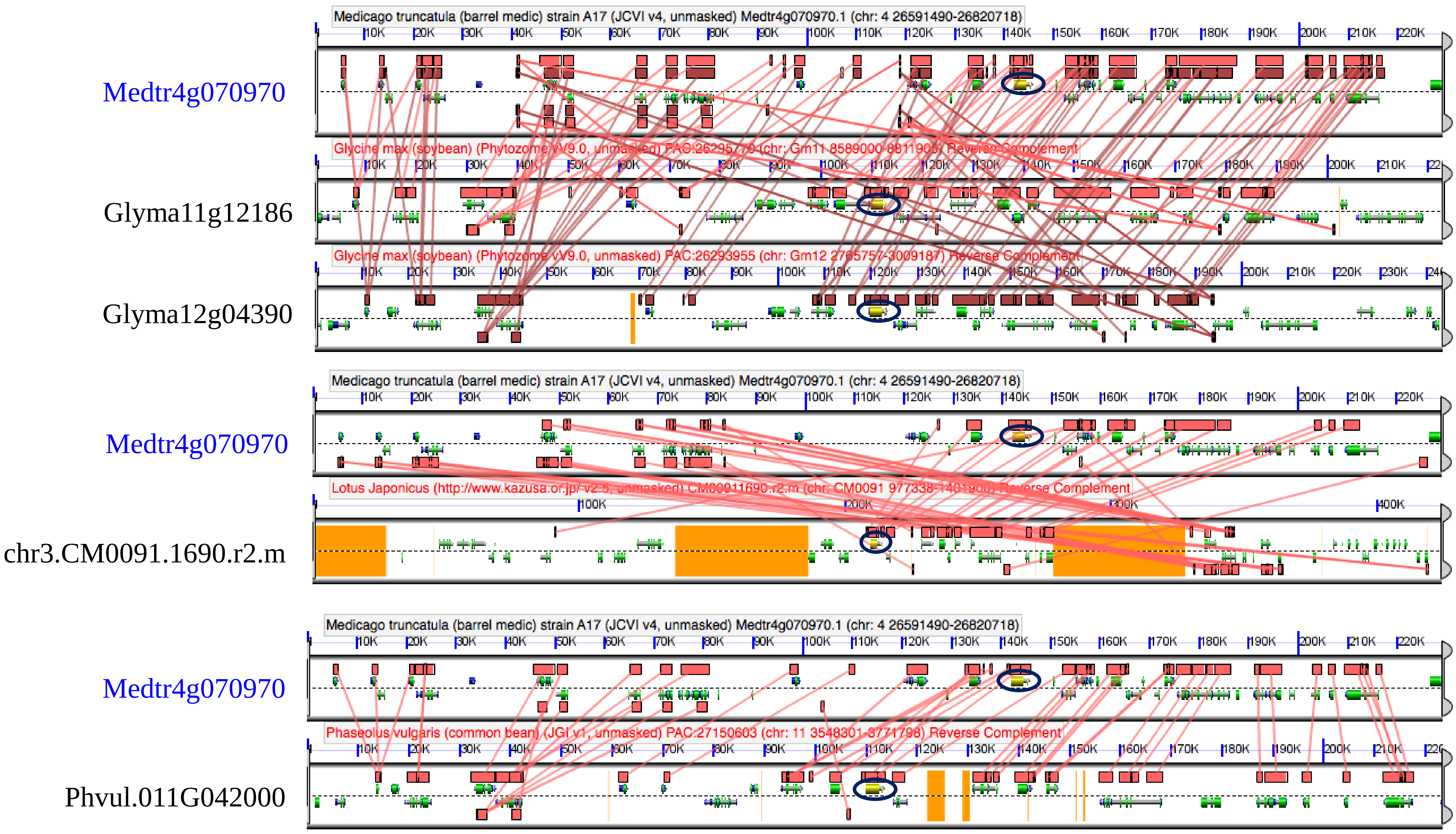

CCS52 orthologous genes in genomic regions of four legume plants

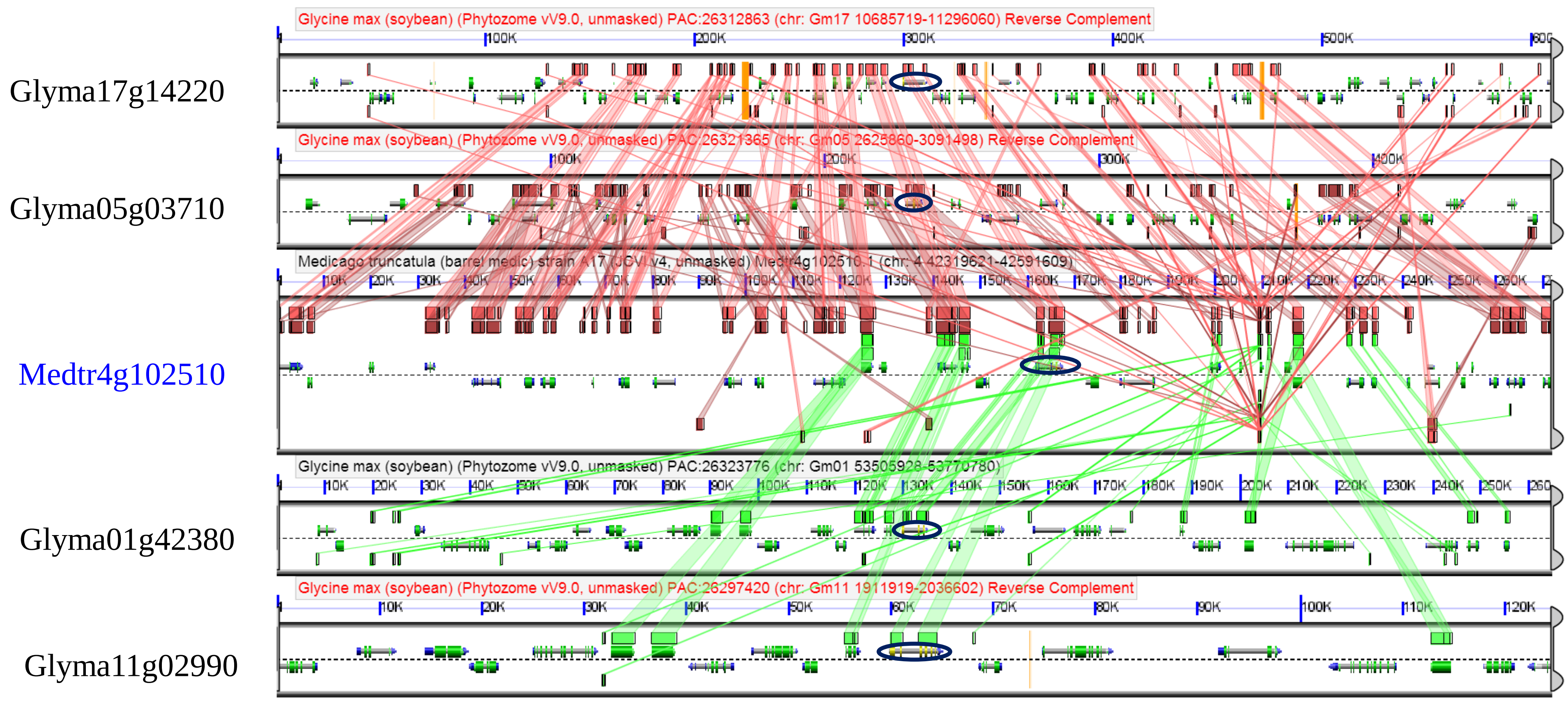

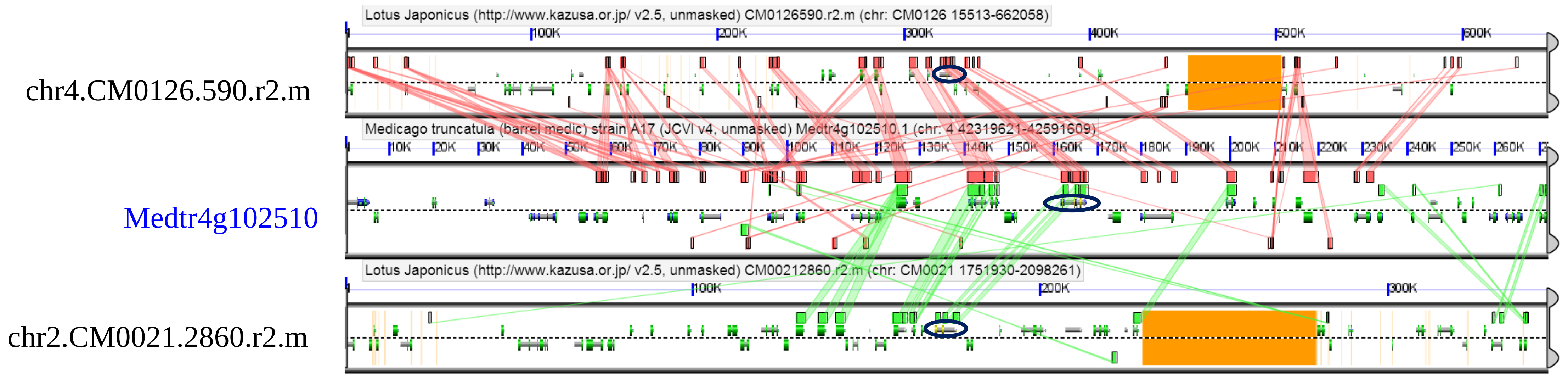

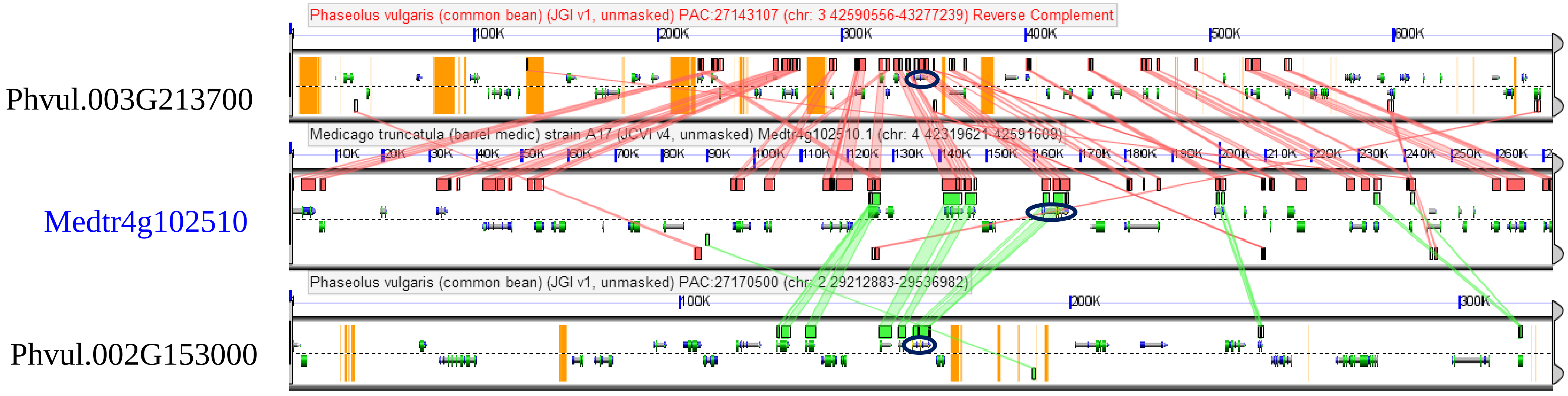

CYCLOPS/IPD3 orthologous genes in genomic regions of four legume plants

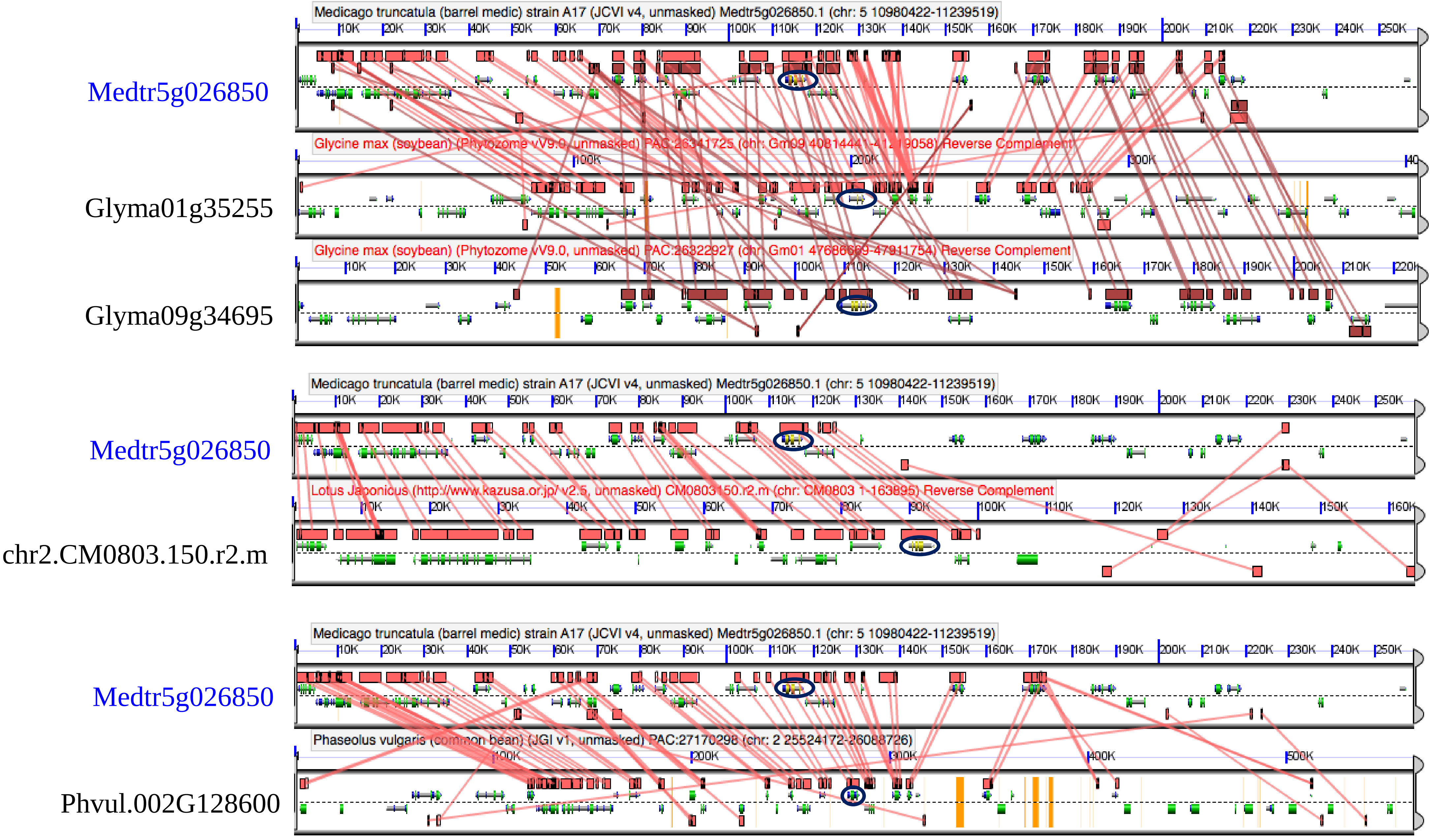

SAN1A orthologous genes in genomic regions of four legume plants

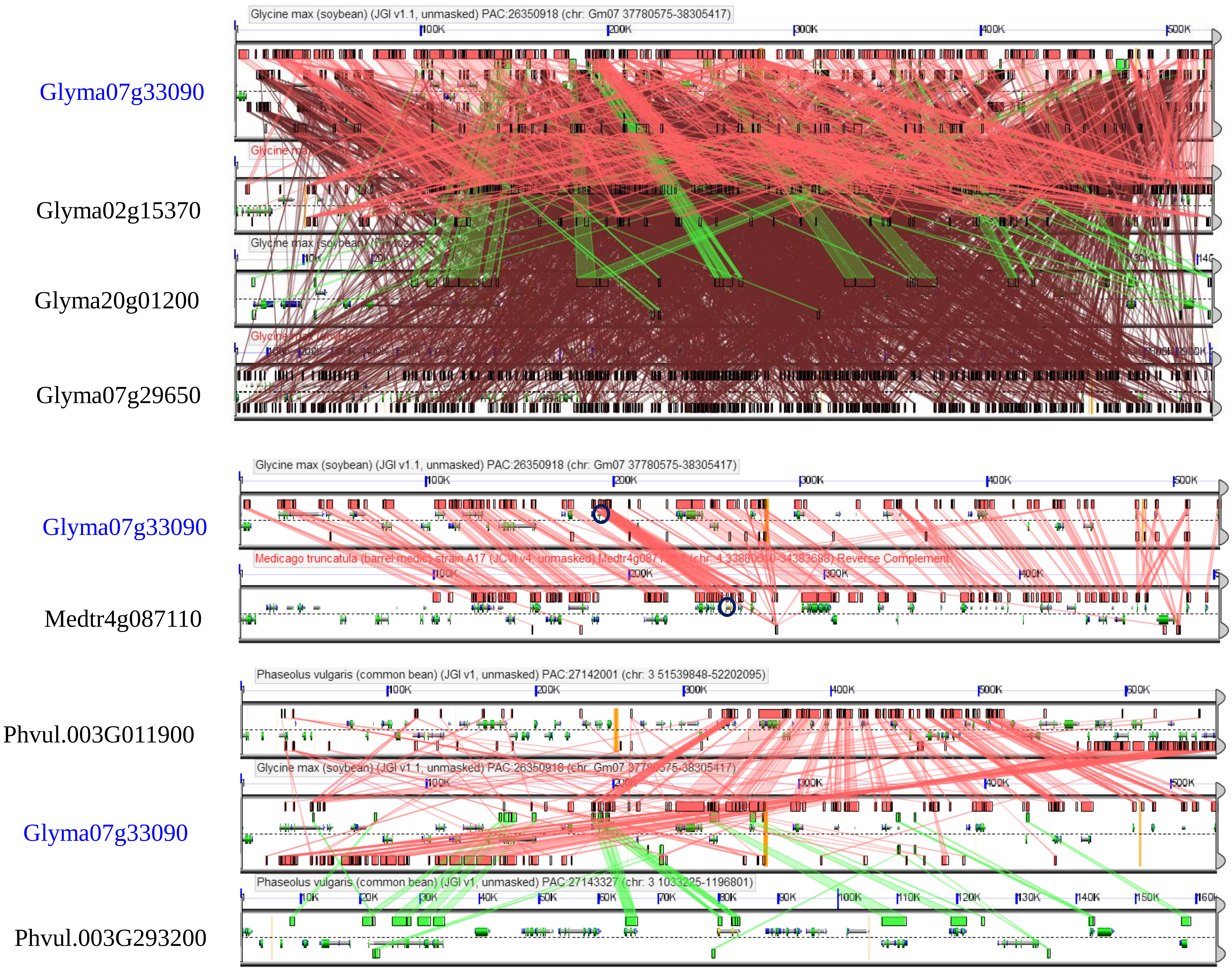

SAN1B orthologous genes in genomic regions of four legume plants

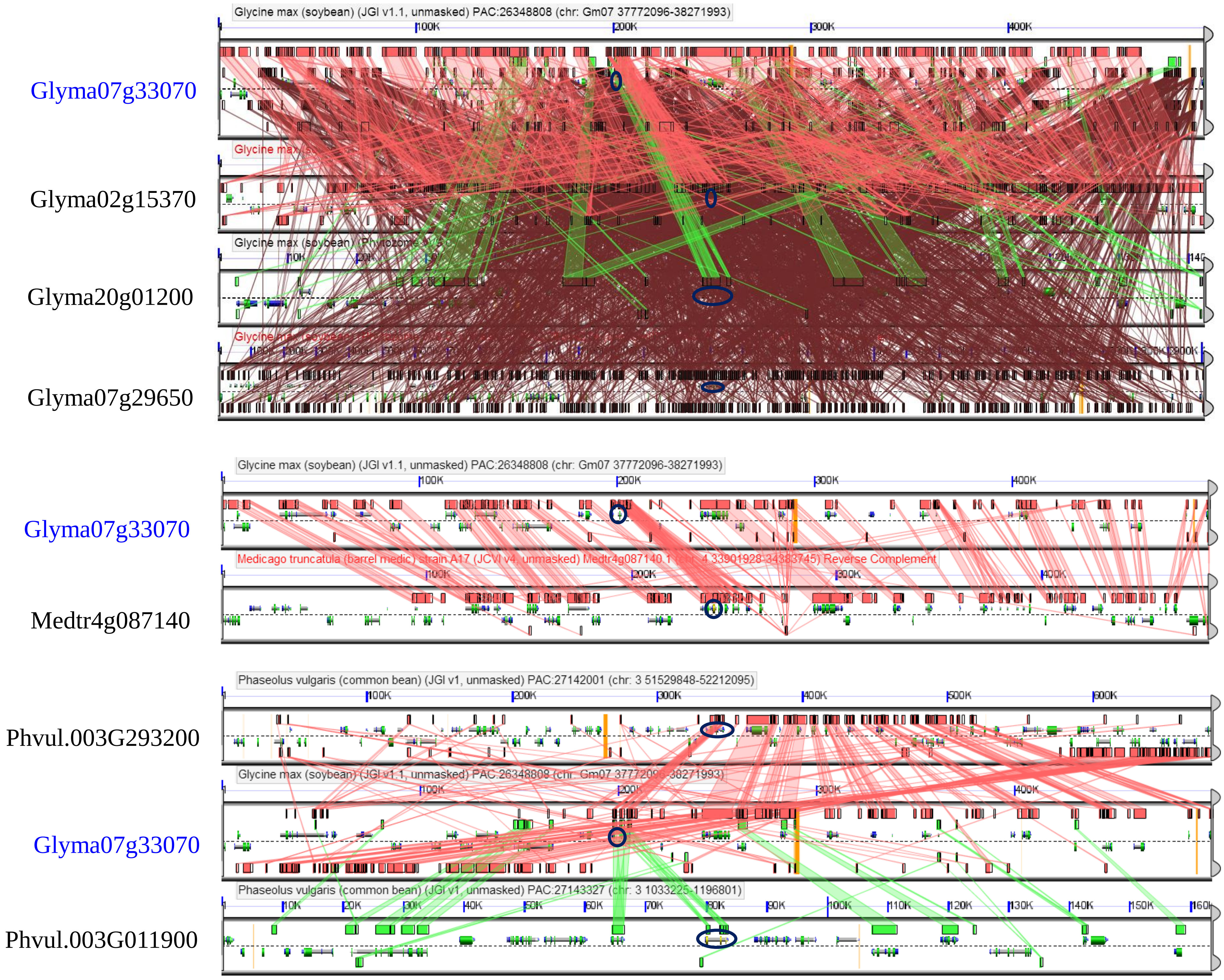

SIN1 orthologous genes in genomic regions of four legume plants

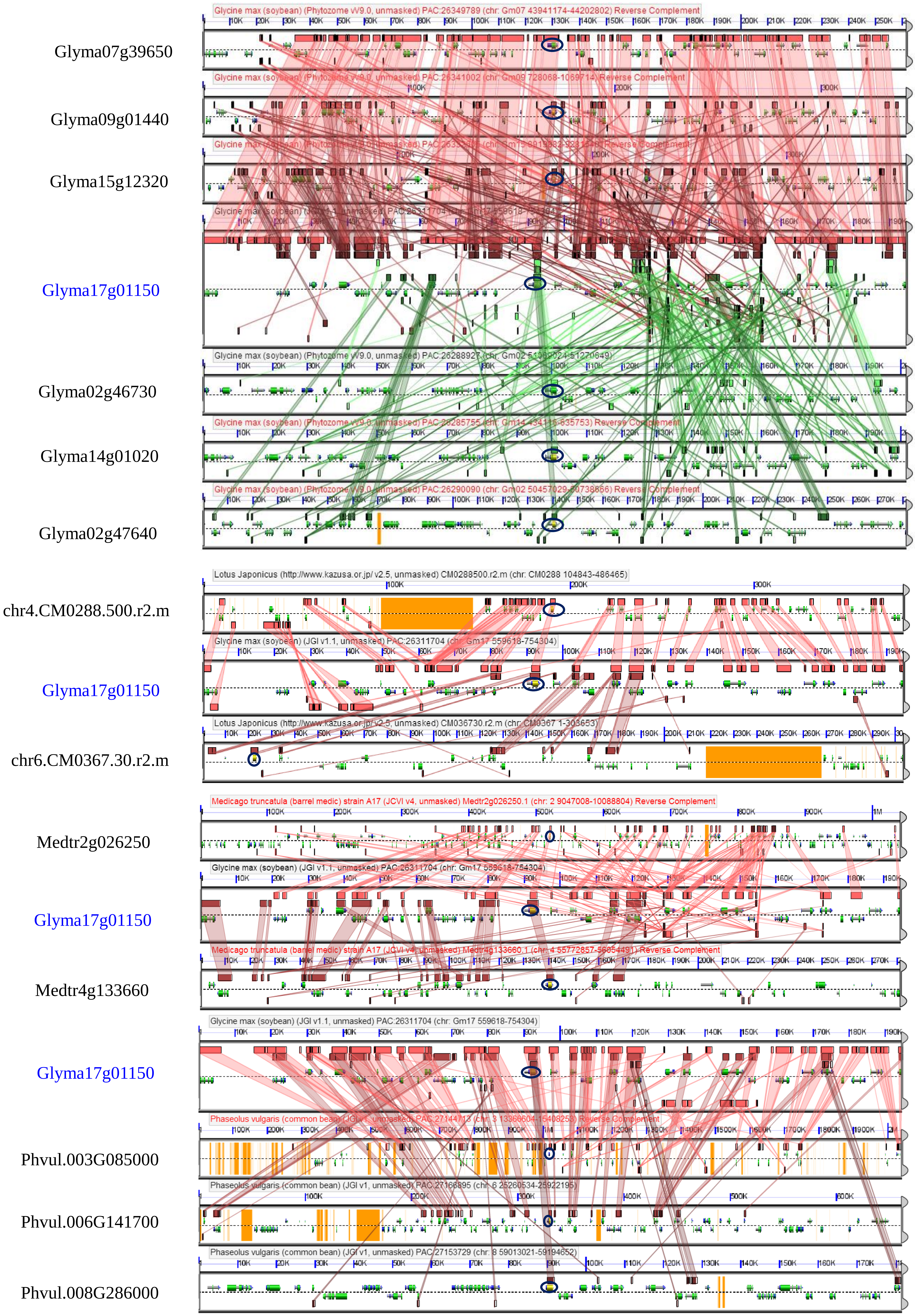

GmRIC1 orthologous genes in genomic regions of four legume plants

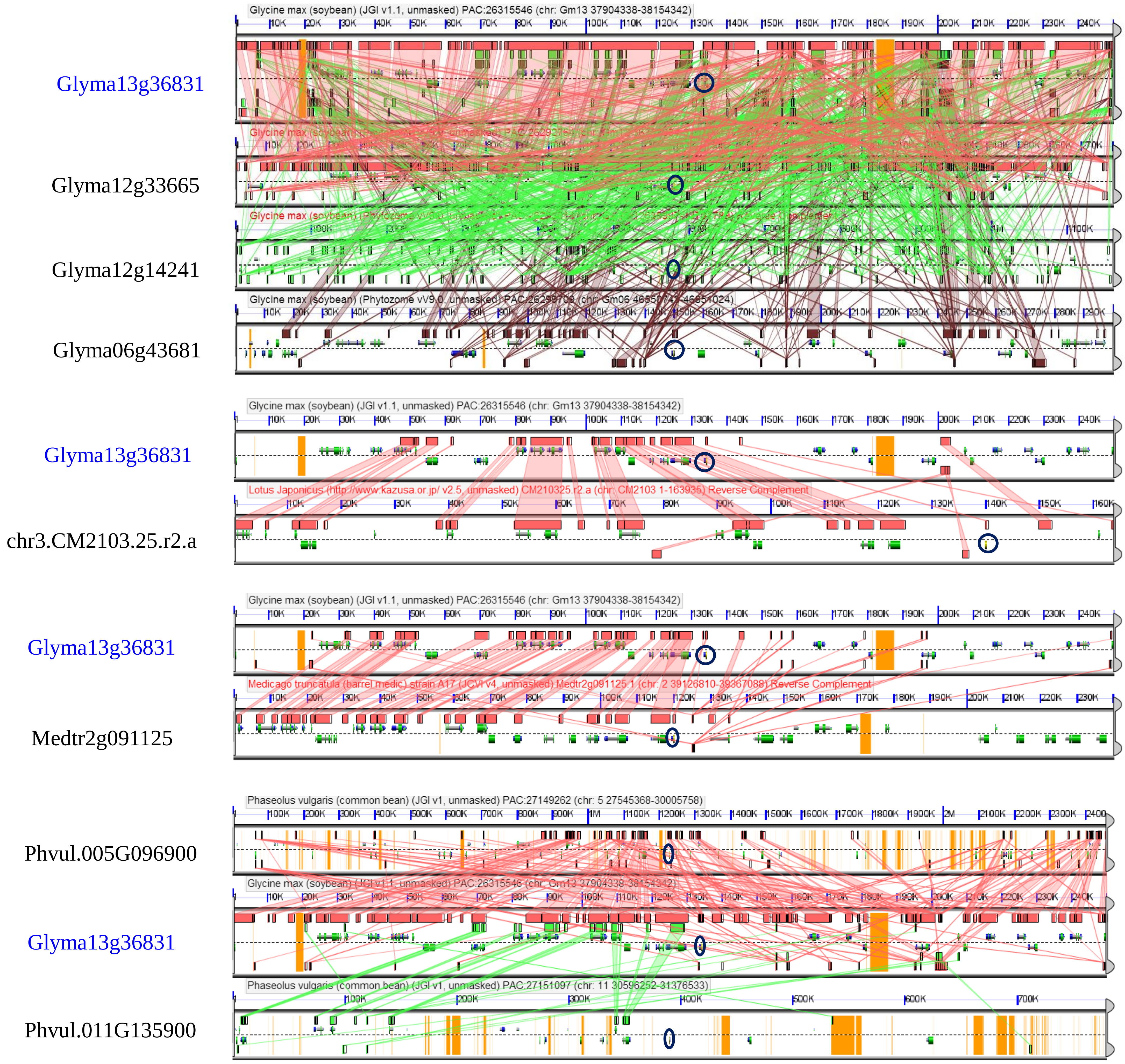

GmNIC1 orthologous genes in genomic regions of four legume plants

Glyma12g33660

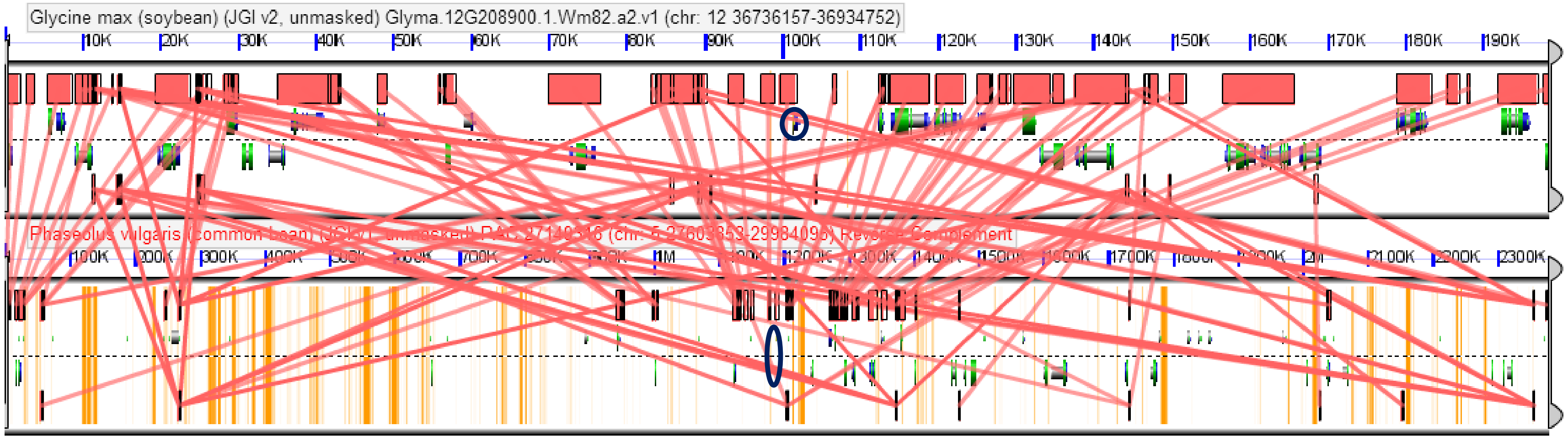

Phvul.005G097000

FWL1 orthologous genes in genomic regions of four legume plants

Medtr6g084940

Glyma09g31910

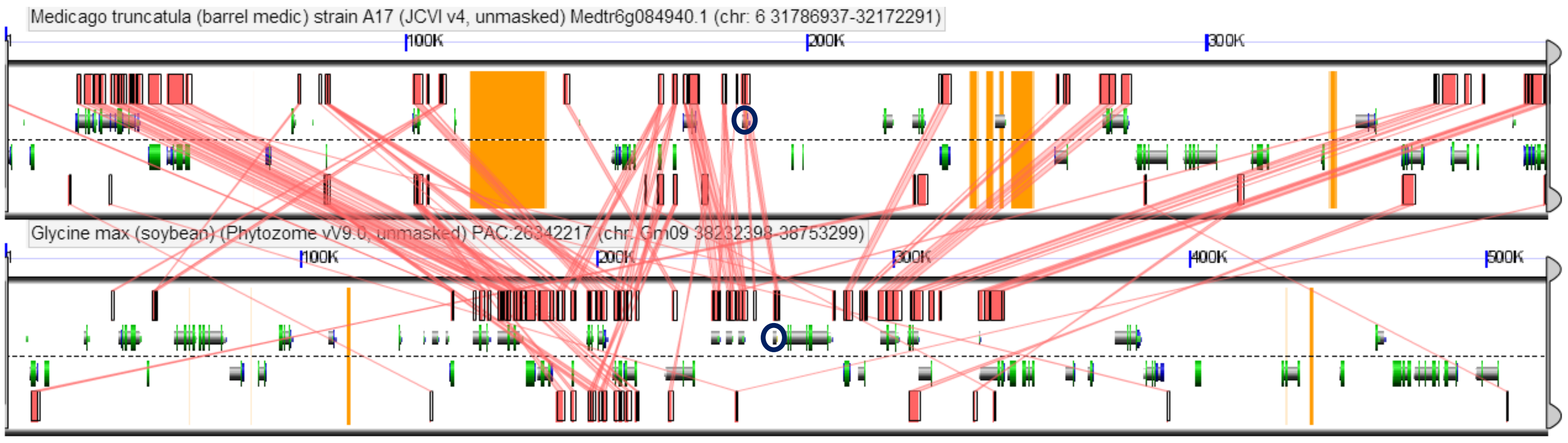

ERN1/2 orthologous genes in genomic regions of four legume plants

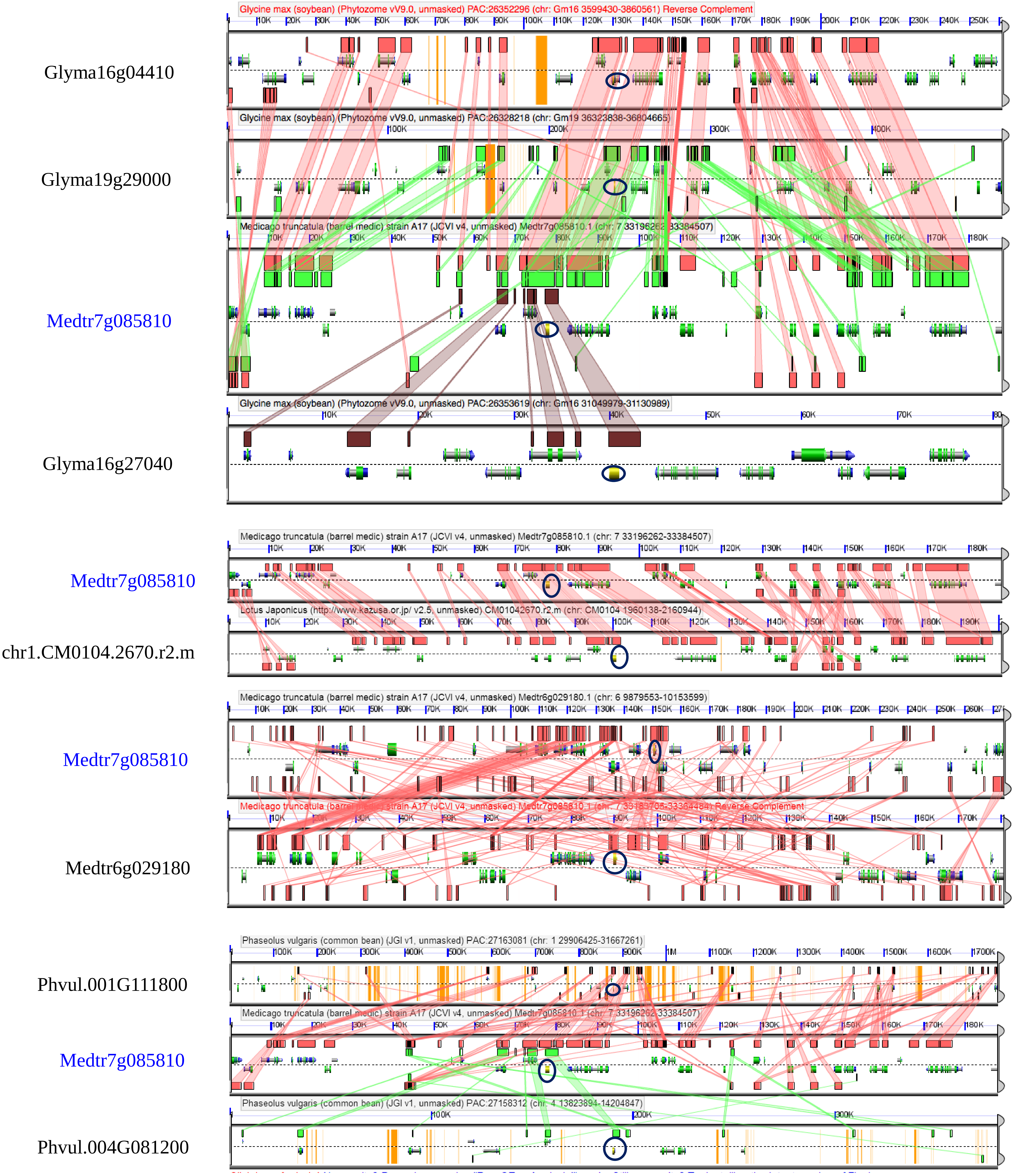

ERN3 orthologous genes in genomic regions of four legume plants

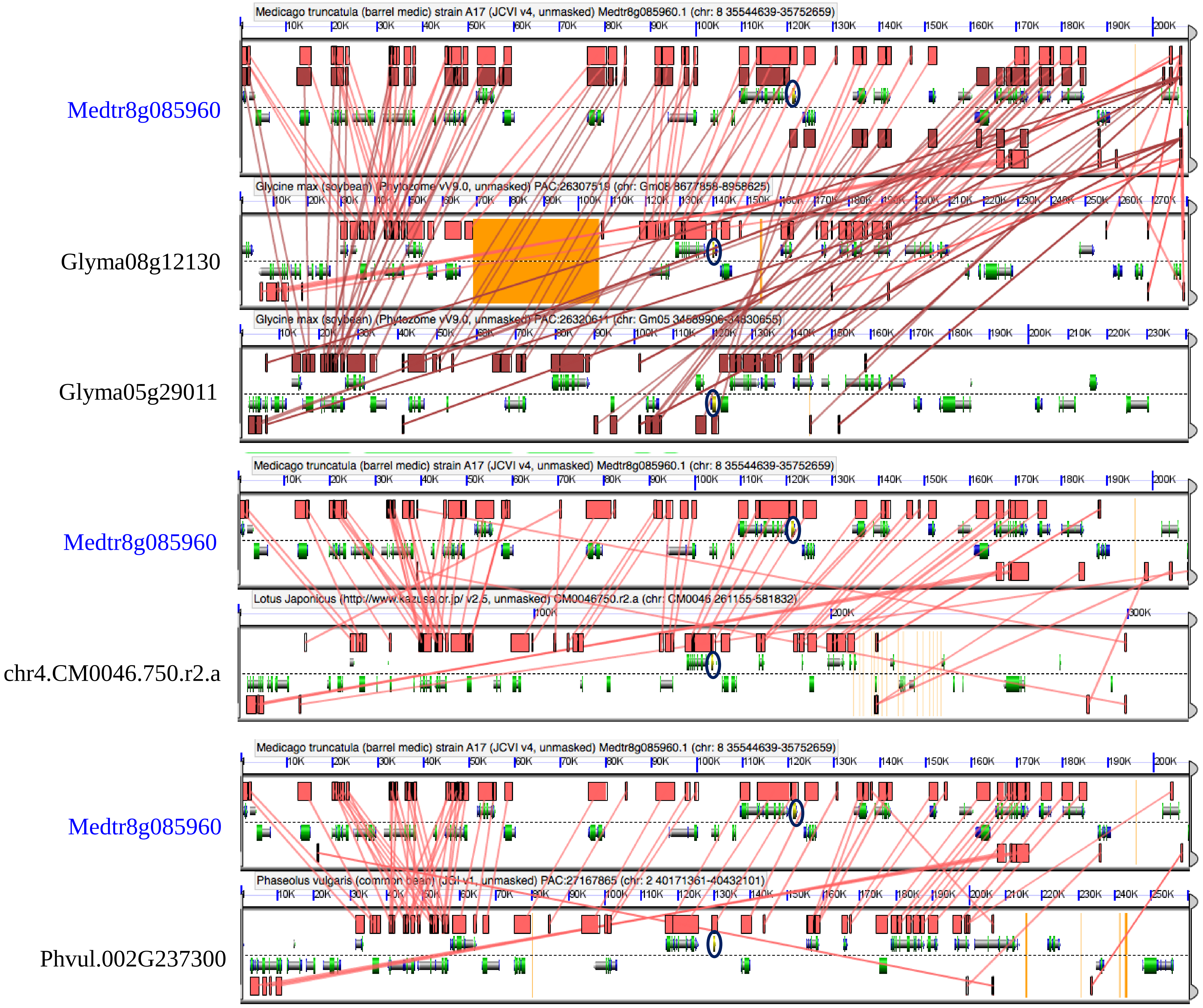

MtLIN orthologous genes in genomic regions of four legume plants

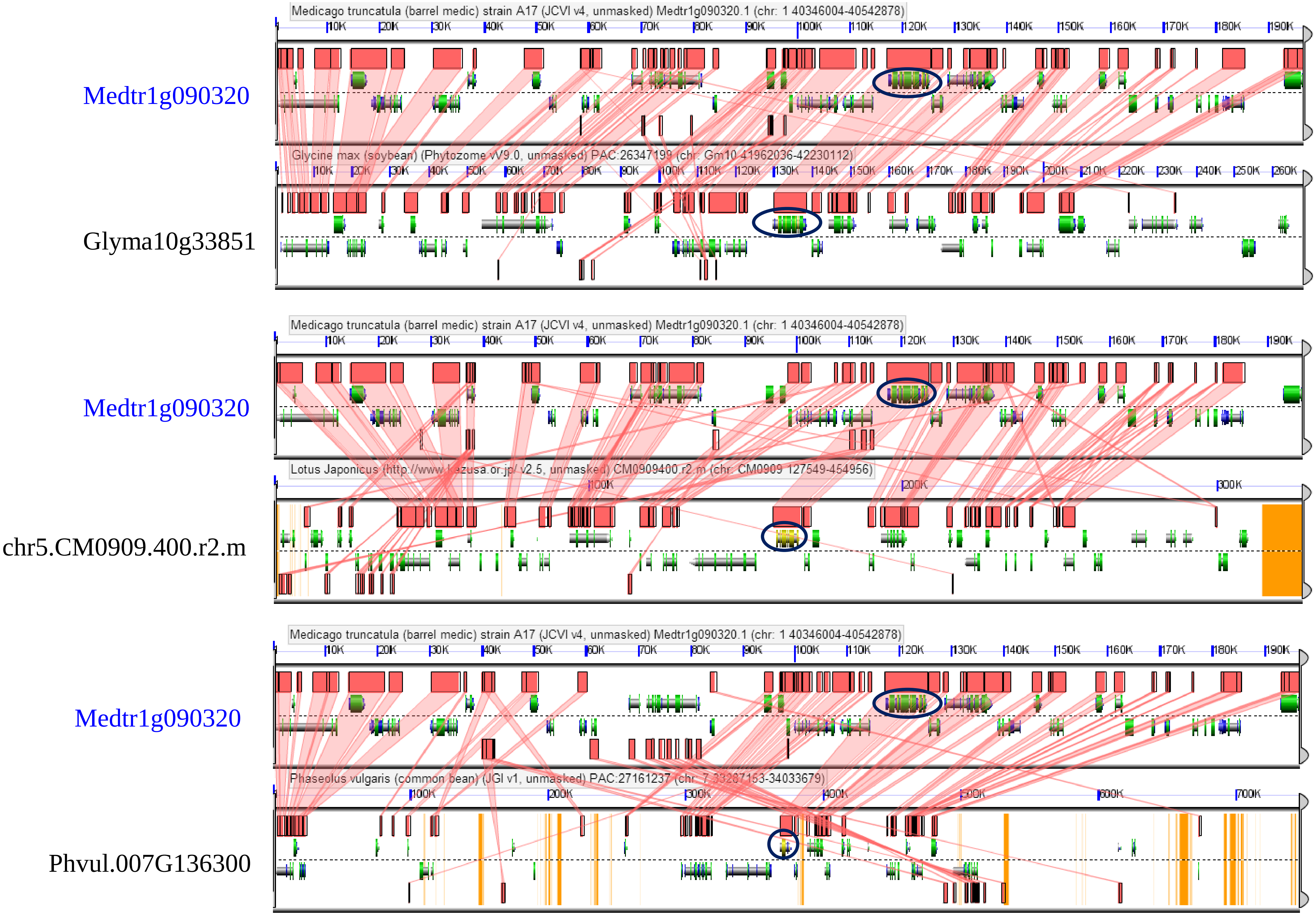

MtCRE1 orthologous genes in genomic regions of four legume plants

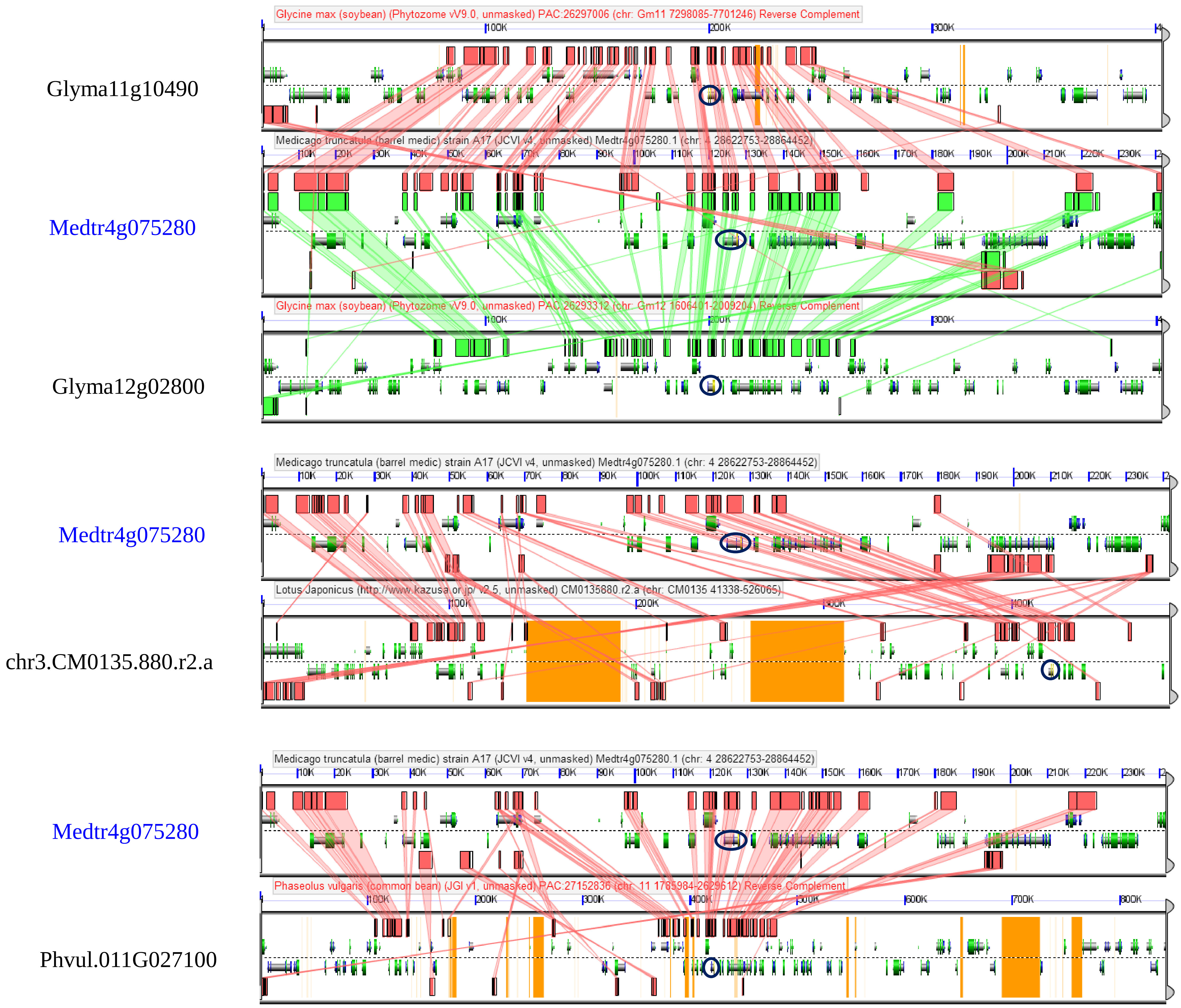

MtPIN2 orthologous genes in genomic regions of four legume plants

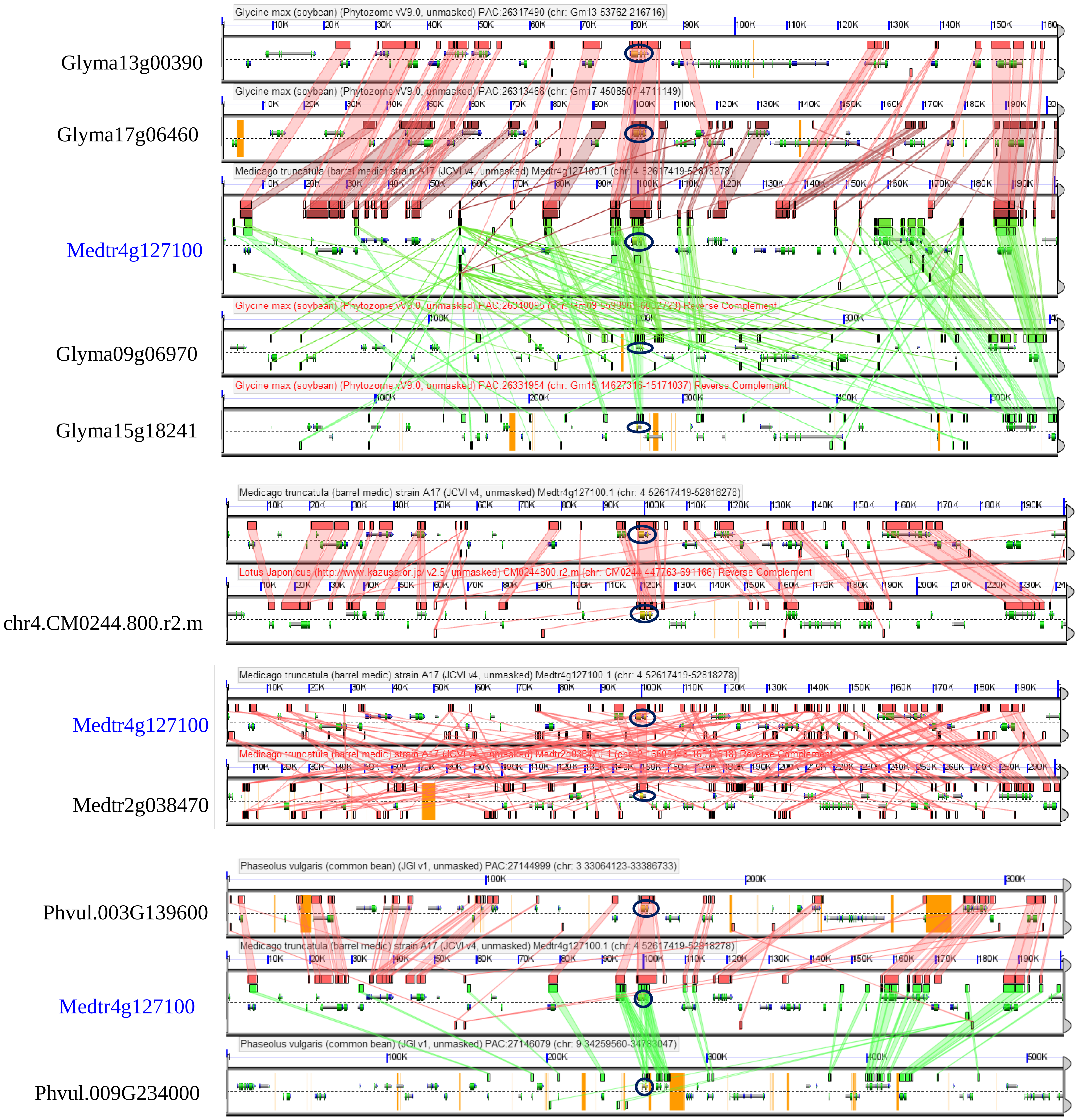

RIP1 orthologous genes in genomic regions of four legume plants

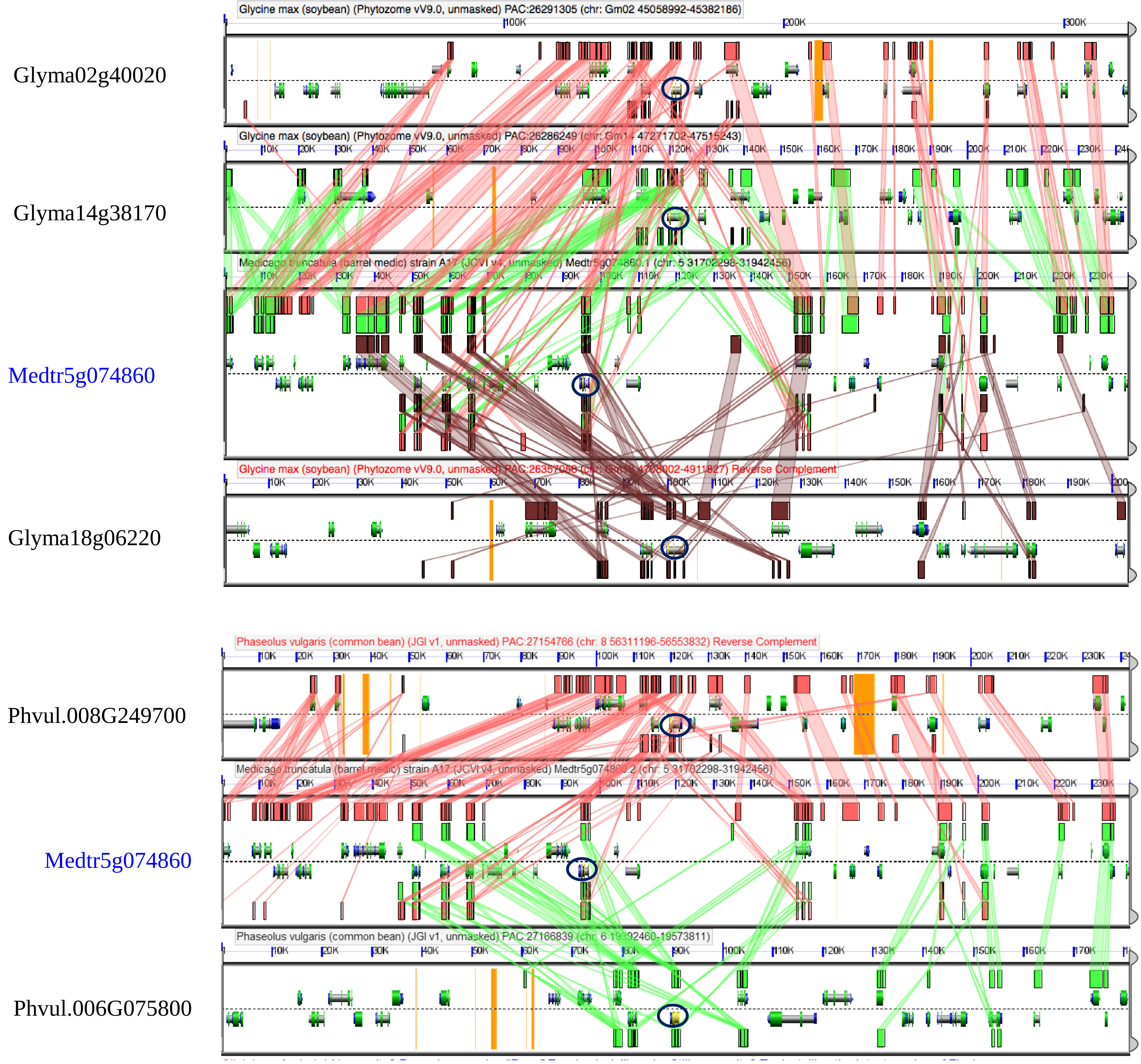

N56 orthologous genes in genomic regions of four legume plants

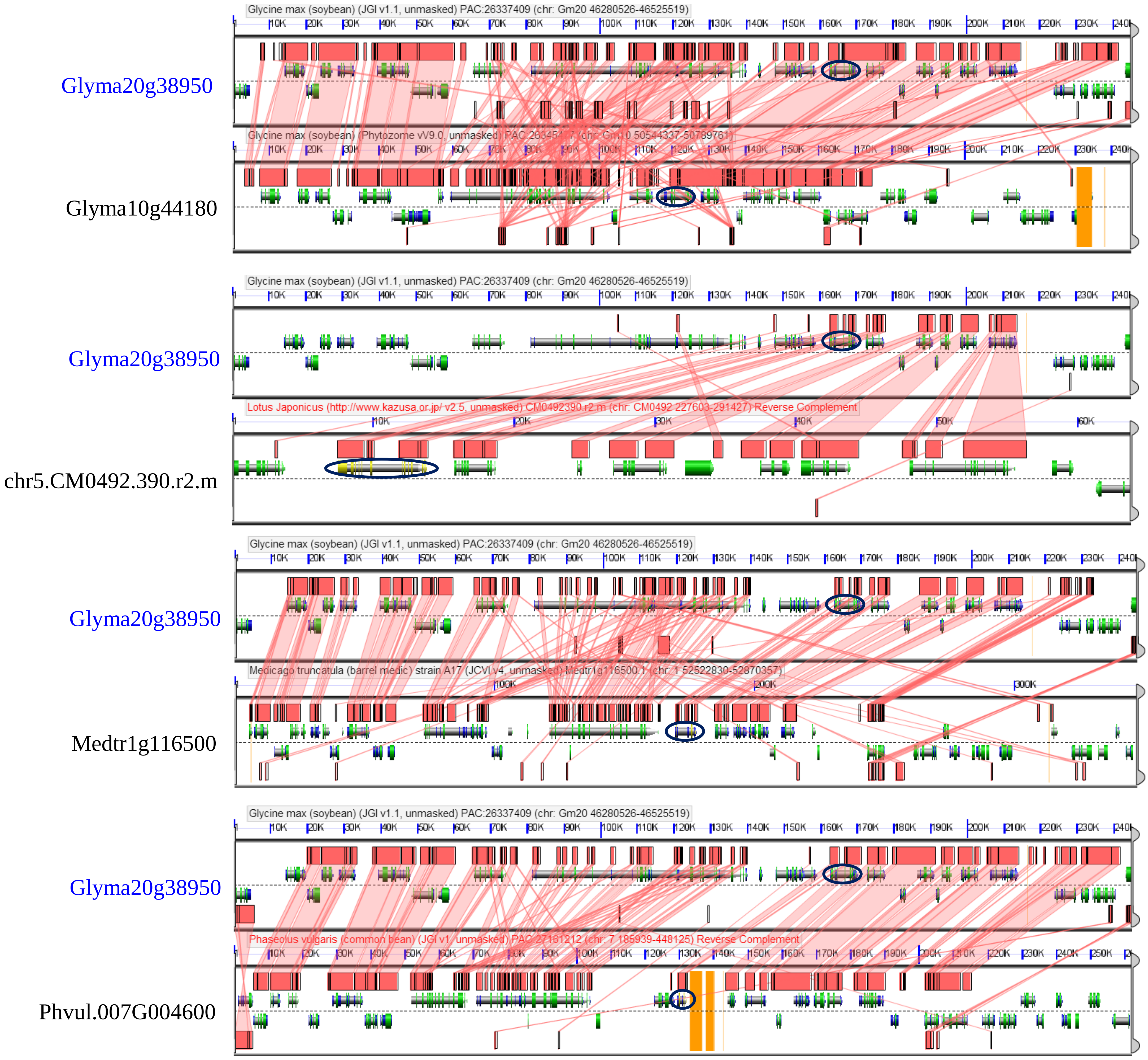

N70 orthologous genes in genomic regions of four legume plants

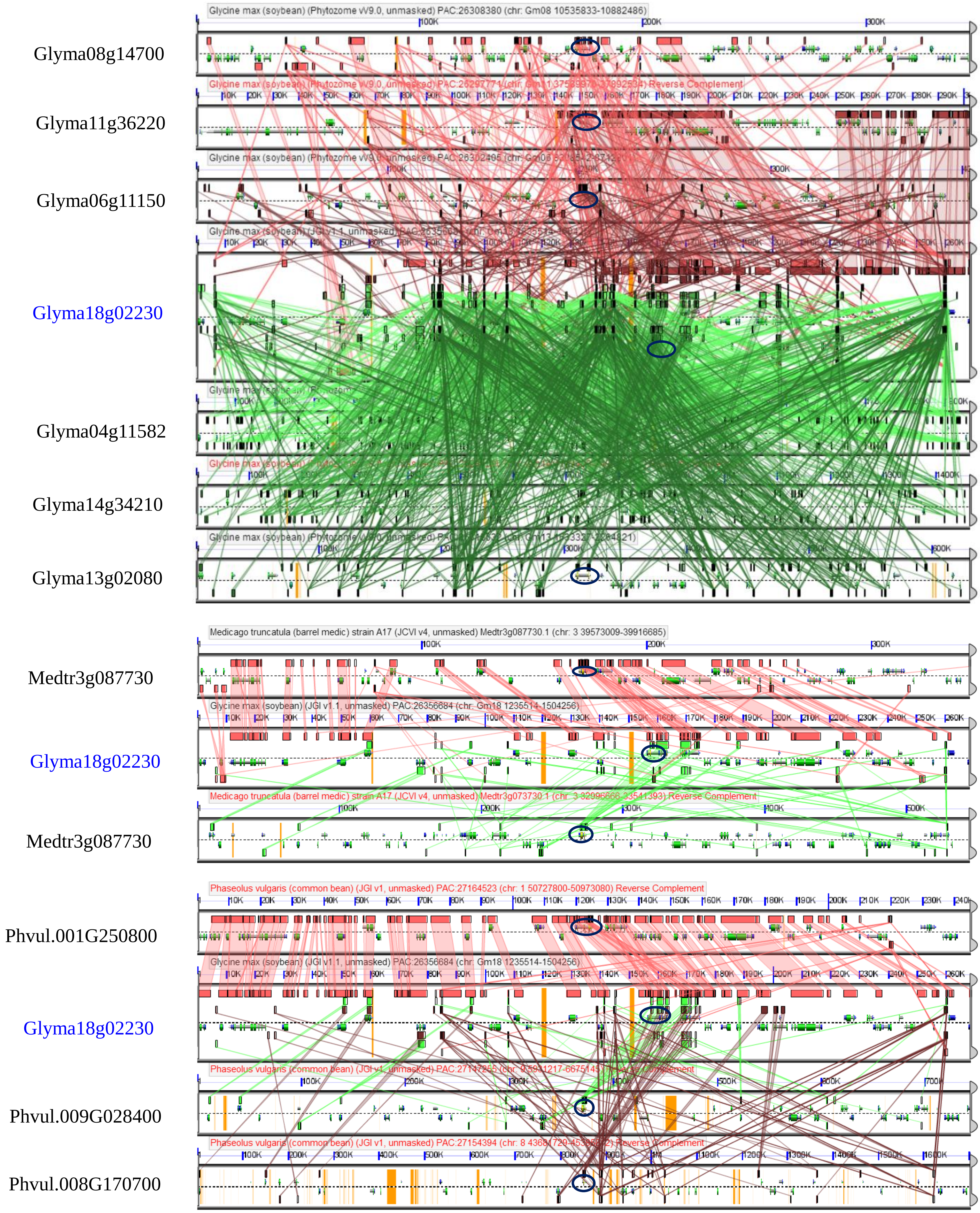

N93 orthologous genes in genomic regions of four legume plants

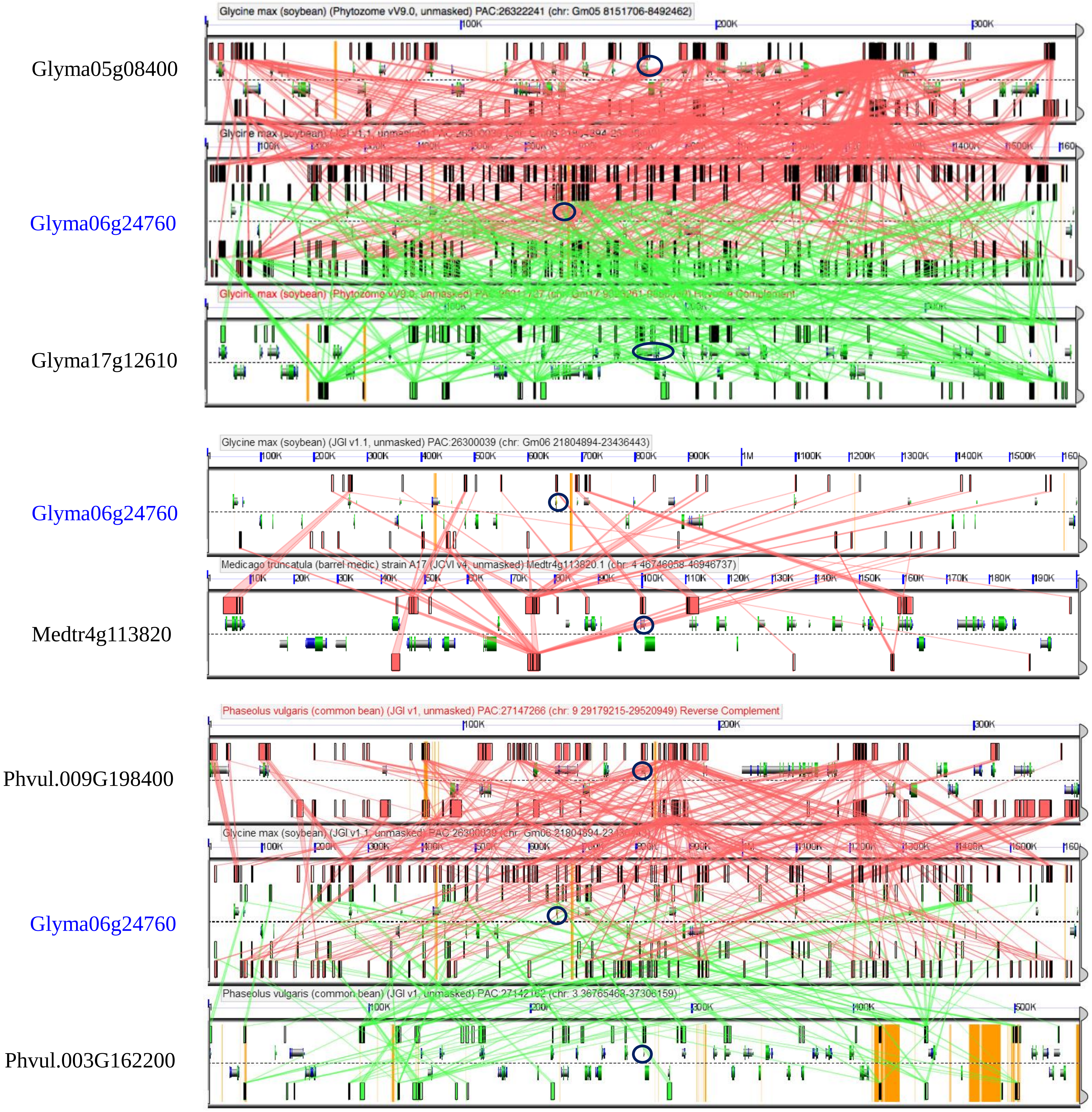

NODULIN-26a orthologous genes in genomic regions of four legume plants

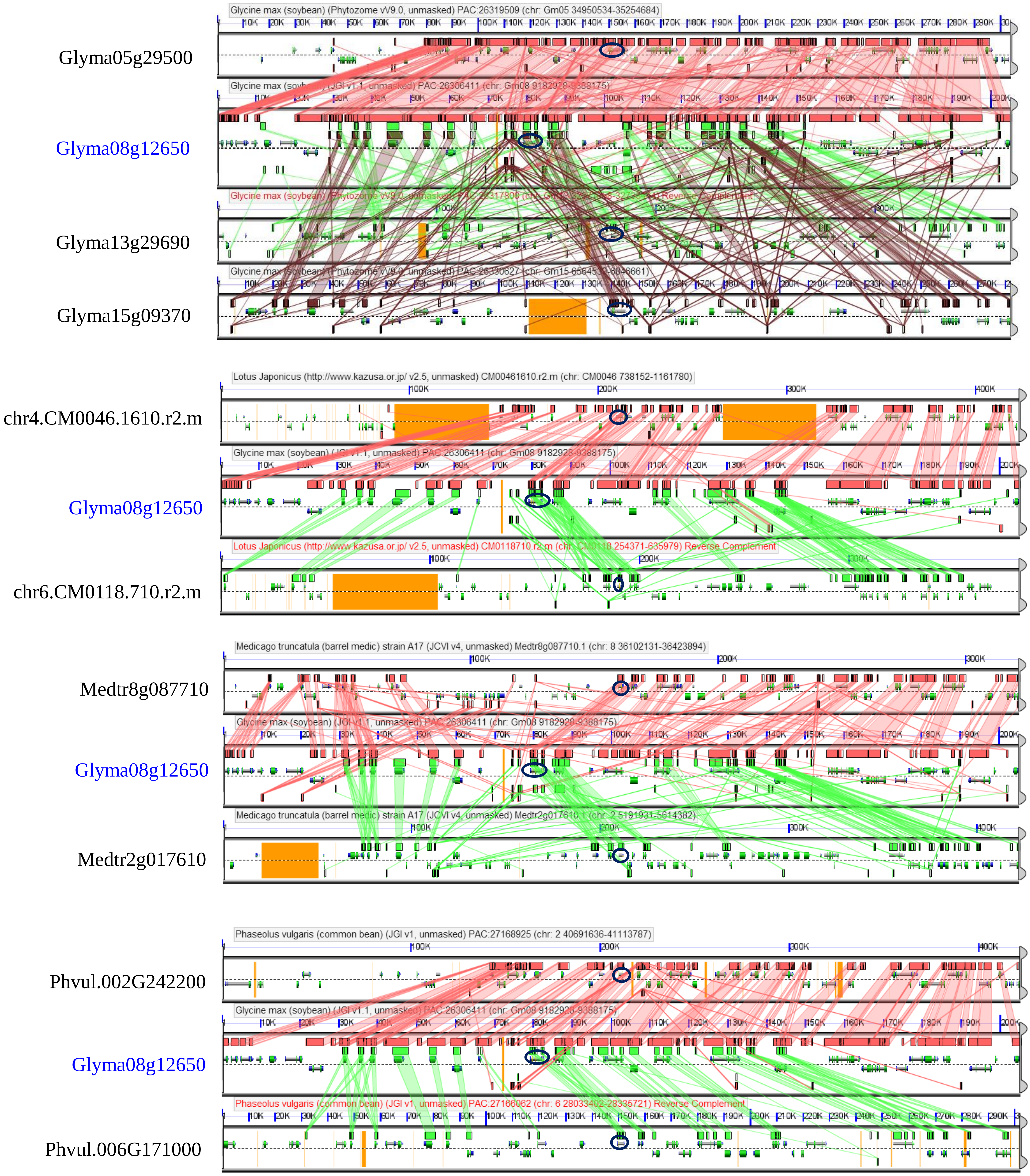

NODULIN-33 orthologous genes in genomic regions of four legume plants

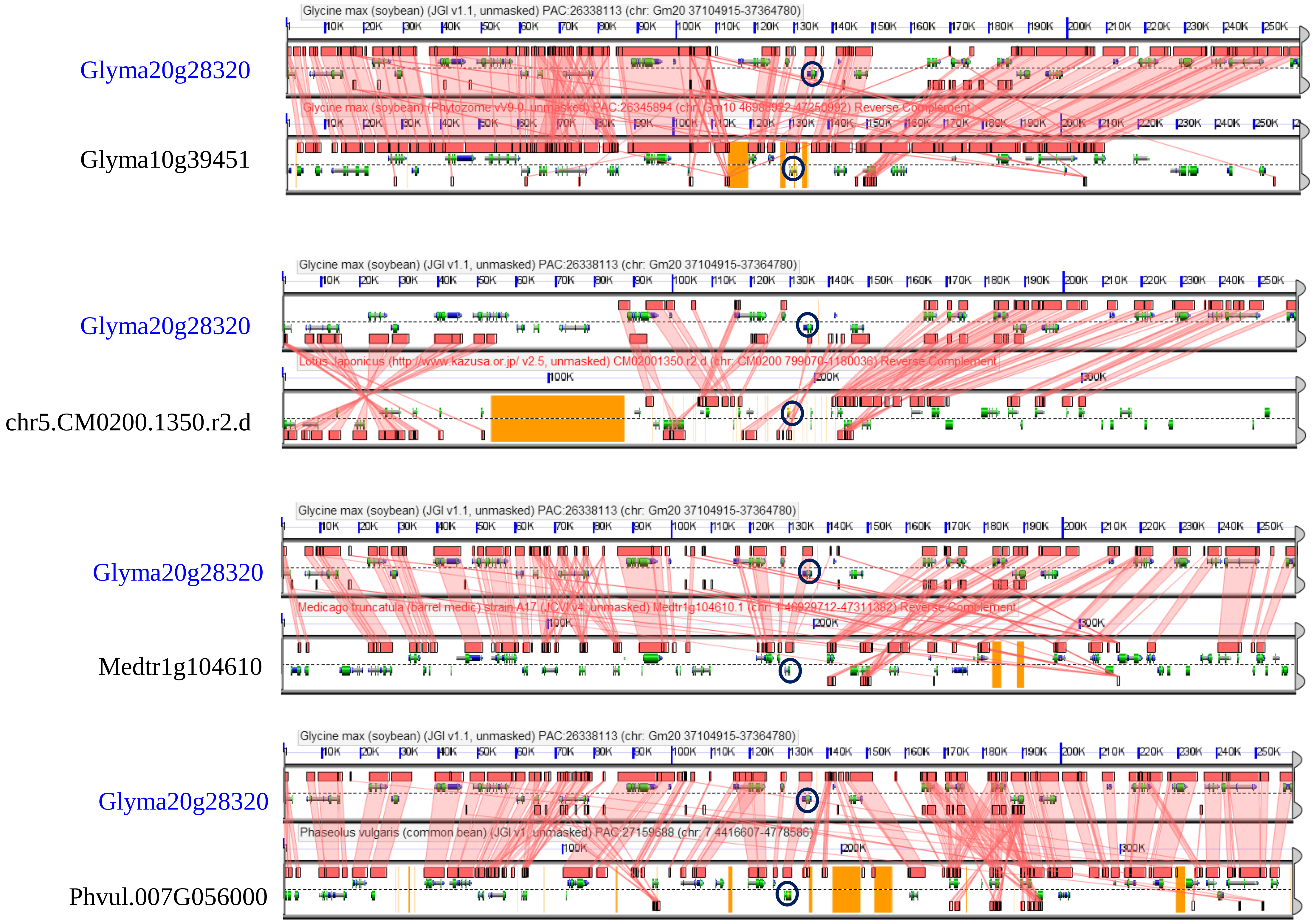

MtAnn1 orthologous genes in genomic regions of four legume plants

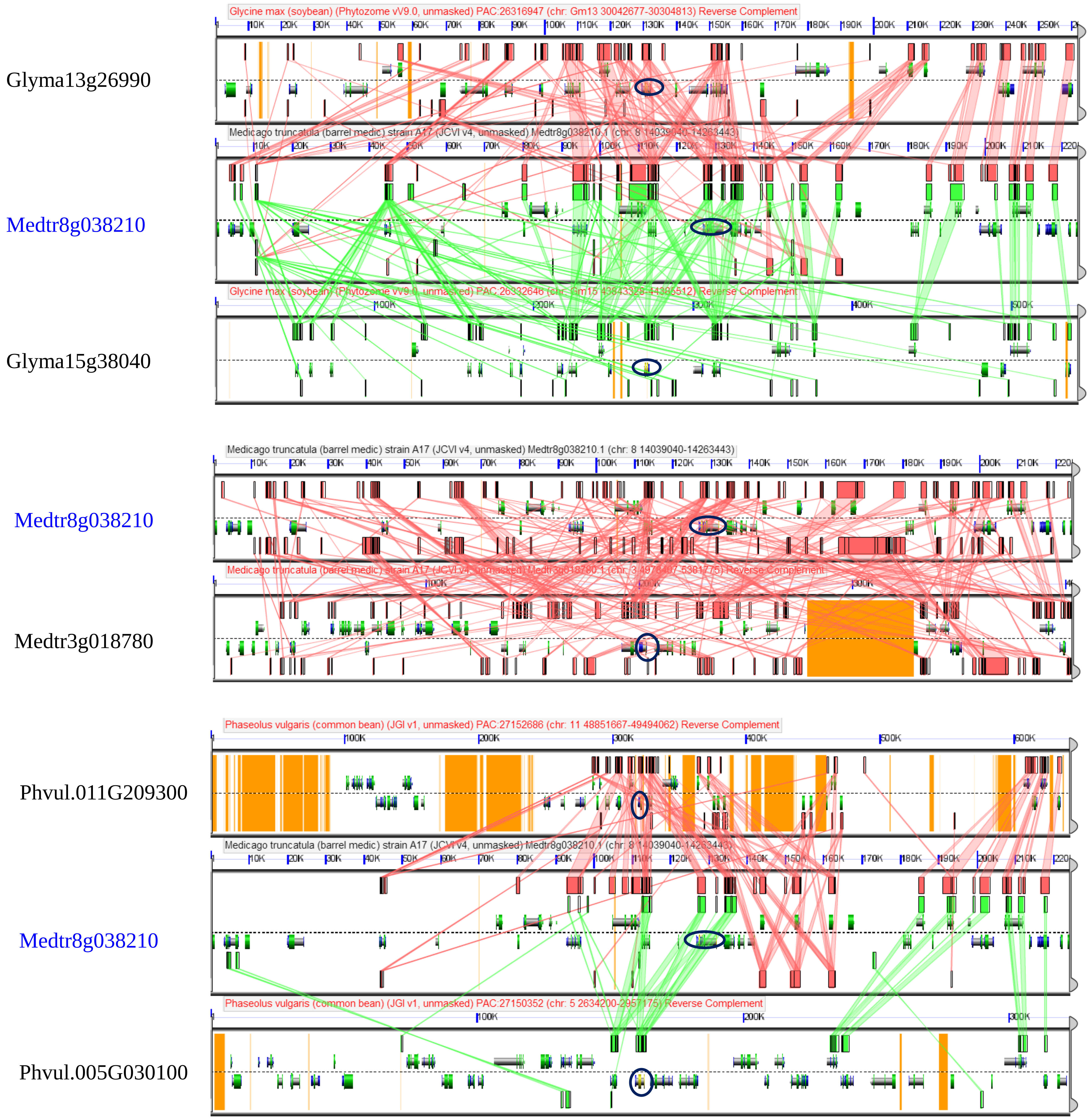

MtAUX1-like orthologous genes in genomic regions of four legume plants

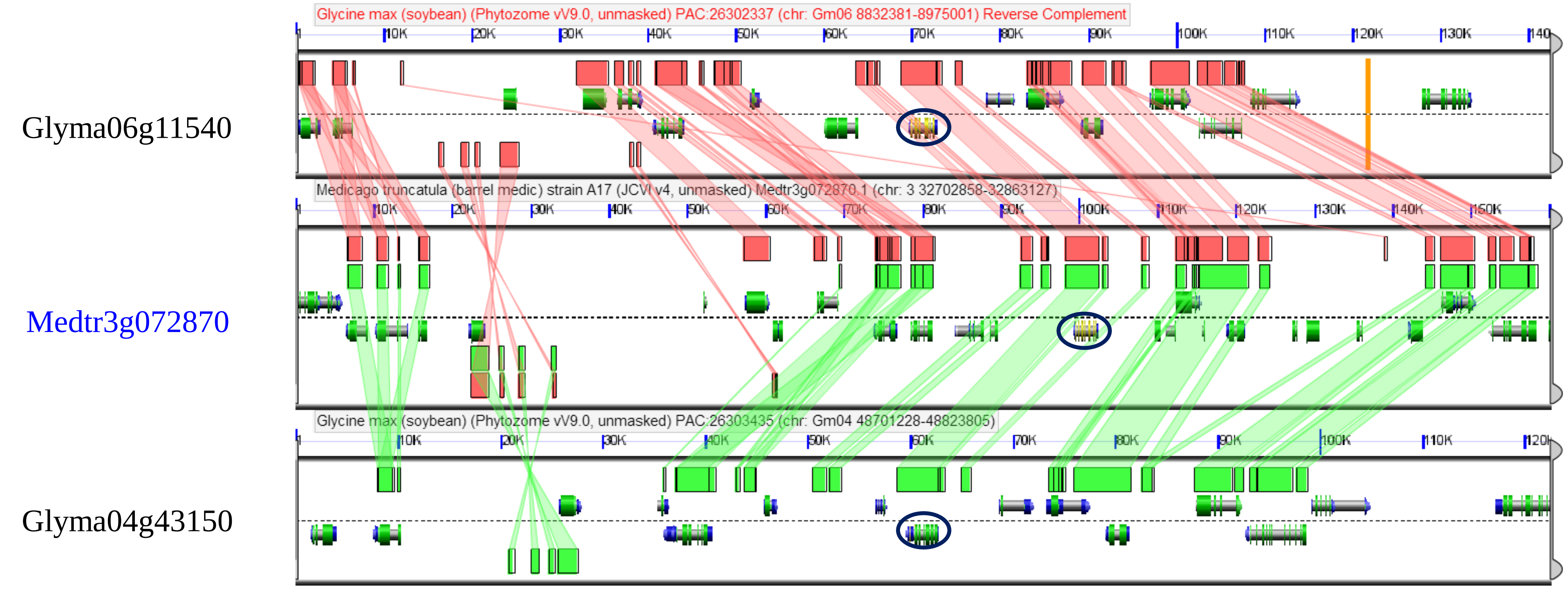

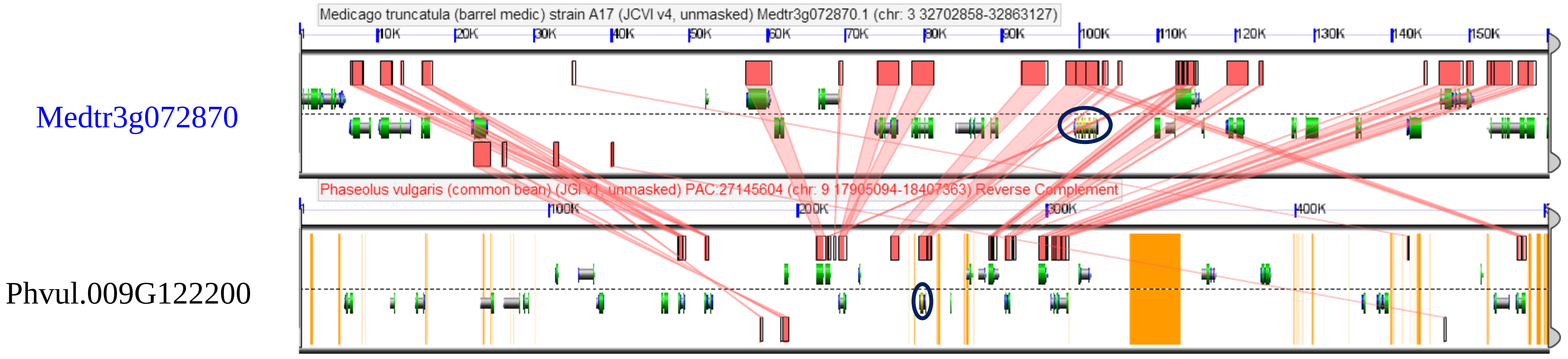

LjSbtS orthologous genes in genomic regions of four legume plants

Glyma11g19130

Medtr4g053630

Glyma12g09290

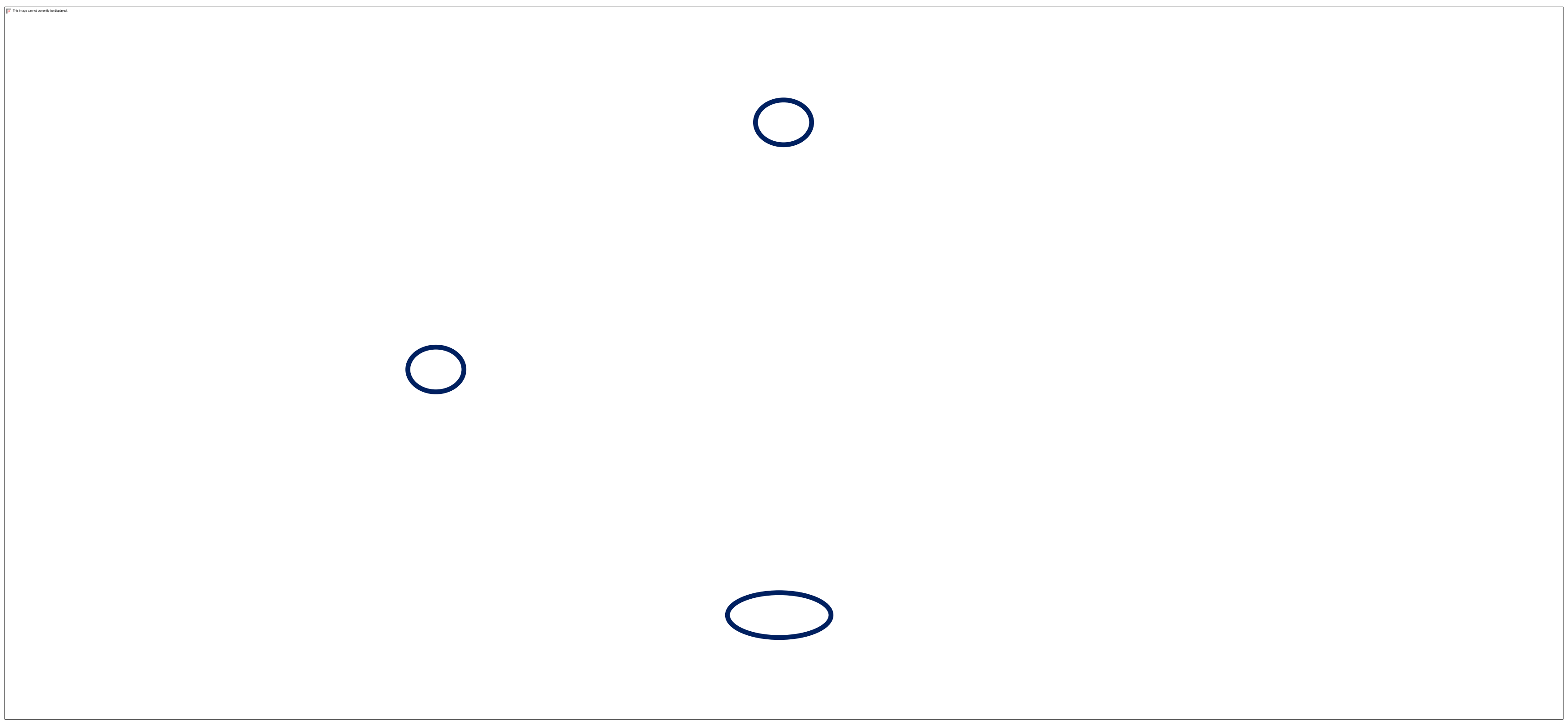

Medtr4g053630

chr3.CM1144.130.r2.m

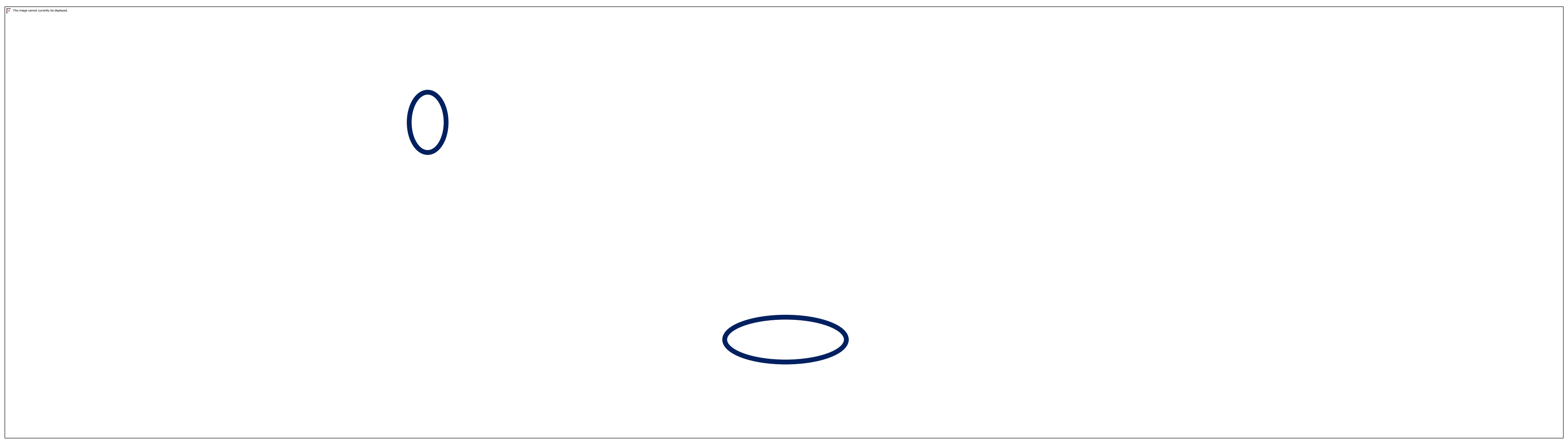

Medtr4g053630

Medtr5g055920

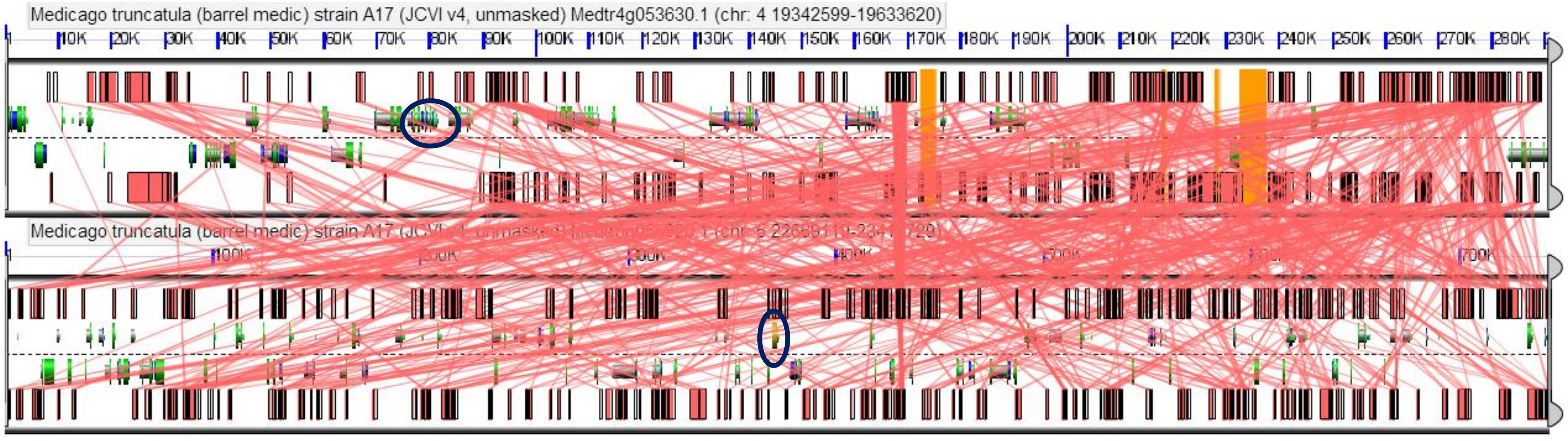

Medtr4g053630

Phvul.011G092600

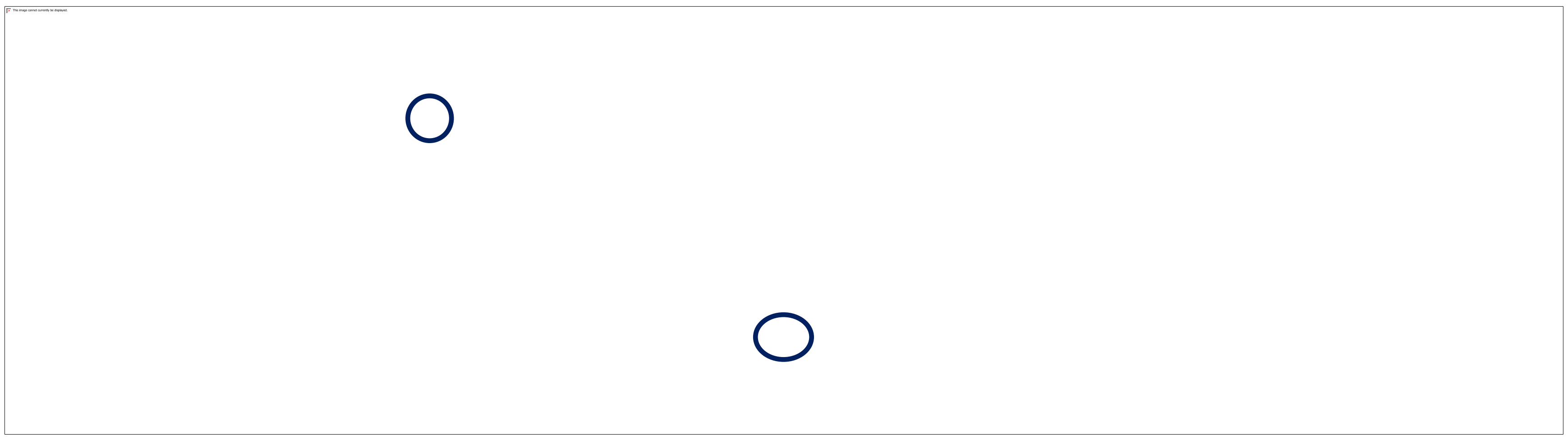

LjCysS orthologous genes in genomic regions of four legume plants

Glyma11g20400

Glyma12g08200

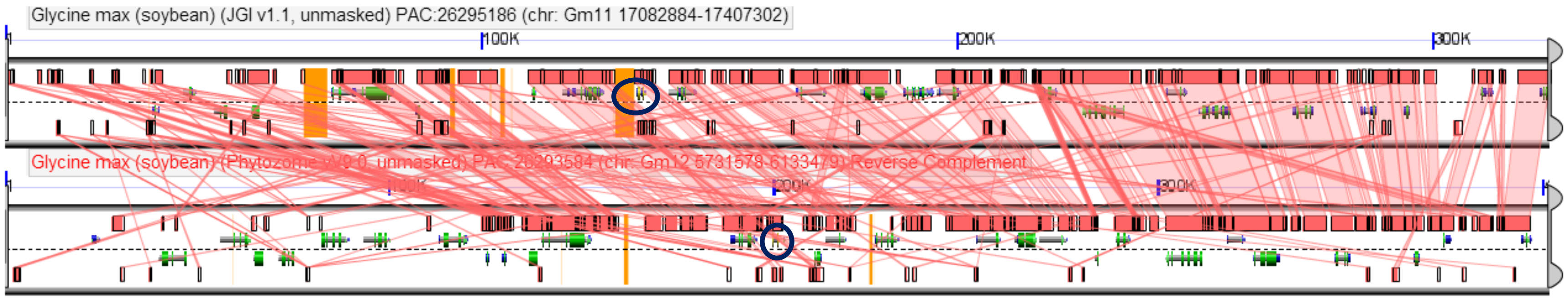

Glyma11g20400

chr3.CM0649.310.r2.d

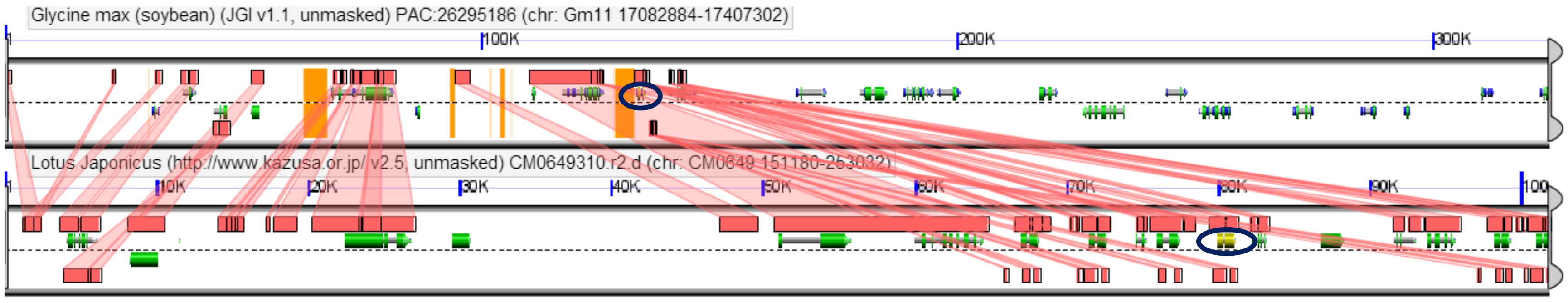

Glyma11g20400

Phvul.011G081100

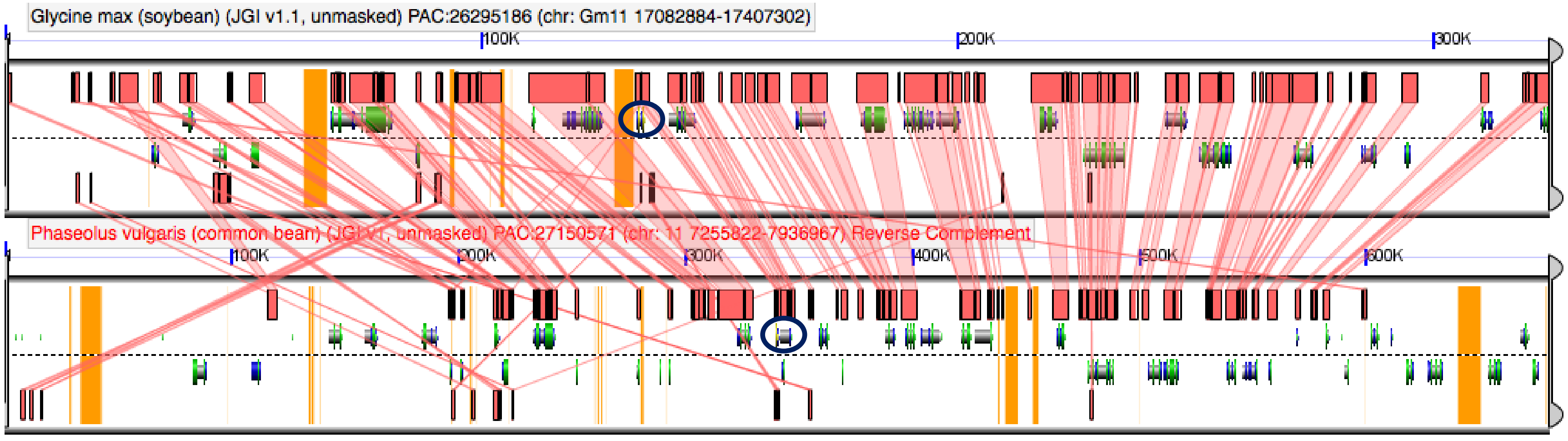

MtROP10 orthologous genes in genomic regions of four legume plants

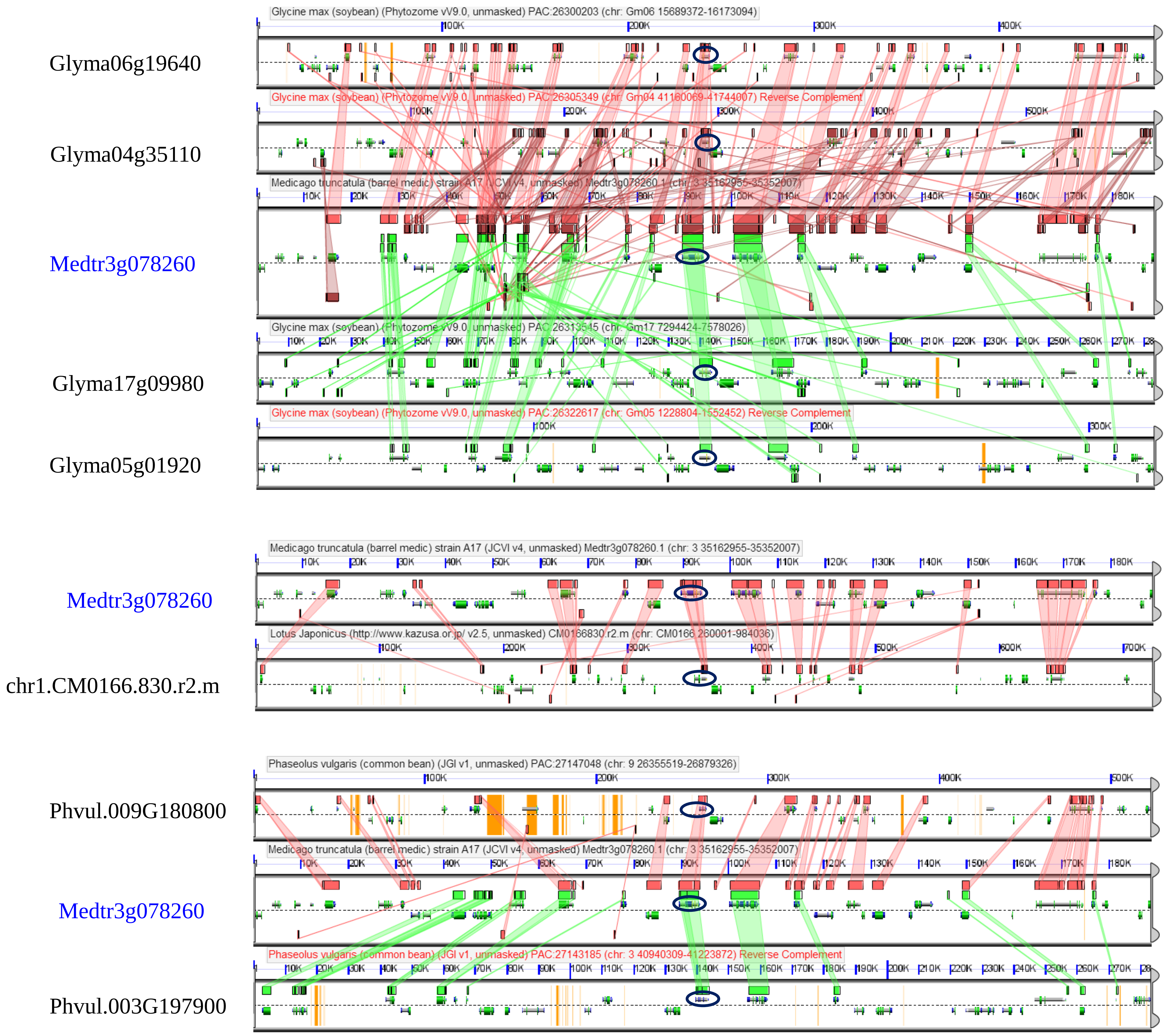

MtPUB1 orthologous genes in genomic regions of four legume plants

Medtr5g083030

Glyma02g43190

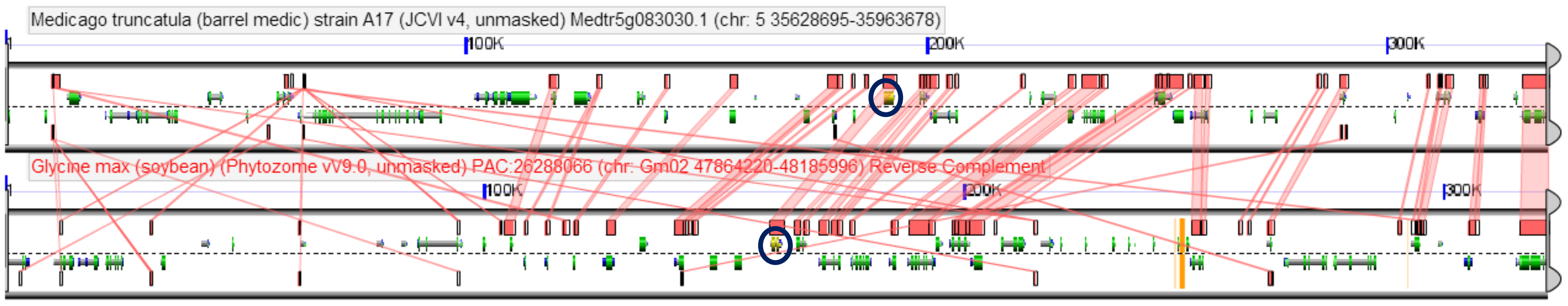

chr2.CM0002.630.r2.m

Medtr5g083030

chr6.LjB02K20.60.r2.m

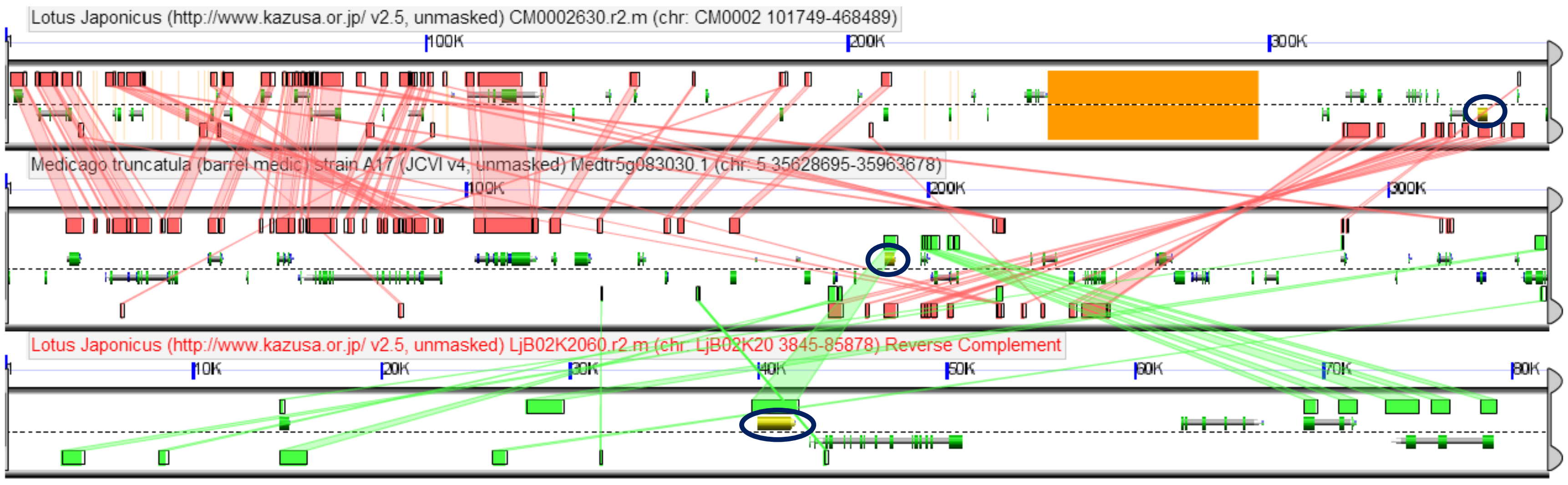

Medtr5g083030

Medtr3g071425

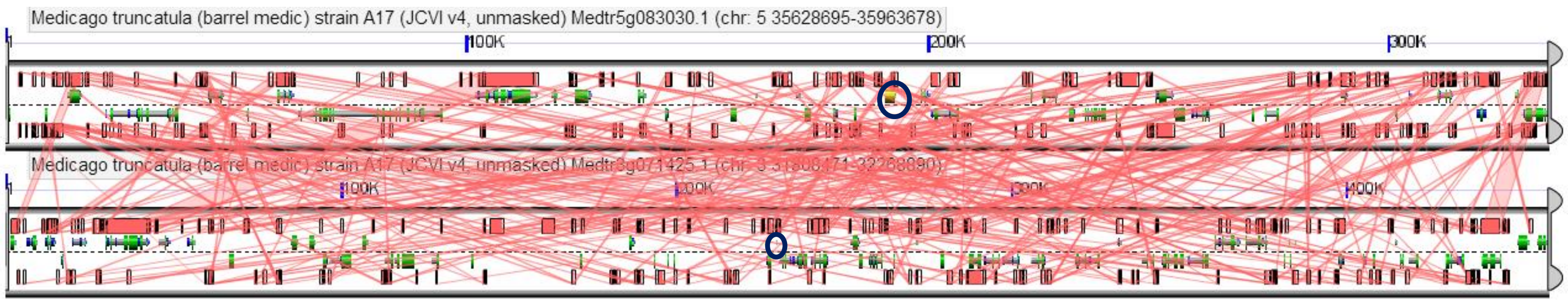

Medtr5g083030

Phvul.008G222100

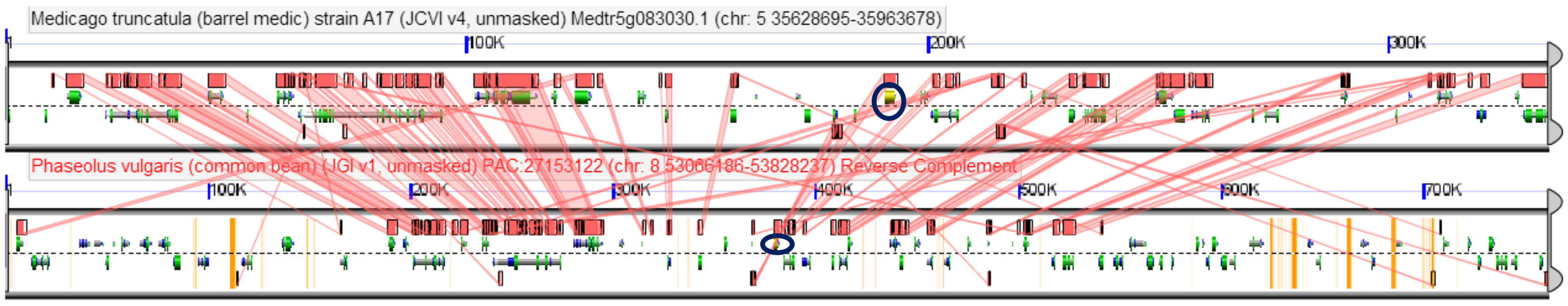

FLOT2/4 orthologous genes in genomic regions of four legume plants

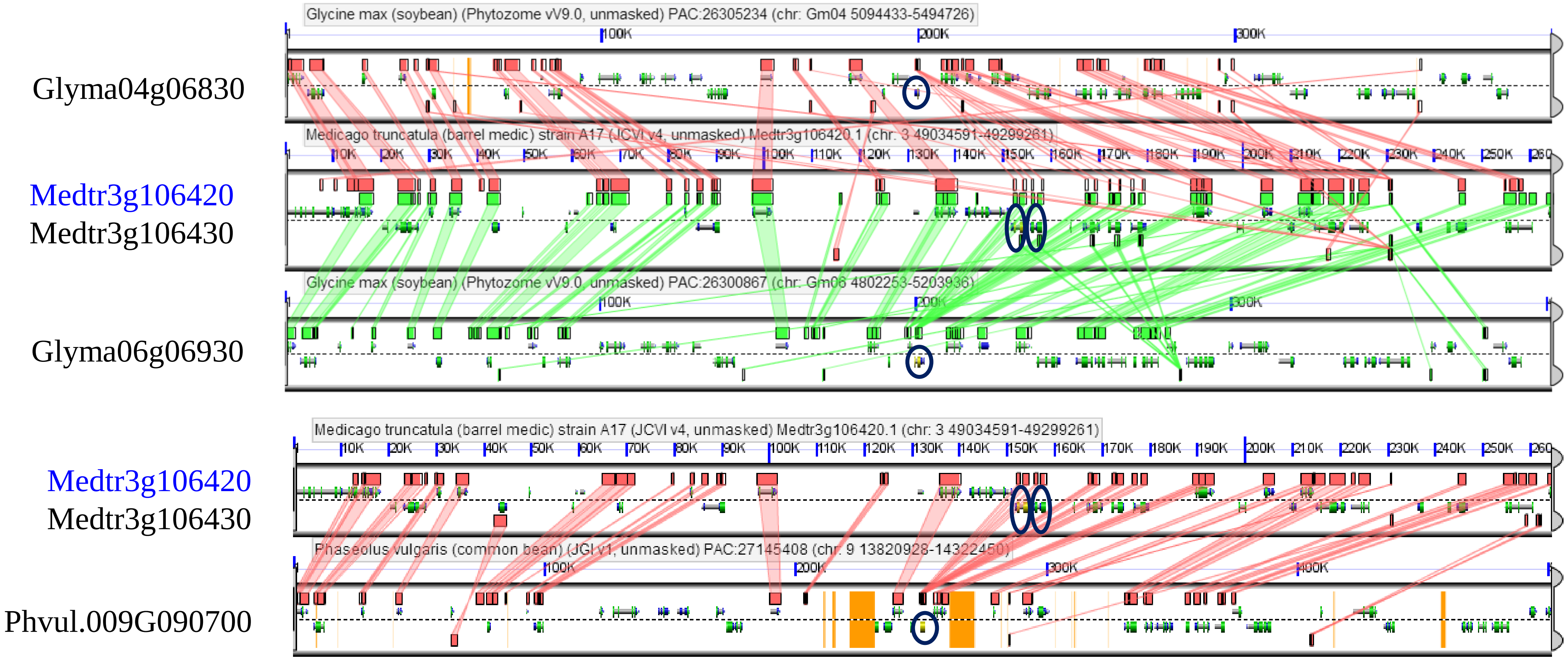

RDN1 orthologous genes in genomic regions of four legume plants

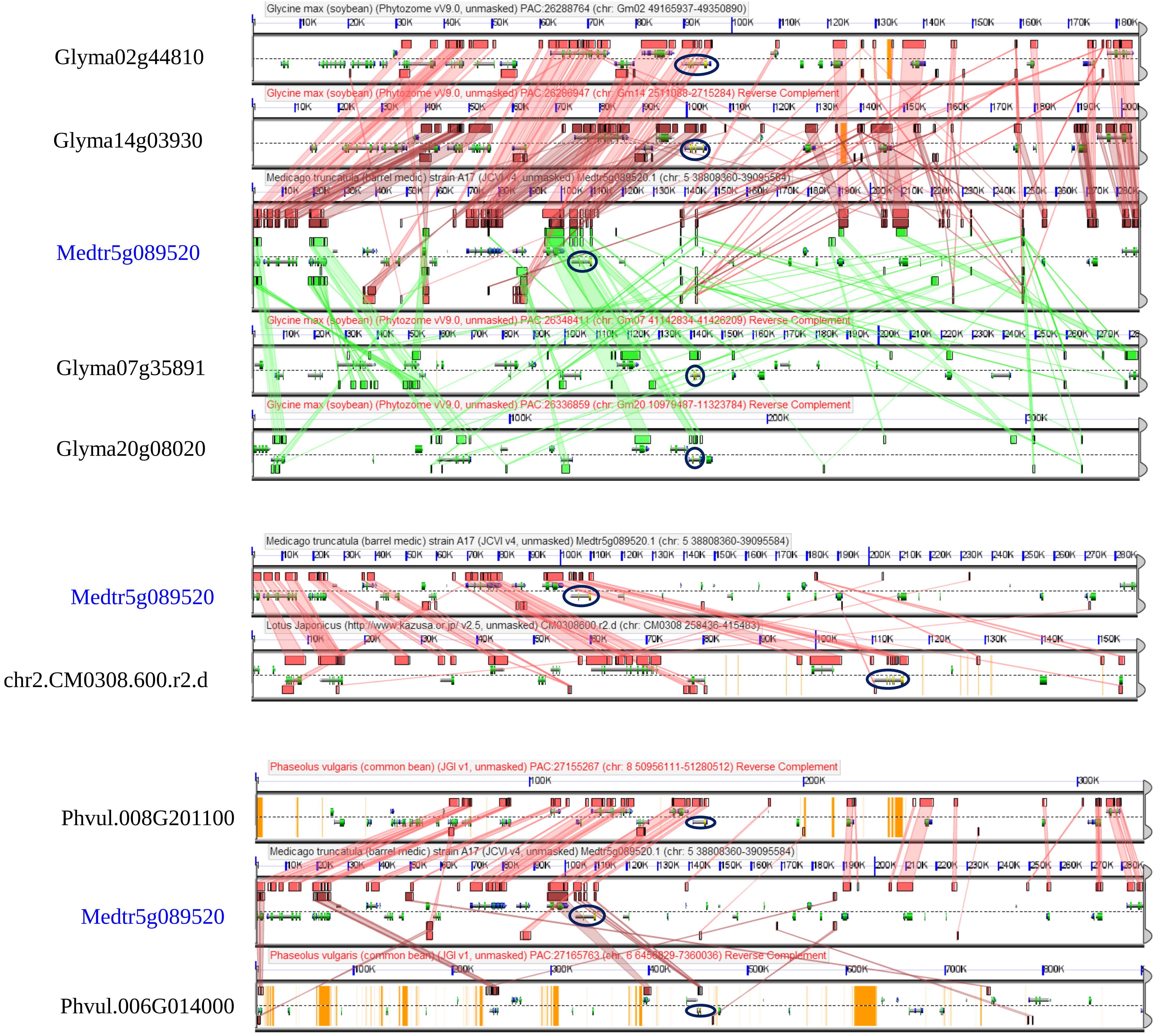

RDN2 orthologous genes in genomic regions of four legume plants

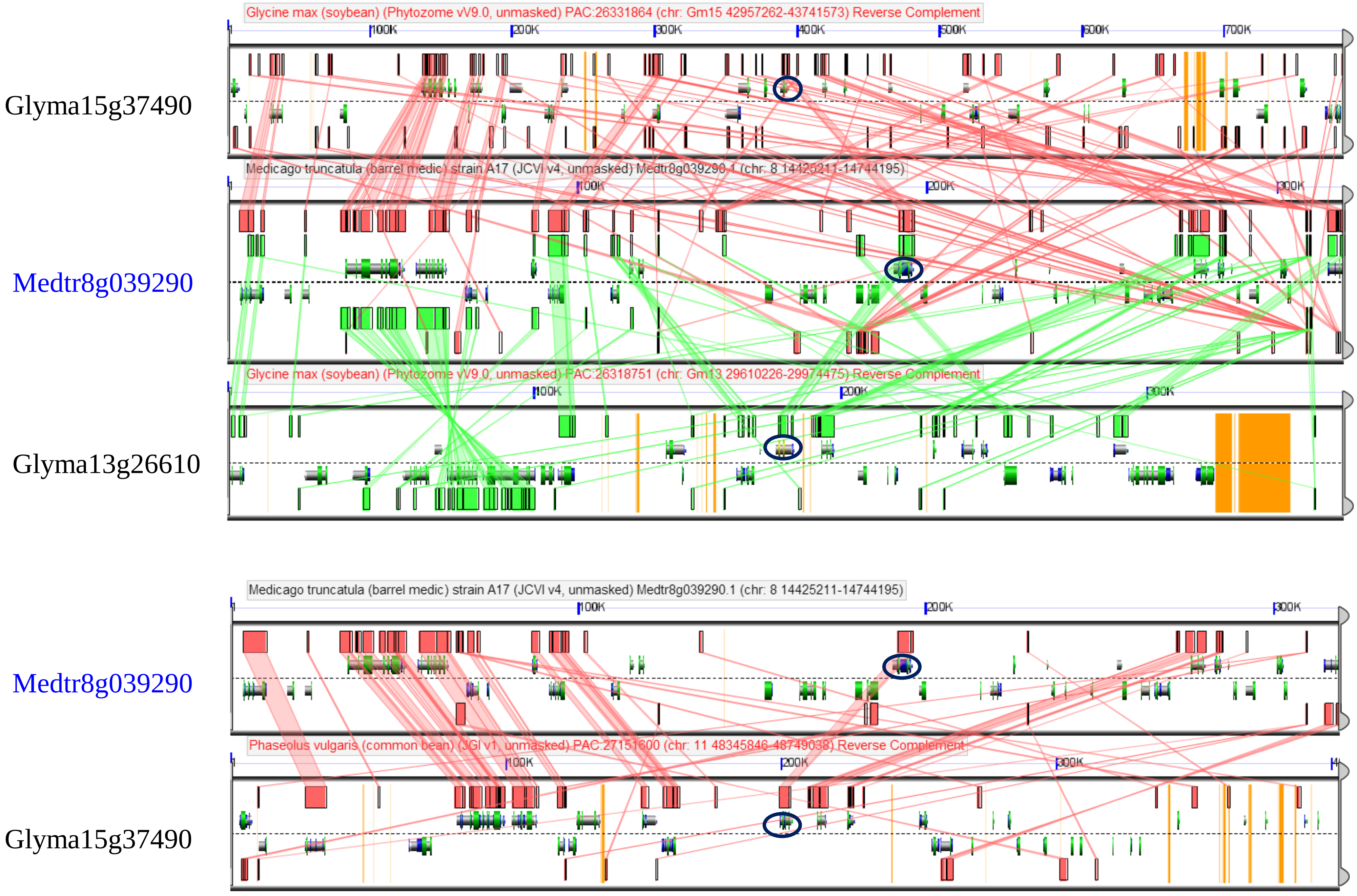

RDN3A orthologous genes in genomic regions of four legume plants

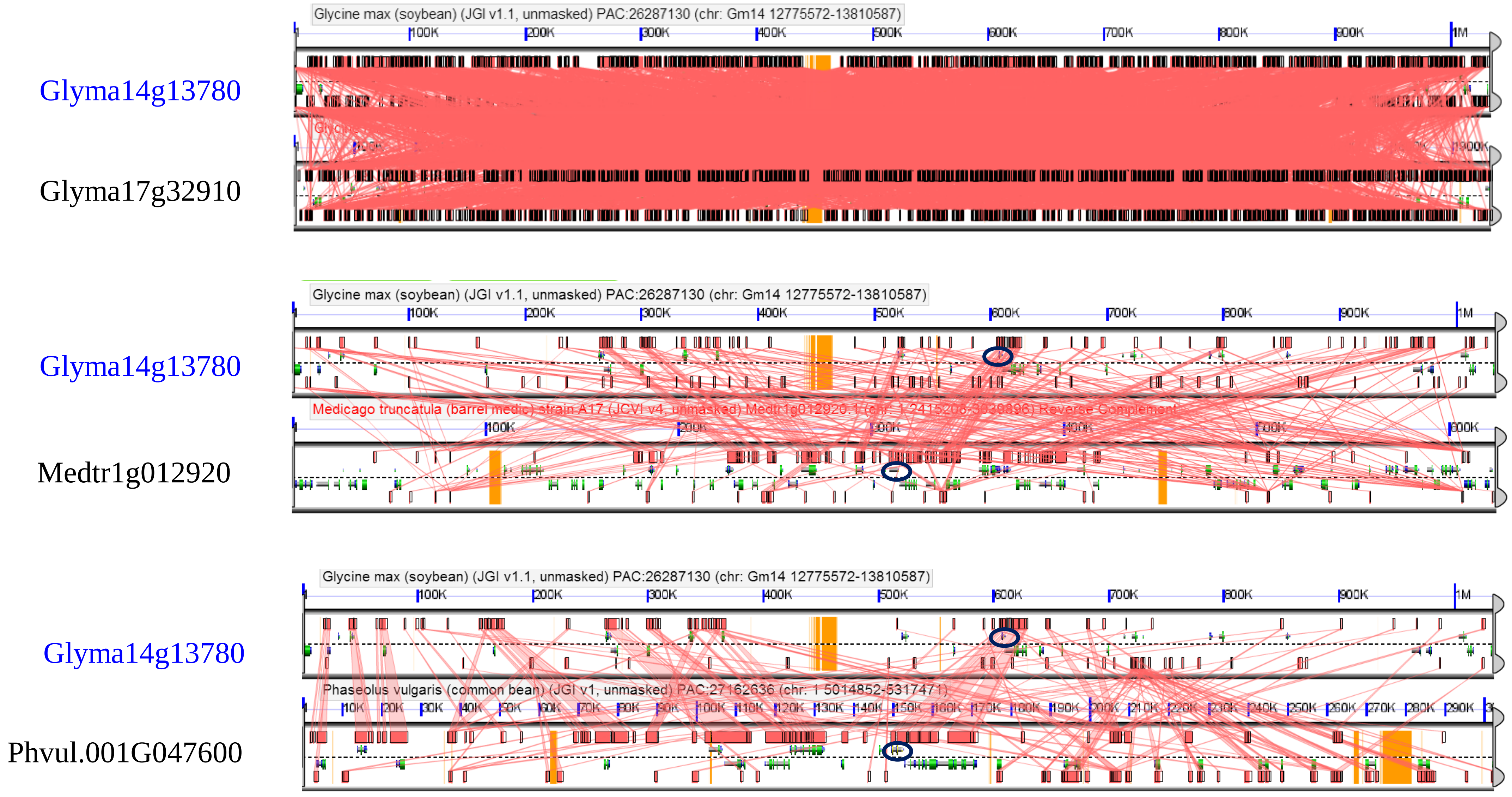

NMNa orthologous genes in genomic regions of four legume plants

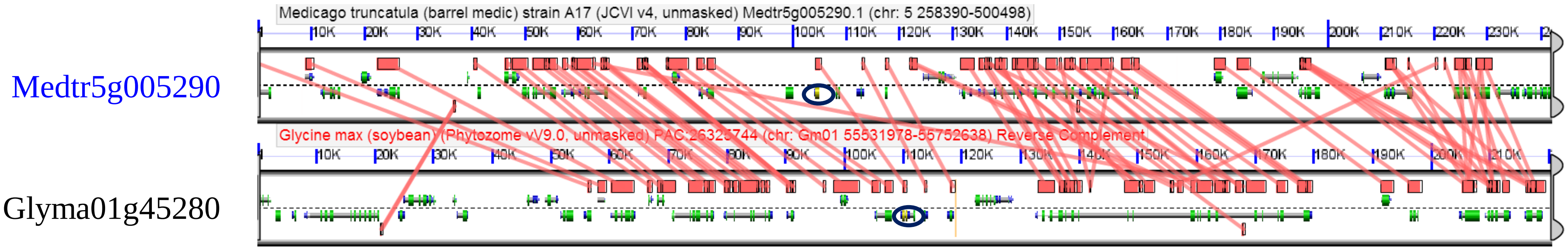

LjCHC1 orthologous genes in genomic regions of four legume plants

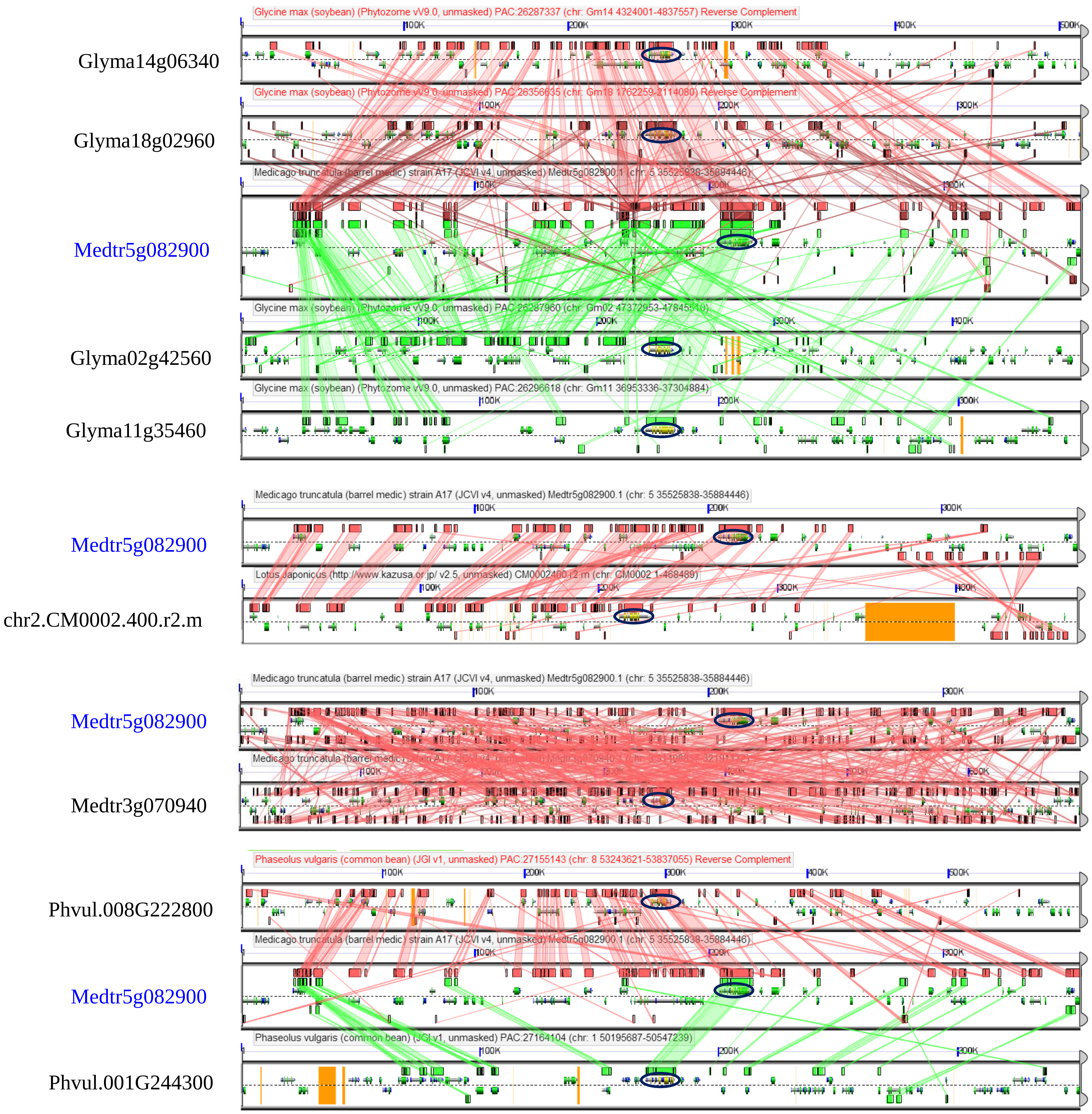

LjCHC2 orthologous genes in genomic regions of four legume plants

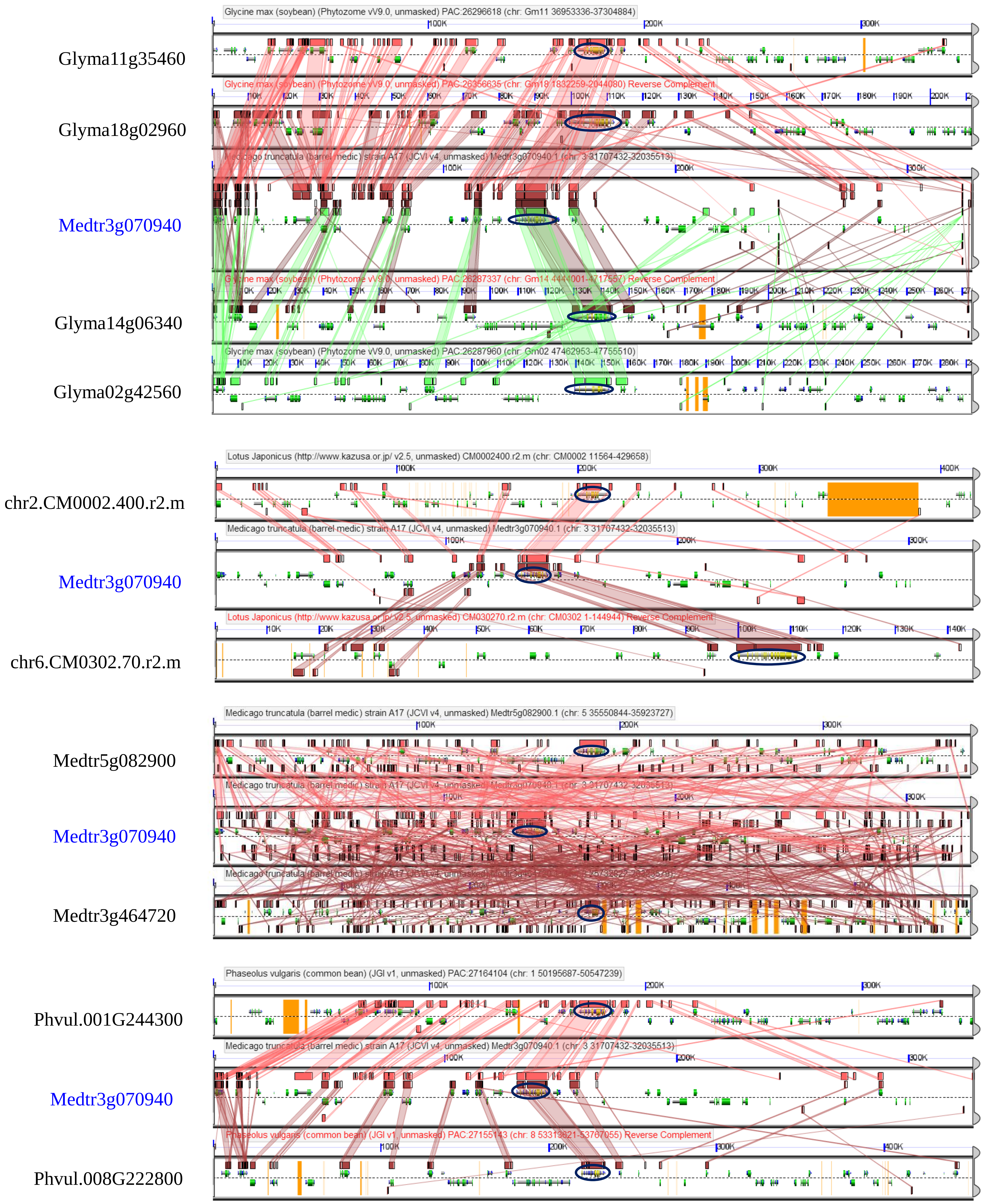

MtNramp1 orthologous genes in genomic regions of four legume plants

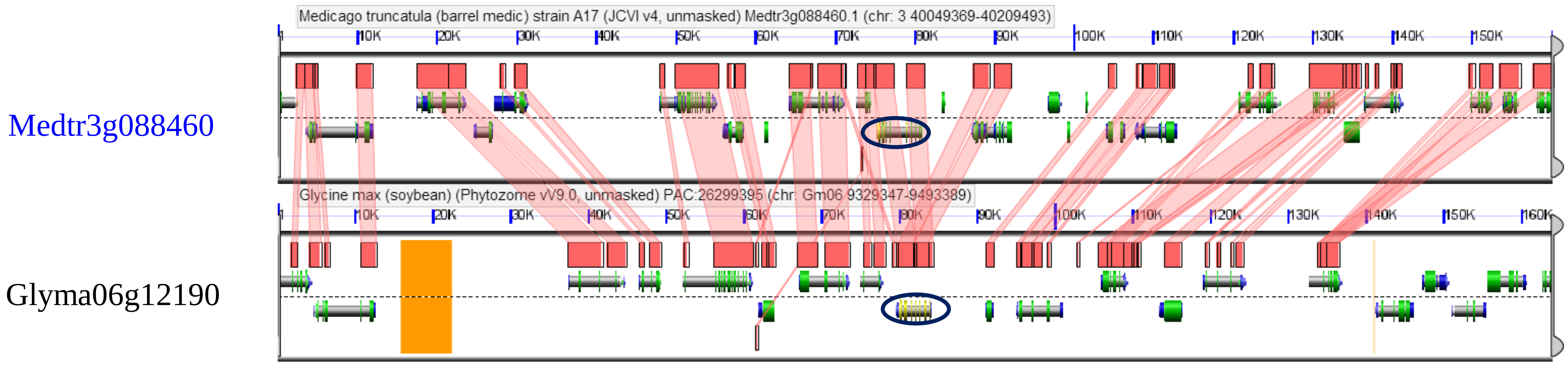

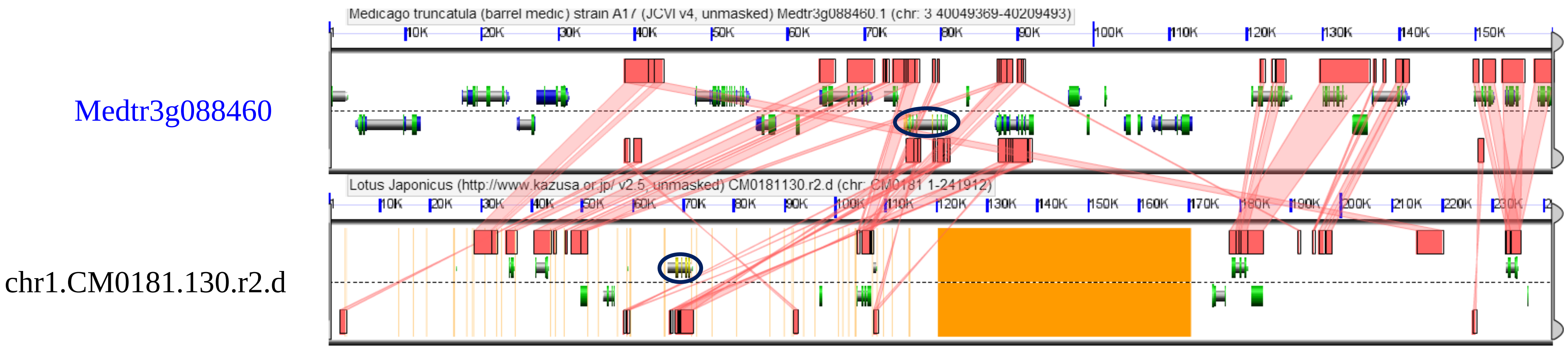

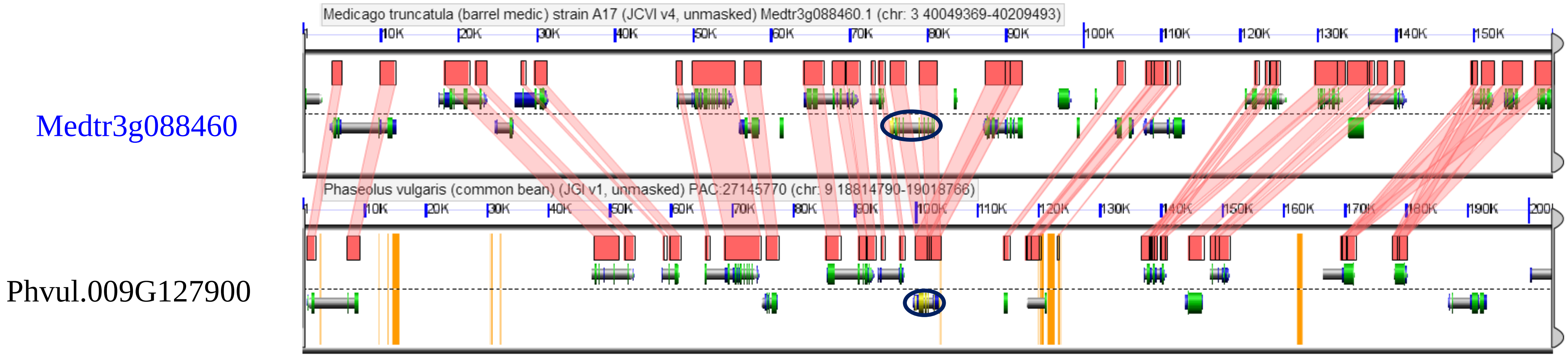

KAPP orthologous genes in genomic regions of four legume plants

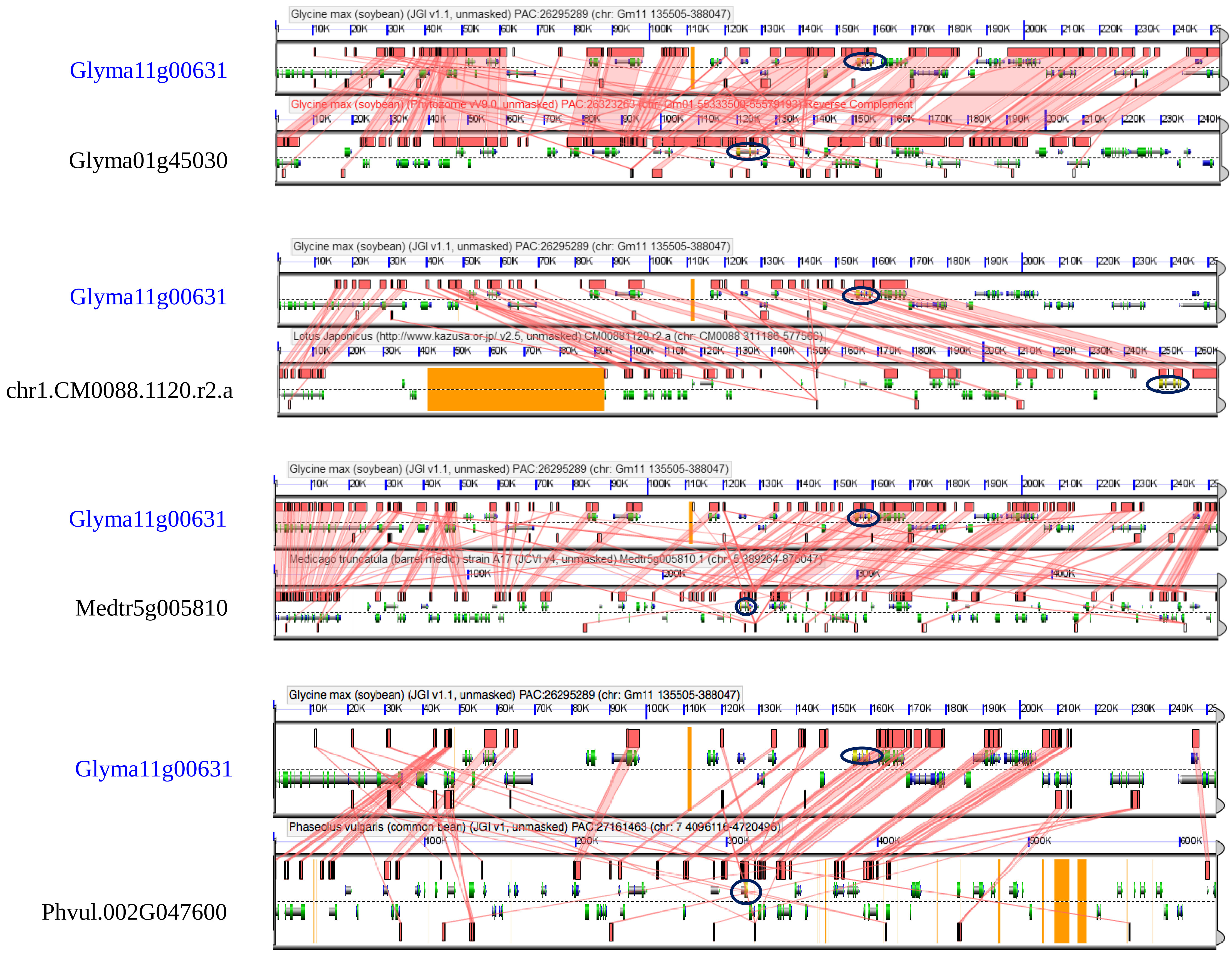

ROP9 orthologous genes in genomic regions of four legume plants

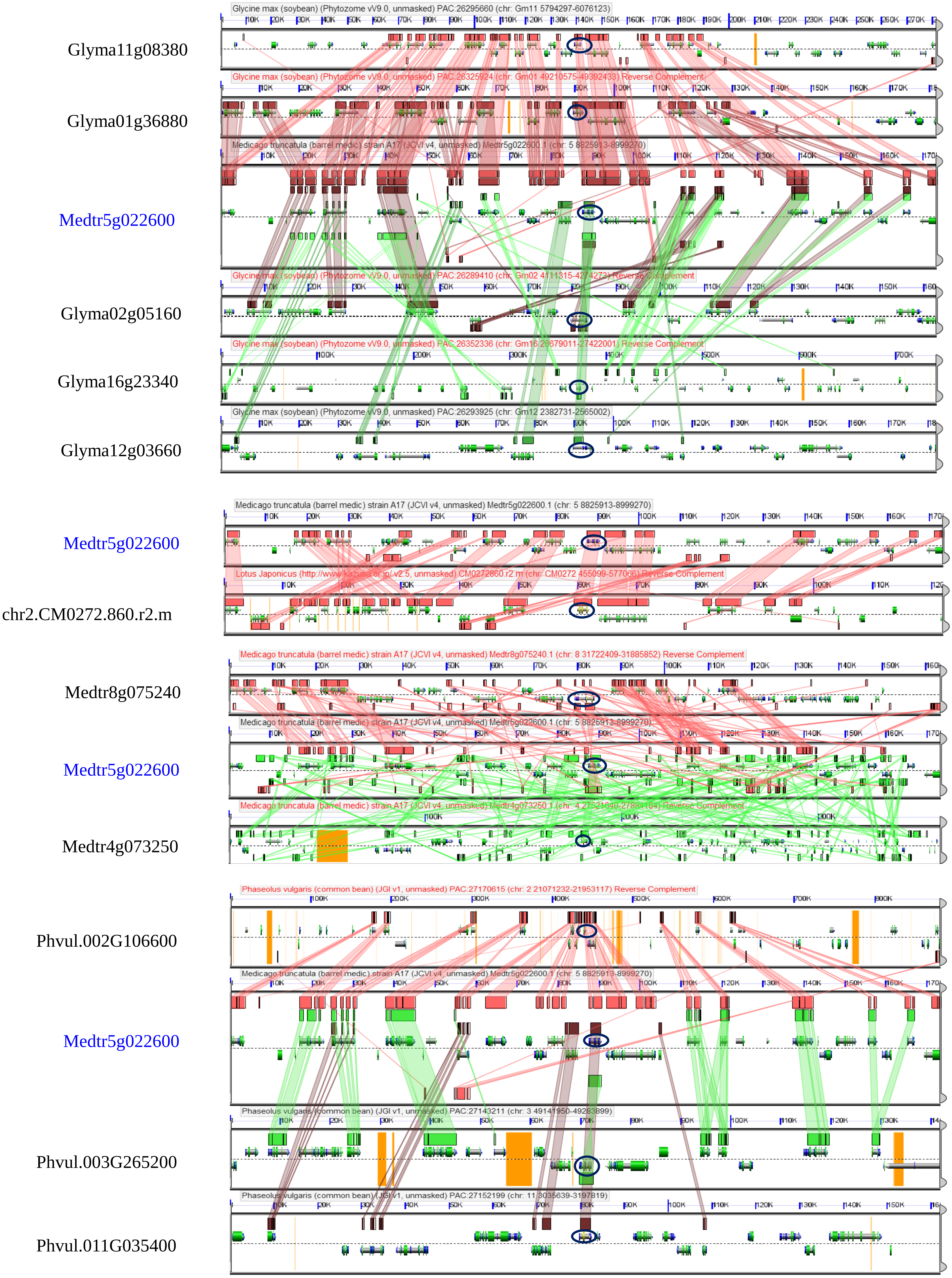

EPR3 orthologous genes in genomic regions of four legume plants

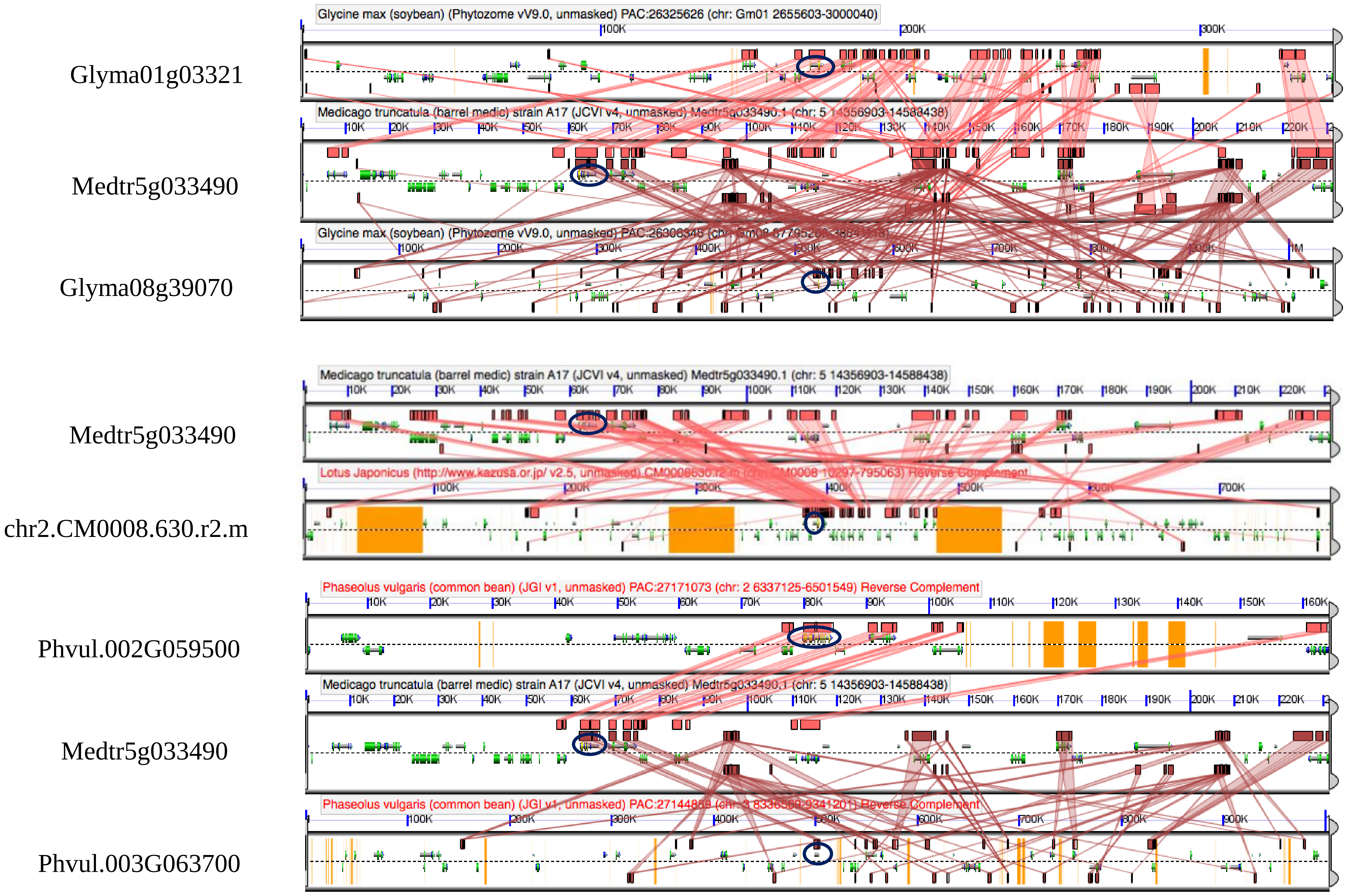

NNC1 orthologous genes in genomic regions of four legume plants

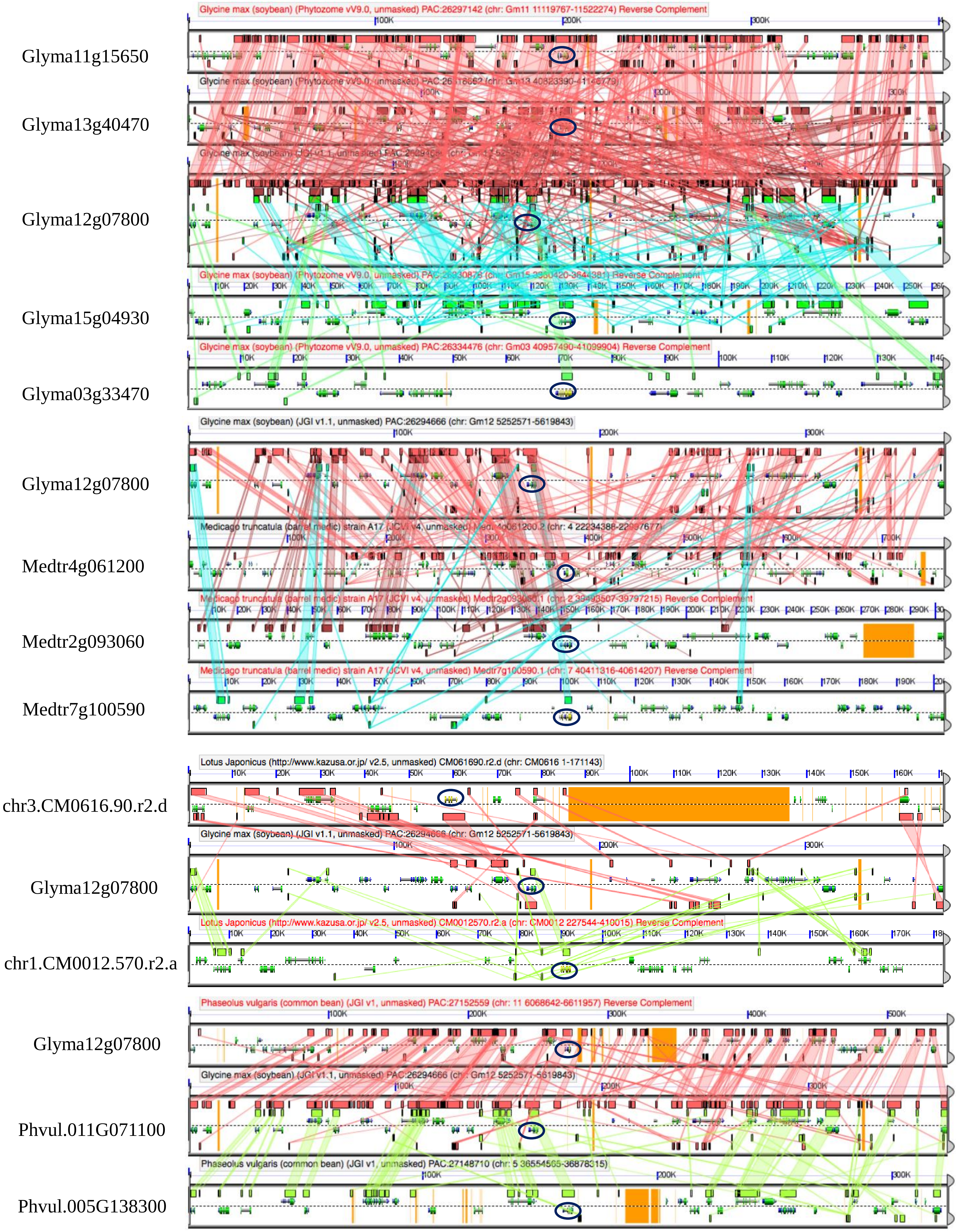

SINA1 orthologous genes in genomic regions of four legume plants

Glyma04g41410

Medtr1g008120

Glyma06g13440

Medtr3g091510

Medtr1g008120

Medtr1g008120

Phvul.009G052500

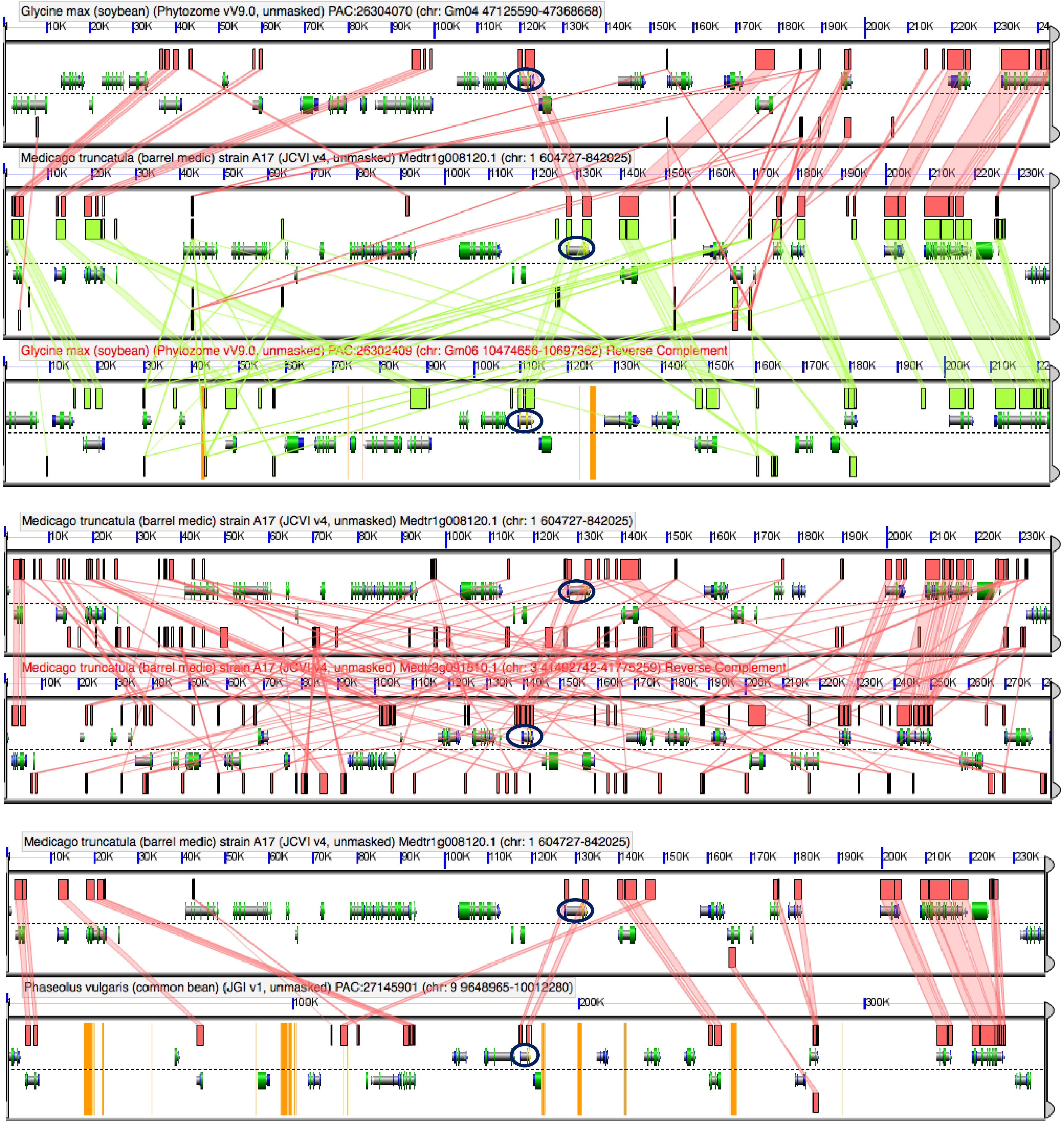

RabA2 orthologous genes in genomic regions of four legume plants

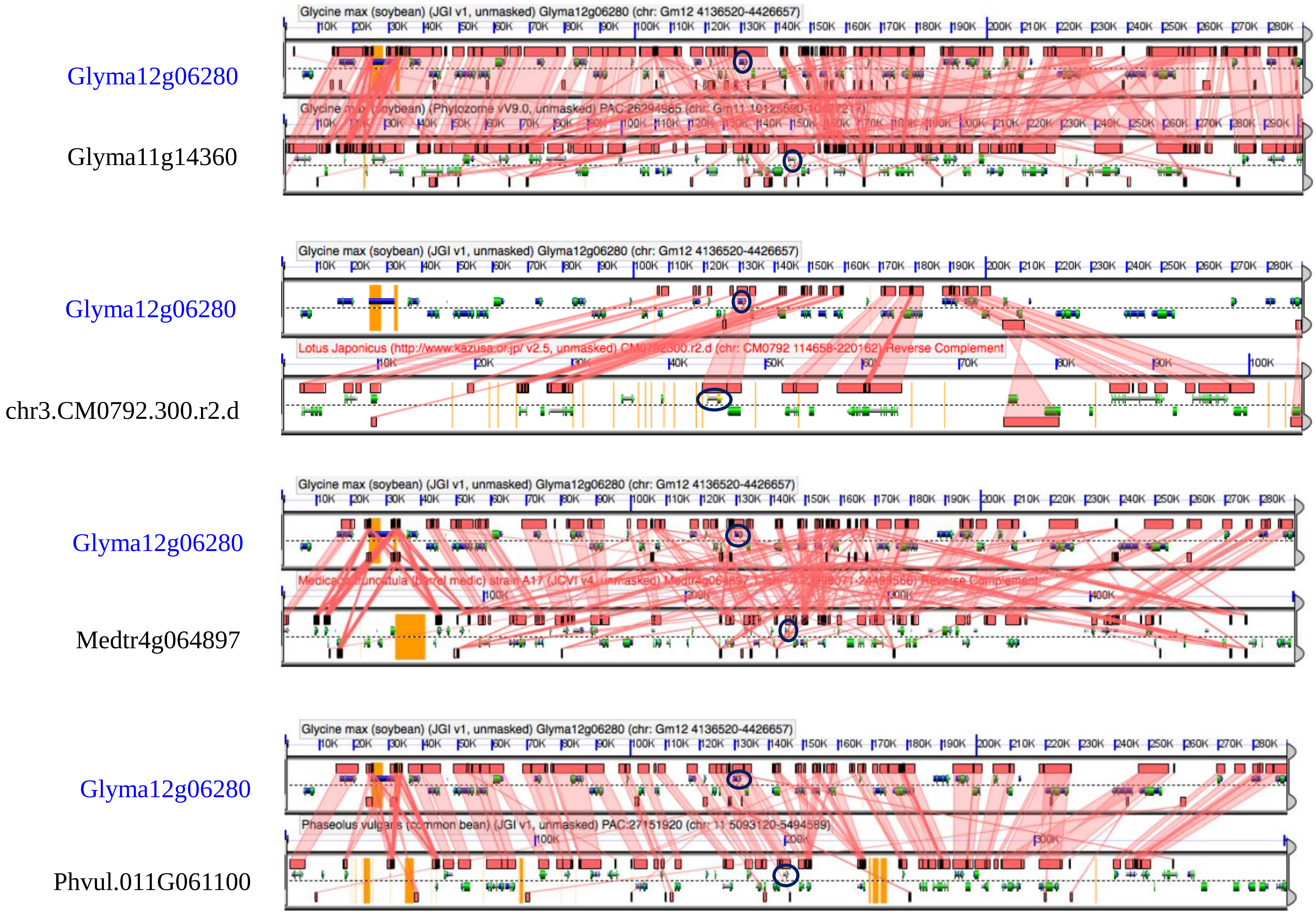

Supplement: Supplementary file 5 [file Presentation1.PDF]
